# Biomarker and genomic analyses reveal molecular signatures of non-cardioembolic ischemic stroke

**DOI:** 10.1038/s41392-023-01465-w

**Published:** 2023-05-30

**Authors:** Lingling Ding, Yu Liu, Xia Meng, Yong Jiang, Jinxi Lin, Si Cheng, Zhe Xu, Xingquan Zhao, Hao Li, Yongjun Wang, Zixiao Li

**Affiliations:** 1 Department of Neurology, Beijing Tiantan Hospital, Capital Medical University, Beijing, 100070 China; 2 China National Clinical Research Center for Neurological Diseases, Beijing, 100070 China; 3 Research Unit of Artificial Intelligence in Cerebrovascular Disease, Chinese Academy of Medical Sciences, Beijing, 100070 China; 4 Center for Excellence in Brain Science and Intelligence Technology, Chinese Academy of Sciences, Shanghai, 200031 China; 5 Advanced Innovation Center for Human Brain Protection, Capital Medical University, Beijing, 100070 China; 6 Clinical Center for Precision Medicine in Stroke, Capital Medical University, Beijing, 100070 China; 7 Chinese Institute for Brain Research, Beijing, China; 8 Beijing Engineering Research Center of Digital Healthcare for Neurological Diseases, Beijing, 100070 China

**Keywords:** Prognostic markers, Cardiovascular diseases, Genome informatics

## Abstract

Acute ischemic stroke (AIS) is a major cause of disability and mortality worldwide. Non-cardioembolic ischemic stroke (NCIS), which constitutes the majority of AIS cases, is highly heterogeneous, thus requiring precision medicine treatments. This study aimed to investigate the molecular mechanisms underlying NCIS heterogeneity. We integrated data from the Third China National Stroke Registry, including clinical phenotypes, biomarkers, and whole-genome sequencing data for 7695 patients with NCIS. We identified 30 molecular clusters based on 63 biomarkers and explored the comprehensive landscape of biological heterogeneity and subpopulations in NCIS. Dimensionality reduction revealed fine-scale subpopulation structures associated with specific biomarkers. The subpopulations with biomarkers for inflammation, abnormal liver and kidney function, homocysteine metabolism, lipid metabolism, and gut microbiota metabolism were associated with a high risk of unfavorable clinical outcomes, including stroke recurrence, disability, and mortality. Several genes encoding potential drug targets were identified as putative causal genes that drive the clusters, such as *CDK10, ERCC3*, and *CHEK2*. We comprehensively characterized the genetic architecture of these subpopulations, identified their molecular signatures, and revealed the potential of the polybiomarkers and polygenic prediction for assessing clinical outcomes. Our study demonstrates the power of large-scale molecular biomarkers and genomics to understand the underlying biological mechanisms of and advance precision medicine for NCIS.

## Introduction

Acute ischemic stroke (AIS) is a heterogeneous syndrome characterized by a high risk of recurrence, mortality, and disability; hence, reducing the burden of ischemic stroke remains a global challenge.^1,2^ Non-cardioembolic ischemic stroke (NCIS), which constitutes the majority of AIS cases, is mainly caused by arteriosclerosis and atherosclerosis.^3,4^ Despite the guidelines for secondary prevention and optimal control of risk factors, the 10-year risk of stroke recurrence is ~27% in patients with NCIS, indicating the urgent need for new therapeutic strategies.^4,5^ Recently, increasing evidence on the pathophysiological mechanisms of NCIS has emerged, which can facilitate the development of new therapy strategies.^6,7^ However, it remains a challenge to stratify patients with NCIS using molecular biomarkers, and personalized treatment strategies are lacking.

Circulating biomarkers can be used to discern the biological and pathological mechanisms of NCIS. Lipids, glycemic traits, and liver and kidney function tests are frequently used to monitor disease conditions and are key prognostic factors for AIS.^8^ In particular, inflammation and gut microbial metabolites are emerging nontraditional risk factors.^6,7,9^ Although the genetic basis of some biomarkers has been extensively studied,^10,11^ most biomarkers have not been well defined in large-scale ischemic stroke-affected population datasets. In addition, there remains a lack of comprehensive insight into the mechanism underlying the biological heterogeneity of NCIS. Deep phenotypic profiling based on circulating biomarkers and genetic data can expand our understanding of the specific characteristics of NCIS and aid in the development of new therapies.^12^

A major challenge in translating molecular findings into therapeutic innovations is reclassifying patients with stroke into subpopulations based on molecular biomarkers. Despite advances in bioinformatics, capturing the heterogeneous phenotypes of populations with distinct molecular mechanisms, therapeutic effects, and prognoses remains difficult. Machine learning approaches are particularly useful when dealing with complex, high-throughput, and multidimensional biomedical data and can be examined to identify further biological associations. In particular, dimensionality reduction and clustering analysis are unbiased, hypothesis-free, data-driven approaches that facilitate the identification of homogeneous subclusters within a heterogeneous dataset; this approach is becoming increasingly popular in precision medicine to reclassify phenotypes of complex diseases and probe common biomolecular interactions and pathophysiological patterns that correlate with diseases.

In the Third China National Stroke Registry (CNSR-III) study, we collected 59 clinical phenotypes, 63 circulating biomarkers, and whole-genome sequence data from 7,695 individuals with NCIS. We applied a data-driven dimensionality reduction approach to identify the biologically and clinically relevant subpopulations. This study aimed to establish evidence of biological and genetic heterogeneity among these heterogeneous subpopulations of NCIS by integrating genomic data with circulating biomarker data, thereby facilitating the development of high-precision treatment strategies and serving as a theoretical basis for future precision medicine studies using molecular profiling to guide individualized therapy in patients with AIS.

## Results

**Fig. 1:**
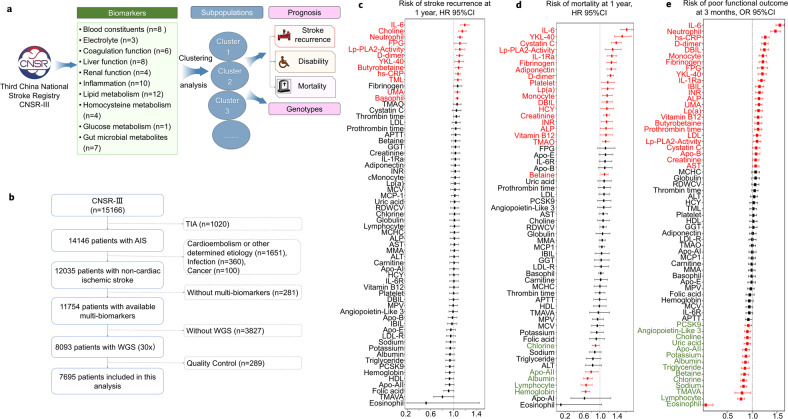
Study design and analysis of 63 biomarkers for non-cardioembolic ischemic stroke (NCIS). **a** Research framework. **b** Study flow. **c** Risk of stroke recurrence for 63 circulating biomarkers. Hazard ratio (HR), 95% confidence interval (CI) were calculated by adjusted Cox proportional hazards regression. **d** Risk of mortality at 1 year, assessed with 63 circulating biomarkers. HR (95% CI) were calculated by adjusted Cox proportional hazards regression. **e** Risk of poor functional outcome (defined as a modified Rankin Scale (mRS) score >2) at 3 months using 63 circulating biomarkers. Adjusted odds ratios were calculated using logistic regression. All models were adjusted for sex, age, alcohol consumption, smoking, history of stroke, dyslipidemia, hypertension, diabetes mellitus, and coronary heart disease. (Red line: *P* < 0.05)

We analyzed data from the Third China National Stroke Registry (CNSR-III), a nationwide, multicenter, prospective observational registry study of patients with AIS or transient ischemic attack (TIA) enrolled at 201 hospitals in China between August 01, 2015 and March 31, 2018. The mean age of the patients was 62.2 (SD = 11.3) years and 31.7% of the patients were female. To explore the biology of ischemic stroke and identify new therapeutic opportunities, we performed a comprehensive molecular and genomic characterization of these patients. Patients with TIA, cardioembolism (CE), or stroke caused by a specific etiology such as Moyamoya disease, Fabry disease, and other uncommon diseases that had specific biomarkers and genetic variants were excluded from the analysis. Patients who presented with cancer or infection before stroke, those without multiple circulating biomarkers, and those who did not undergo whole-genome sequencing (WGS) were also excluded. After data quality control of WGS (30× coverage), 7,695 individuals with NCIS were included in this study (Fig. 1a, b, Supplementary Table 1).

### Prognostic biomarkers for NCIS

To profile the molecular characteristics and prognostic markers in NCIS, we excluded seven biomarkers that were missing in 25% or more of the samples and 11 biomarkers with correlation coefficients >0.7. A total of 63 circulating biomarkers were included in this study, including electrolytes (*n* = 3), blood constituents (*n* = 8), coagulation function (*n* = 6), liver function (*n* = 8), renal function (*n* = 4), inflammation (*n* = 10), lipid metabolism (*n* = 12), homocysteine metabolism (*n* = 4), gut microbial metabolites (*n* = 7), and glucose metabolism (*n* = 1) (Fig. 1a, Supplementary Tables 2-3).

We performed cumulative risk assessments for each biomarker and identified 12 high-risk biomarkers for stroke recurrence in the population, including the inflammatory factors interleukin-6 (IL-6), Lp-PLA2-activity, high-sensitivity C-reactive protein (hs-CRP), neutrophils, chitinase-3-like protein 1 (YKL-40), and basophils; D-dimer; fasting plasma glucose (FPG); the gut microbial metabolites choline, butyrobetaine, and trimethyllysine (TML); renal function index (UMA); and the four low-risk biomarkers albumin, apolipoprotein AII, folic acid, and N,N,N-trimethyl-5-aminovaleric acid (TMAVA). In addition, we identified 25 biomarkers significantly (*P* < 0.05) associated with a high risk of poor functional outcome (modified Rankin Scale (mRS) 3–6) and 19 biomarkers significantly (*P* < 0.05) associated with a high risk of mortality (Fig. 1c–e).

### Hierarchical clustering identifies molecular clusters with prognostic relevance

**Fig. 2:**
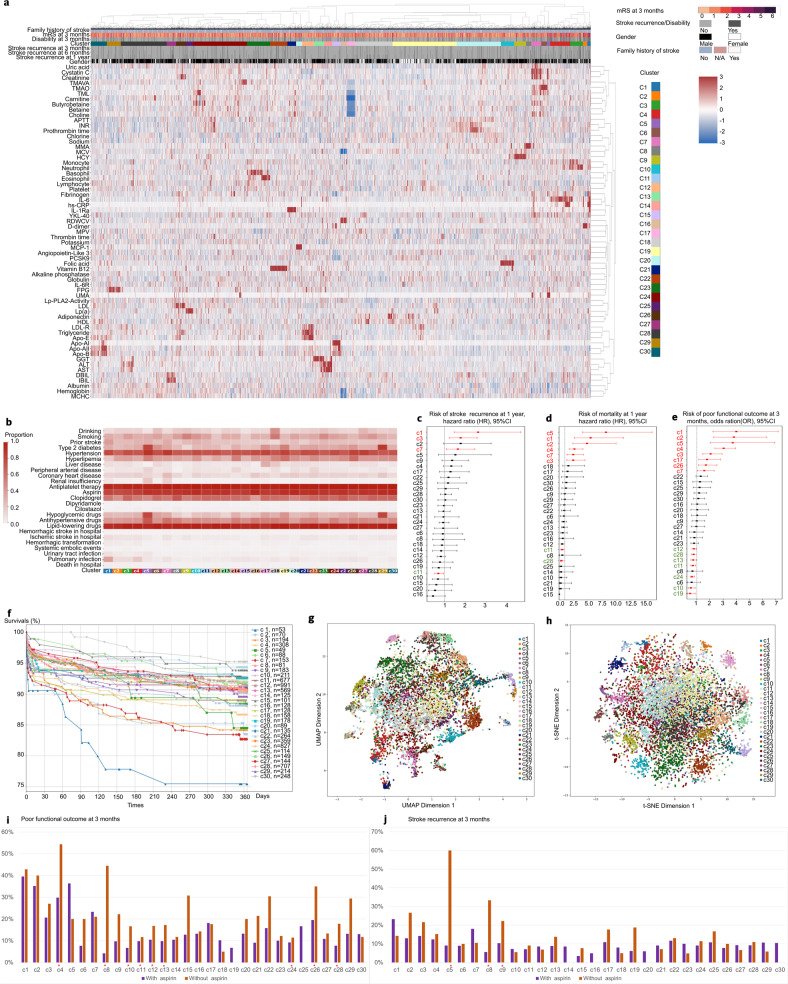
Clustering analysis reveals subpopulations associated with clinical phenotypes and outcomes of non-cardioembolic ischemic stroke (NCIS). **a** A heatmap illustrating 30 clusters based on 63 circulating biomarkers. **b** Characterization of clinical phenotypes and medication use across clusters. **c** Cumulative risk of stroke recurrence at 1 year for the 30 clusters. **d** Cumulative risk of mortality at 1 year for the 30 clusters. **e** Cumulative risk of poor functional outcome at 3 months for the 30 clusters. **f** Kaplan–Meier curves of time to stroke recurrence within 1-year post-stroke for the 30 clusters. **g** Uniform manifold approximation and projection (UMAP) of 30 molecular clusters. **h** t-distributed stochastic neighbor embedding (t-SNE) of 30 molecular clusters. **i** Incidence of poor functional outcome in patients with or without aspirin therapy (^*^P < 0.05). **j** Incidence of stroke recurrence in patients with or without aspirin therapy (^*^P < 0.05)

To determine whether the stroke-affected population can be risk-stratified based on specific circulating biomarkers, we used hierarchical clustering by Euclidian distance to reveal molecular clusters and found that these individuals could be grouped into 30 clusters based on 63 biomarkers (Fig. 2a). The biomarkers that were enriched in each cluster and differed significantly from those in other clusters are shown in Table 1.

**Table 1:** Clinical characteristics of patients in 30 clusters

Cluster	Total number	Female (%)	Marked biomarker	Risk factors and family history	Comorbidity and complications	Etiology	Prognosis
**C1**	53	22.64%	hs-CRP	History of stroke (39.6%)	Pulmonary infection (37.7%)	LAA (35.8%)	High risk
**C2**	70	28.57%	D-dimer		DVT (5.7%)	LAA (42.9%)	High risk
**C3**	194	23.20%	Monocytes, neutrophils			LAA (35.1%)	High risk
**C4**	308	26.95%	IL-6	Age (median 67, IQR 60–76)	Pulmonary infection (18.8%), hemorrhagic transformation (3.9%)	LAA (38.6%)	High risk
**C5**	49	34.69%	UMA	History of stroke (30.6%), type 2 diabetes (81.6%), hypertension (89.8%), family history of diabetes (16.3%), hypertension (30.6%), and stroke (20.4%)	Urinary tract infection (20.4%), renal insufficiency (6.1%)		High risk
**C6**	88	18.18%	TMAO	Liver disease (11.4%)		SAO (36.4%)	Moderate risk
**C7**	153	20.92%	Cystatin C, creatinine	Age (median 68, IQR 61–76) history of stroke (32.7%), hypertension (86.9%), CHD 24.8%	Urinary tract infection (15%), renal insufficiency (0.7%)	LAA (33.3%)	High risk
**C8**	81	27.16%	MMA	Family history of hypertension (28.4%)		UE (54.3%)	Moderate risk
**C9**	183	7.65%	HCY	Smoking (52.5%)			Moderate risk
**C10**	211	18.96%	Folic acid			UE (55%)	Low risk
**C11**	677	16.54%	APTT, INR, prothrombin time			SAO (30.9%)	Low risk
**C12**	991	54.79%	Adiponectin HDL				Low risk
**C13**	569	36.03%	Adiponectin, HDL, YKL-40	BMI (median 2.67, IQR 21.78–25.53)			Low risk
**C14**	125	24.80%	TML,^a^ carnitine,^a^ butyrobetaine,^a^ betaine,^a^ choline^a^			SAO (34.4%)	Moderate risk
**C15**	101	56.44%	RDWCV, MCV,^a^ hemoglobin,^a^ MCHC^a^			LAA (34.7%)	Moderate risk
**C16**	128	39.06%	Apolipoprotein-AI			UE (61.7%)	Low risk
**C17**	128	37.50%	ALT, AST	Liver disease (32%)		UE (50.8%)	High risk
**C18**	158	9.49%	GGT	Smoking (57%), drinking (45.6%), Liver disease (19.6%)			Moderate risk
**C19**	178	44.38%	LDL-R, apo-E, triglyceride	Hyperlipemia (59.6%)		SAO (31.5%)	Low risk
**C20**	89	33.71%	MCP-1			LAA (33.7%)	Moderate risk
**C21**	135	35.56%	IL-1Ra				Moderate risk
**C22**	264	31.44%	Vitamin B12	PAD (13.3%)		LAA (30.3%)	High risk
**C23**	359	20.06%	Basophils, eosinophils	Smoking (47.9%)			Moderate risk
**C24**	827	22.37%	TMAVA, TML				Low risk
**C25**	114	41.23%	Lp(a)			UE (52.6%)	Moderate risk
**C26**	149	42.28%	LDL	Hyperlipemia (62.4%)		LAA (32.2%)	High risk
**C27**	144	9.72%	DBIL, IBIL			LAA (31.9%)	Moderate risk
**C28**	707	16.55%		BMI 25.39 (23.56, 27.60)			Low risk
**C29**	214	37.38%	FPG	Type 2 diabetes (86.9%), family history of diabetes (20.6%)		UE (50%)	Moderate risk
**C30**	248	41.13%	Apo-AII, apo-B				Moderate risk

*hs-CRP* high-sensitivity C-reactive protein, *IL-1Ra* Interleukin-1 receptor antagonist, *IL-6* interleukin-6, *UMA* urine microalbumin, *TMAO* trimethylamine-N-oxide, *MMA* methylmalonic aciduria, *HCY* homocysteinemia, *APTT* activated partial thromboplastin time, *INR* international normalized ratio, *HDL* high-density lipoprotein, *YKL-40* chitinase-3-like protein 1, *TML* trimethyllysine, *RDWCV* coefficient of variation of RBC distribution width, *MCV* mean corpuscular volume, *MCHC* mean corpuscular hemoglobin concentration, Apo-AI apolipoprotein-AI, *ALT* alanine aminotransferase, *AST* aspartate aminotransferase, *GGT* γ-Glutamyl transpeptidase, *LDL-R* low-density lipoprotein receptor, *Apo-E* apolipoprotein-E, *MCP-1* monocyte chemoattractant protein-1, *TMAVA* N,N,N-trimethyl-5-aminovaleric acid, *LP(a)* lipoprotein (a), *LDL* low-density lipoprotein, *DBIL* direct bilirubin, *IBIL* indirect bilirubin, FPG fasting plasma glucose, *Apo-AII* apolipoprotein AII, *Apo-B* apolipoprotein-B, *IQR* interquartile range, *CHD* coronary heart disease, *BMI* body mass index, *PAD* peripheral arterial disease, *DVT* deep venous thrombosis, *LAA* large artery atherosclerosis, *UE* undetermined etiology, *SAO* small artery occlusion

^a^ Low serum level

To further understand the classification, we compared the differences in clinical phenotypes in these clusters. We found that the risk factors and comorbidity were diverse across 30 clusters (Fig. 2b, Table 1, Supplementary Table 4). Cluster 5 (marked by UMA) and cluster 29 (marked by FPG) had a large proportion of patients with type 2 diabetes (81.6% and 86.9%, respectively) and those receiving hypoglycemic drugs (79.6% and 80.4%, respectively). Cluster 18 (marked by GGT) showed a high prevalence of drinking (45.6%). Cluster 17 (marked by AST and ALT) and cluster 18 had a large proportion of patients with liver disease. Cluster 1 (marked by hs-CRP) showed the highest risk of pulmonary infection (37.7%). Cluster 5 and cluster 7 (marked by cystatin C and creatinine) showed a high risk of urinary tract infection (20.4% and 15%, respectively). Cluster 1 and cluster 4 (marked by IL-6) showed a relatively high risk of hemorrhagic transformation in the hospital.

The identified subpopulations had different risks of clinical outcomes, indicating that distinct molecular profiles played a significant role in the pathophysiology and prognosis of ischemic stroke. There were several important biological subpopulations within the dataset that showed a high risk of unfavorable outcomes. These subpopulations were defined as clusters 1, 4, 5, 7, 17, and 26. Two of these subpopulations were characterized by inflammatory factors such as hs-CRP of cluster 1 and IL-6 of cluster 4. Renal function characterized the subpopulations of cluster 5, identified by UMA, and cluster 7, identified by cystatin C and creatinine. Cluster 17, identified by aspartate aminotransferase (AST) and alanine aminotransferase (ALT), was characterized by liver function. Cluster 26, identified by low-density lipoprotein (LDL), was characterized by lipid metabolism. Conversely, the subpopulations characterized by folic acid (cluster 10), apolipoprotein-E (Apo-E) (cluster 19), and prothrombin time (cluster 11) had a relatively low risk of unfavorable outcomes (Fig. 2c–f, Table 1, and Supplementary Tables 5–11).

### Dimensionality reduction of biomarkers reveals fine-scale population structures in NCIS

Our initial hierarchical clustering approach revealed important clusters defined on a molecular basis; however, hierarchical clustering did not reveal relationships between individuals across clusters. To further investigate the heterogeneity of subpopulations at greater resolution, identified by circulating biomarkers, we visualized the biomarkers using uniform manifold approximation and projection (UMAP) and t-distributed stochastic neighbor embedding (t-SNE) (Fig. 2g, h). We observed an overlap of the biomarker components and clusters derived from hierarchical clustering, which was consistent with the molecular profiles that classified the subpopulations. In the low-dimensional space, the similar data points were positioned closer together. We found that biomarkers of the same functional modules were adjacent in space. For example, the subpopulations associated with inflammation, such as cluster 1 (identified by hs-CRP) and cluster 4 (identified by IL-6), were adjacent to each other. The subpopulations characterized by renal function biomarkers, UMA (cluster 5), cystatin C, and creatinine (cluster 7), were also located adjacent to each other. Similarly, the subpopulations characterized by lipid metabolism, cluster 16 (Apo-AI) and cluster 19 (Apo-E), were located adjacent to each other (Supplementary Figs. 1–3). The low-dimensional embedding of biomarkers indicated that the subtle structural differences of AIS population can be determined based on the specific biomarker in the reduced dimension.

### Subpopulations capture specific genetic variations and pathways associated with NCIS

**Fig. 3:**
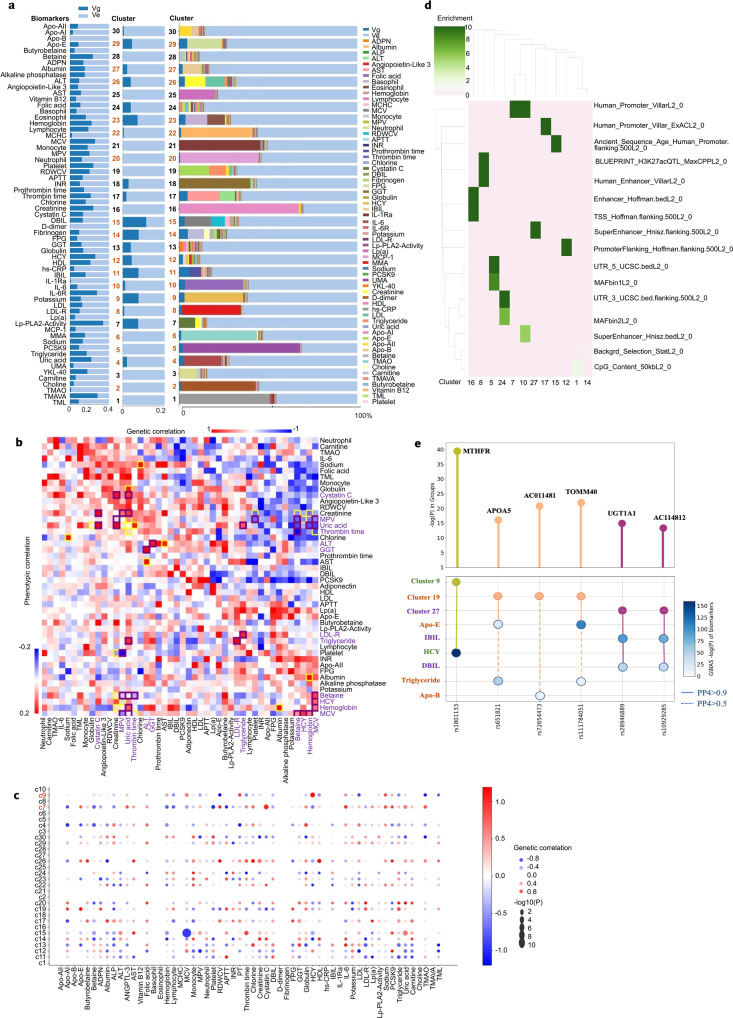
Genetic characteristics of the 30 molecular clusters identified in the cohort. **a** Proportion of variation attributed to the genotyped single-nucleotide polymorphisms (SNPs) and biomarkers for each cluster. **b** Genetic correlation between biomarkers (yellow box: *P* < 0.01, purple: FDR < 0.05). **c** Genetic correlations of the molecular clusters with the biomarkers. **d** Heritability and functional enrichment of the molecular clusters. **e** Colocalization of the molecular clusters and biomarkers

Next, we sought to understand the common genetic basis for variation in molecular clusters. Biomarkers and genome-wide single nucleotide polymorphism (SNP) data were used to estimate the proportion of variation attributable to each cluster. Common genetic variants accounted for 0–13.58% of the interindividual variation within clusters (Fig. 3a, Supplementary Table 12). To identify variants specific for each molecular cluster, we conducted genome-wide association studies (GWAS) across 30 clusters and performed pathway enrichment analysis of biological terms and gene ontology (GO) biological processes for each cluster. We identified 36 loci for 30 molecular clusters that satisfied a genome-wide significance threshold of *P* = 5.0 × 10^−8^ (Supplementary Table 13). After applying multiple testing correction to the number of clusters, 20 loci showed significant associations (*P* < 5.0 × 10^−8^/30 = 1.67 × 10^−9^). Seven SNPs were predicted as “deleterious” or “damaging” in missense variants with *P*-values < 1 × 10^−5^ (Supplementary Table 14). For example, CLCN6 (rs1023252, SIFT score = 0, deleterious_low_confidence) and C1orf167 (rs61773952, SIFT score = 0.01, deleterious; PolyPhen-2 score = 0.974, probably damaging) could affect protein function in cluster 9, which has been implicated in cardiovascular disease.^13^ The genes in cluster 9 were enriched for positive regulation of the apoptotic process [GO: 0043065]. The rs4148323 locus (UGT1A1, SIFT score = 0.05, PolyPhen-2 score = 0.625, possibly damaging) could affect protein function in cluster 27, which encodes UDP-glucuronosyltransferase involved in the glucuronidation pathway that transforms small lipophilic molecules, such as bilirubin and drugs. Cluster 27 enriched with genes for flavonoid glucuronidation [GO: 0052696] and aspirin ADME [R-HSA-9749641].

### Heritability and genetic correlations among biomarkers and subpopulations

The genetic effects on circulating biomarkers could offer novel insights into the mechanisms underlying the genetics of molecular clusters and relevant phenotypes. Therefore, we estimated the SNP-based heritability to further understand the molecular cluster mechanisms, computed pairwise genetic correlations (r_g_) across 30 molecular clusters and 63 biomarkers using bivariate linkage disequilibrium score regression (LDSC) (Fig. 3b, c), and identified shared genetic backgrounds for correlated biomarkers and molecular clusters. For example, cluster 7 was genetically correlated with cystatin C (r_g_ = 1; *P* = 0.005), cluster 9 was genetically correlated with homocysteinemia (HCY) (r_g_ = 1; *P* = 0.006), and cluster 15 had genetic correlations with mean corpuscular volume (MCV) (r_g_ = -0.923; *P* = 6.81 × 10^−11^) and hemoglobin (r_g_ = -0.469; *P* = 0.030). Heritability analysis suggests that clusters share, in part, a common genetic basis with biomarkers.

To identify the heritability enrichment in functional elements across cluster, we applied a stratified LDSC to test for annotation-specific heritability enrichment. Partitioning of functional genomic elements showed enrichment of heritability in regulatory elements, including CpG for cluster 1, histone H3 lysine 27 acetylation (H3K27ac) for cluster 8, 5' UTR for cluster 5, 3' UTR for cluster 24, transcription start site (TSS) for cluster 16, and super-enhancers for clusters 10 and 27 (Fig. 3d). Functional enrichment analysis revealed a significant contribution of conserved and regulatory regions to AIS.

### Colocalization of biomarker quantitative trait locus with GWAS risk loci identified

**Fig. 4:**
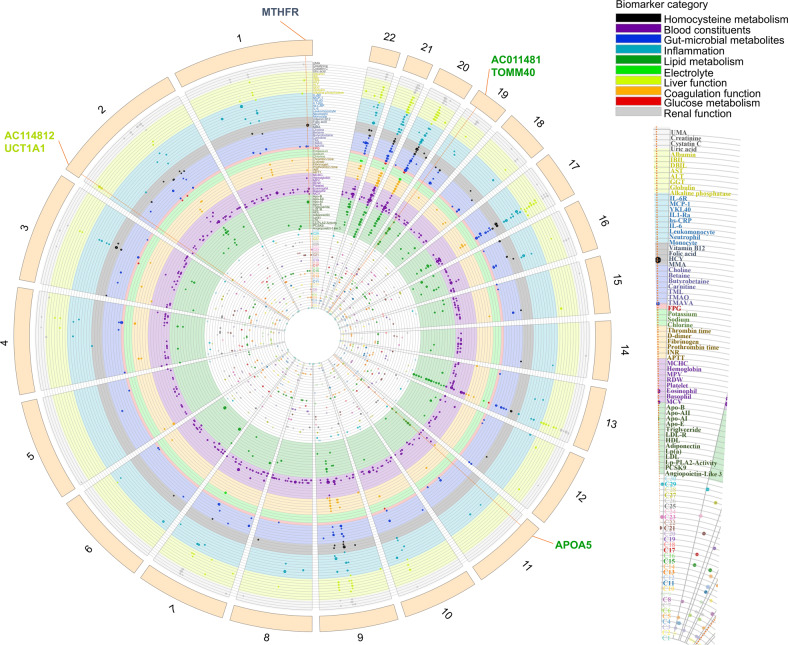
Genetics of 30 molecular clusters and 63 biomarkers. Circos plot showing the genome-wide significant variants for 30 molecular clusters (*P* < 1 × 10^−5^) and 63 biomarkers (*P* < 5 × 10^−8^). Each dot corresponds to a trait-associated locus, while each radial line connects dots for colocalization of biomarker-QTLs with cluster-GWAS risk loci

Genetic effects on circulating biomarkers may offer novel insights into the mechanisms underlying the genetics of molecular clusters. Through colocalization with clusters, biomarker quantitative trait locus (QTLs) may help identify causal genes and disease pathways. We obtained GWAS summary statistics for 63 biomarkers and 30 clusters and scanned all genome-wide significant GWAS loci overlapping in our results (Fig. 4). At a more relaxed genome-wide significant threshold (*P* < 1 × 10^−5^), nine GWAS loci with high support (PP4 > 0.8) and five with medium support (0.5 < PP4 ≤ 0.8) were identified in the colocalization between 63 circulating biomarkers and 30 molecular clusters.

Among the high- and medium-confidence (PP4 > 0.8 and PP4 > 0.5, respectively) colocalization results, we identified the genetic effects of circulating biomarkers and relevant molecular clusters. For example, rs1801133 (*MTHFR*) was identified as a candidate causal variant that explained the shared association signal between cluster 9 and *HCY*. In addition, we observed more than one colocalized QTL converging on multiple biomarkers for a molecular cluster. The pleiotropic loci included, for example, rs111784051(*TOMM40*) and rs651821 (*APOA5*) loci, both of which were associated with the serum levels of TG and Apo-E, and colocalized with GWAS signals for cluster 19 (marked by LDL-R, Apo-E, and triglyceride). Similarly, rs10929285 (*AC114812*) and rs28946889 (*UGT1A1*) both regulated the serum levels of indirect bilirubin (IBIL) and direct bilirubin (DBIL) and colocalized with GWAS signals for cluster 27 (marked by IBIL and DBIL) (Fig. 3e and Supplementary Table 15).

### TWAS-significant genes associated with NCIS subpopulations

To gain additional insights into the genetic basis underlying molecular clusters and search for driver-mediating genes that could act as candidate therapeutic targets, we used transcriptome-wide association studies (TWAS) to predict tissue-specific gene expression levels for molecular clusters and biomarkers. For 30 molecular clusters, we identified 39 TWAS signals for gene expression levels that were significantly associated with one or more of the tissues (*P* < 1.0 × 10^−7^), particularly in the central nervous and cardiovascular systems (Supplementary Table 16, Supplementary Fig. 4a). We also found that transcriptomic profiles of whole blood provided supporting evidence for the role of specific genes that modulated multiple clusters. The strongest signal was observed in *TSG101*, which encodes a component of the ESCRT-I complex required for the sorting of endocytic ubiquitinated cargos into multivesicular bodies (cluster 26: PTWAS = 3.52 × 10^−16^; cluster 24: PTWAS = 1.22 × 10^−9^; cluster 30: PTWAS = 9.45 × 10^−9^).

To explore the genetic overlaps between the TWAS results for clusters and biomarkers, we investigated 183 TWAS signals for 63 biomarkers (*P* < 5.0 × 10^−8^). RC3H2 expression was associated with cluster 21 and IL-1Ra (cluster 21: PTWAS = 1.97 × 10^−15^; IL-1Ra: PTWAS = 2.38 × 10^−16^); RC3H2 may play an important role in inflammation.^14^ KCNJ13 expression was associated with both cluster 27 and biomarkers for the liver function index (cluster 27: PTWAS = 2.5 × 10^−10^; TBIL: PTAS = 2.87 × 10^−65^; DBIL: PTWAS = 2.25 × 10^−25^; IBIL: PTWAS = 1.23 × 10^−56^). *KCNJ13*, which encodes an inwardly rectifying potassium channel protein, is associated with the risk of coronary artery disease.^15^ RELT expression was associated with cluster 19 and LDL-R, triglyceride (cluster 19: PTWAS = 3.28 × 10^−8^; LDL-R: PTWAS = 1.23 × 10^−19^; Triglyceride: PTWAS = 4.78 × 10^−14^). The results revealed the GWAS loci that may directly affect gene expression and contribute to both biomarkers and subpopulations.

### Target gene identification through eQTL colocalization of TWAS signals provides evidence of causality

We performed TWAS fine-mapping using FOCUS to prioritize putative causal genes that drive the clusters, which were used to compute posterior inclusion probability (PIP) and estimate credible sets for genes at each TWAS region and relevant tissue types. We identified colocalized expression quantitative trait loci (eQTLs) in 34 regions, providing suggestive evidence of causal genes in molecular clusters. These eQTLs may provide insights into the biological pathways in and drug targets for NCIS populations. We identified several genes encoding potential drug targets from human protein atlas database, with variants that influenced the identified subpopulations. For example, the likely causal variant CDK10 (PIP = 0.597) was associated with cluster 8, while the likely causal variant ERCC3 (PIP = 0.669) was associated with cluster 14, and CHEK2 (PIP = 0.774) was associated with cluster 21 (Table 2).

**Table 2:** Causal posterior probabilities for genes in 90% credible sets for clusters

Cluster	Gene symbol	Tissue	Gene type	Chrom	Twas_z	PIP	Region
1	*PDPN*	nerve_tibial	protein_coding	1	4.6	0.756	1:12779560–1:14890557
1	*ZNF493*	brain_hypothalamus	protein_coding	19	−4.85	0.863	19:20905964–19:22732521
1	*ZNF85*	thyroid	protein_coding	19	3.75	0.716	19:20905964–19:22732521
4	*SFI1*	esophagus_muscularis	protein_coding	22	4.56	0.795	22:29652158–22:31439918
5	*RP11-524C21.2*	lung	lincRNA	6	4.81	0.83	6:22748670–6:23936167
6	*RP11-48O20.4*	adipose_subcutaneous	lincRNA	1	−4.3	1.0	1:158027412–1:159913048
7	*CHCHD4*	brain_putamen_basal_ganglia	protein_coding	3	−4.42	0.982	3:13070799–3:14816745
7	*TMEM43*	whole_blood	protein_coding	3	−3.5	0.704	3:13070799–3:14816745
7	*SULT1E1*	lung	protein_coding	4	−3.14	0.568	4:68854765–4:71049152
7	*FOXC1*	whole_blood	protein_coding	6	−4.73	0.901	6:100116–6:1452209
8	*SPATA33*	brain_cerebellum	protein_coding	16	−2.84	0.745	16:89041165–16:90292788
8	***CDK10***	thyroid	protein_coding	16	−1.93	0.597	16:89041165–16:90292788
9	*DRAXIN*	adipose	protein_coding	1	3.88	0.567	1:11778084–1:12778482
10	*C1orf127*	nerve_tibial	protein_coding	1	−5.2	0.754	1:11778084–1:12778482
10	*RP11-430B1.2*	skin_sun_exposed_lower_leg	lincRNA	15	−4.85	0.61	15:53069747–15:54508497
11	*PM20D1*	colon_transverse	protein_coding	1	−4.51	0.561	1:206074070–1:208410160
11	*IRF4*	esophagus_mucosa	protein_coding	6	−4.64	0.855	6:100116–6:1452209
11	*ABCC4*	cells_transformed_fibroblasts	protein_coding	13	5.1	0.868	13:96087558–13:97519115
14	***ERCC3***	brain_caudate_basal_ganglia	protein_coding	2	4.47	0.669	2:128034539–2:129473860
14	*MTRNR2L9*	liver	protein_coding	6	−4.35	0.581	6:61880512–6:63552727
14	*SETDB2*	brain_caudate_basal_ganglia	protein_coding	13	−4.75	0.851	13:49384134–13:51590433
15	*RP11-297J22.1*	spleen	lincRNA	2	−4.55	0.749	2:118367466–2:121303783
15	*SFRP4*	artery_aorta	protein_coding	7	−4.54	0.783	7:36213873–7:37554798
16	*NAP1L1*	brain_hippocampus	protein_coding	12	−4.48	0.535	12:73818454–12:76511314
17	*IGF2R*	breast_mammary_tissue	protein_coding	6	1.64	0.94	6:160580578–6:162169443
17	*SLC22A1*	adipose	protein_coding	6	4.27	0.857	6:160580578–6:162169443
18	*AC110771.1*	liver	protein_coding	4	−4.02	0.522	4:184929717–4:186909090
18	*TMEM116*	artery_aorta	protein_coding	12	−1.29	0.548	12:110336719–12:113263518
18	*RP3-462E2.5*	thyroid	lincRNA	12	−1.28	0.517	12:110336719–12:113263518
19	*ALG1L11P*	brain_caudate_basal_ganglia	pseudogene	8	−3.27	0.758	8:10463197–8:11278541
19	*RP11-1101K5.1*	nerve_tibial	lincRNA	8	−4.85	0.794	8:110484517–8:111850847
19	*AP000797.4*	brain_cerebellar_hemisphere	lincRNA	11	−4.64	0.654	11:116383348–11:117746741
19	*ZNF284*	brain_cortex	protein_coding	19	−2.15	1.0	19:44744147–19:46102697
20	*TAF9BP1*	brain_cerebellum	pseudogene	3	4.48	0.742	3:23804865–3:25461007
21	*NMT2*	muscle_skeletal	protein_coding	10	4.86	0.878	10:13321600–10:15026047
21	***CHEK2***	blood	protein_coding	22	3.59	0.774	22:27835627–22:29650993
24	*CRELD2*	adipose_subcutaneous	protein_coding	22	5.07	0.503	22:49825112–22:51240820
25	*LPAL2*	brain_dorsolateral_prefrontal_cortex	transcribed_unprocessed_pseudogene	6	5.0	0.712	6:160580578–6:162169443
26	*RP1L1*	lung	protein_coding	8	3.74	0.772	8:10463197–8:11278541
26	*NEIL2*	brain_amygdala	protein_coding	8	0.0443	0.594	8:10463197–8:11278541
26	*TMEM116*	artery_aorta	protein_coding	12	0.4	0.518	12:110336719–12:113263518
27	*C2orf82*	brain_hippocampus	protein_coding	2	−4.15	0.977	2:233550145–2:235150987
27	*C2orf82*	brain_amygdala	protein_coding	2	−6.02	0.764	2:233550145–2:235150987
27	*UGT1A6*	esophagus_mucosa	protein_coding	2	−5.54	0.673	2:233550145–2:235150987
27	*TTC39B*	adipose_subcutaneous	protein_coding	9	4.71	0.891	9:12276490–9:14836363
28	*TUB*	esophagus_muscularis	protein_coding	11	3.96	0.69	11:9087533–11:10951332
28	*TMEM41B*	adipose	protein_coding	11	1.55	0.597	11:9087533–11:10951332
29	*RPS10P7*	brain_caudate_basal_ganglia	lincRNA	1	4.5	0.743	1:200137649–1:201589075

Abbreviations: PIP posterior inclusion probability

### Landscape of NCIS subpopulations

Nine clusters with higher risk of stroke recurrence, mortality, or unfavorable functional outcomes when compared with other clusters were defined as high-risk subpopulations, while eight clusters with lower risk of stroke recurrence, mortality, or unfavorable functional outcomes when compared with other clusters were defined as low-risk subpopulations (Table 1). We mapped the phenotypic and genetic characteristics of these subpopulations to reveal their molecular landscapes and identify potential therapeutic targets for NCIS. The characteristics of several typical subpopulations are as follows:

### High-risk subpopulations

#### Cluster 1—inflammation

Cluster 1 was characterized by inflammation (hs-CRP). Patients in cluster 1 had a high prevalence of a history of stroke (39.6%), symptomatic intracranial atherosclerotic stenosis (sICAS) (39.6%), and pulmonary infection (37.7%) and a significantly higher risk of stroke recurrence (adjusted hazard ratio (HR) 2.601, 95% CI 1.467-4.611, *P* = 0.001), mortality (adjusted HR 5.495, 95% CI 2.695–11.204, *P* < 0.001) within 1 year as well as poor functional outcome within 3 months (adjusted HR 4.479, 95% CI 2.237–7.214, *P* < 0.001). ADAMTS9-AS2 (rs4688534, *P* = 2.52 × 10^−58^) was associated with cluster 1, which has been reported to be associated with infarct size, immune infiltration, and poor survival.^16,17^ In addition, PDPN explained most of the signal in its region (lead SNP *P*_GWAS_ = 3.93 × 10^−6^; conditioned on PDPN lead SNP *P*_GWAS_ = 0.118) (Supplementary Fig. 4b). For the genomic locus 1:12779560-1:14890557, PDPN was included in the 90% credible gene set, with a posterior probability of 0.756 (Table 2).

#### Cluster 2—D-dimer

Cluster 2 was characterized by a high D-dimer level, which suggested hypercoagulable states and increased thrombosis risk. The patients in cluster 2 had a high prevalence of deep venous thrombosis (5.7%), sICAS (41.4%) and symptomatic extracranial atherosclerotic stenosis (sECAS) (7.1%), a higher risk of mortality within 1 year (adjusted HR 4.867, 95% CI 2.394–9.895, *P* < 0.001), and poor functional outcome within 3 months (adjusted HR 3.743, 95% CI 2.300–6.347, *P* < 0.001). We identified 23 independent loci associated with cluster 2 at a genome-wide significance threshold of *P* < 1 × 10^−5^. GWAS loci (ICA1L-WDR12-CARF-NBEAL1) on chromosome 2 (rs10190966, lead SNP_GWAS_ *P* = 1.35 × 10^−7^, lead SNP_GWAS_ = 0.13) explained 0.918 of the variance in atrial appendages (Supplementary Fig. 4c). The ICA1L-WDR12-CARF-NBEAL1 locus was also found to be associated with lacunar stroke.^18^

#### Cluster 7—renal function

Cluster 7 was characterized by renal function (cystatin C and creatinine) levels and had a high prevalence of a history of stroke (32.7%), sICAS (33.34%), hypertension (86.9%), CHD (24.8%), urinary tract infection (15%), and renal insufficiency (0.7%). The patients in cluster 7 had a significantly higher risk of stroke recurrence (adjusted HR 1.6491, 95% CI 1.111–2.448, *P* = 0.013) and mortality (adjusted HR 2.433, 95% CI 1.400–4.227, *P* = 0.002) at 1 year and a high risk of poor functional outcome within 3 months (adjusted HR 2.014, 95% CI 1.052–2.312, *P* = 0.027) when compared with other clusters. We found that a non-coding transcript variant of the protein-coding gene *TMEM43* (rs6798807, *p* = 4.9 × 10^−8^) was significant in cluster 7. For the GWAS loci on chromosome 3, conditioning of *TMEM43* explained 0.924 of the variance (rs3796308 lead SNP_GWAS_ *P* = 4.9 × 10^−8^, lead SNP_GWAS_ = 0.133) (Supplementary Fig. 4d). For the genomic locus 3:13070799-3:14816745, FOCUS showed that *TMEM43* was included in the 90% credible gene set with a posterior probability of 0.704 in whole blood (Table 2).

#### Cluster 26—lipid metabolism

Cluster 26 was characterized by lipid metabolism (LDL, triglyceride). Patients in cluster 26 had a significantly higher risk of disability (adjusted HR 1.836, 95% CI 1.117–2.522, *P* = 0.013). *C8orf74* was associated with neurodevelopmental disorders (rs77073793, SIFT score = 0.04, PolyPhen-2 score = 0.833, possibly damaging) and could affect protein function in cluster 26. The genes in cluster 26 were enriched for cell-cell adhesion (GO: 0098609) and G-protein coupled receptor (GPCR) downstream signaling (R-HSA-388396). GPCRs expressed on the surface of platelets play key roles in the regulation of platelet activity and function. Pharmacological blockade of these receptors, including P2Y1 and P2Y12, can help to prevent arterial thrombosis.^19^ Aspirin was effective in reducing the risk of unfavorable functional outcomes in patients in cluster 26 (Fig. 2i, j).

For the genomic locus 8:10463197-8:11278541, *NEIL2* was included in the 90% credible gene set, with a posterior probability of 0.594 in the brain_amygdala. *NEIL2* is associated with DNA repair; the capacity for DNA repair is likely to be one of the factors that determines the neuronal vulnerability to ischemic stress and may influence the pathological outcome of stroke.^20^ The upregulated genes in cluster 26 showed a hallmark of Notch signaling, an important mediator of hepatic lipid metabolism and the remodeling of blood vessels.

### Low-risk subpopulations

#### Cluster 10—folic acid

Patients in cluster 10 (identified by folic acid) had a significantly lower risk of disability (HR 0.541, 95% CI 0.311–0.879, *P* = 0.014). *RC3H2* expression was associated with cluster 10 based on its expression in the colon_sigmoid (PTWAS = 8.15 × 10^−23^), while *TREH* expression was associated with cluster 10 based on its expression in the colon_sigmoid (PTWAS = 2.26 × 10^−12^) and adipose_subcutaneous (PTWAS = 7.18 × 10^−10^). For the genomic locus 1:11778084-1:12778482, *C1orf127* was included in the 90% credible gene set, with a posterior probability of 0.754 in the nerve_tibial.

#### Cluster 16—apolipoprotein A

Patients in cluster 16 (identified by Apo-AI and Apo-AII) had a relatively low risk of stroke recurrence at 3 months (HR, 0.119; 95% CI 0.017–0.850, *P* = 0.034). The expression of *MTMR14* (myotubularin-related protein 14), which is associated with lipid phosphatase, was associated with cluster 16 based on its expression in the heart_left_ventricle (PTWAS = 7.83 × 10^−8^) and whole blood (PTWAS = 7.93 × 10^−8^). For GWAS loci on chromosome 3, conditioning in *MTMR14* explained 0.349 of the variance (rs7618350 lead SNP_GWAS_ *P* = 1.86 × 10^−7^, lead SNP_GWAS_ = 2.60 × 10^−5^). For the genomic locus 12:73818454-12:76511314, *NAP1L1* was included in the 90% credible gene set, with a posterior probability of 0.535 in the brain_hippocampus.

#### Cluster 24—gut microbiota metabolism

Cluster 24 was characterized by gut microbial metabolites (TMAVA and TML) and had a relatively low risk of poor functional outcome (HR 0.675, 95% CI 0.493–0.808, *P* < 0.001). We found that *CRELD2*, which plays a role in calcium ion binding activity and protein disulfide isomerase activity (rs12170409, *P* = 2.25 × 10^−8^), was significantly associated with cluster 24. For the genomic locus 22:49825112-22:51240820, *CRELD2* was included in the 90% credible gene set with a posterior probability of 0.503 in adipose tissue. The genes in cluster 24 were enriched for the regulation of the Wnt signaling pathway (GO: 0016055).

### Biomarkers and SNP profiling for predicting clinical outcomes across the NCIS subpopulations

**Fig. 5:**
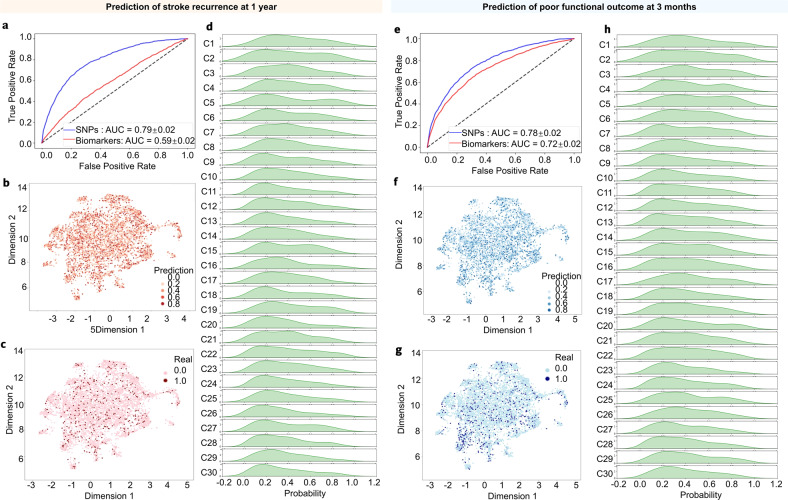
Prediction models for clinical outcomes across the subpopulations. **a** Area under the ROC curve (AUC-ROC) analysis was used to predict stroke recurrence at 1 year using top SNPs and biomarkers. UMAP labeled by (**b**) predicted probability and (**c**) incidence of stroke recurrence at 1 year. **d** Density plots depicting the predicted probability of stroke recurrence at 1 year across the subpopulations. **e** Area under the ROC curve (AUC-ROC) estimates for the prediction of poor functional outcome (mRS > 2) at 3 months using top SNPs and biomarkers. UMAP labeled by the (**f**) predicted probability of poor functional outcome and (**g**) incidence of poor functional outcome (mRS > 2) at 3 months. **h** Density plots depicting the predicted probability of poor functional outcome (mRS > 2) at 3 months across the subpopulations

To provide potential aid for clinicians, we examined the applicability of biomarkers and SNP panels in predicting stroke recurrence and poor functional outcomes, respectively. The top 500 SNPs associated with clinical outcomes and 63 biomarkers were selected to develop the prediction models. Using these 63 circulating biomarkers, the estimated receiver operating characteristic area under the curve (ROC AUC) of stroke recurrence at 1 year was 0.59, while that of poor functional outcome at 3 months was 0.72. When using SNP profiling, the model performance increased to 0.79 ± 0.02 in predicting the risk of stroke recurrence, with an AUC of 0.78 ± 0.02 in predicting the risk of poor functional outcome. The SNP-based prediction models showed a large improvement in the predictive ability compared with the biomarker-based prediction models. As depicted in density plots and UMAPs, the predicted probability of stroke recurrence and poor functional outcome were diverse across the 30 clusters. There was a high proportion of patients with high predicted probability of stroke recurrence and poor functional outcome in cluster 1 (identified by hs-CRP), cluster 2 (identified by D-dimer), and cluster 5 (identified by UMA) (Fig. 5).

## Discussion

In this study, we developed a new approach for examining the heterogeneity of NCIS by characterizing the molecular and genetic profiles of its subpopulations. Through a dimensional reduction approach, we identified 30 subpopulations based on 63 circulating biomarkers using data from 7695 patients with NCIS in the CNSR-III dataset and provided evidence of distinct molecular clusters characterized by different biological and pathogenic processes. We performed GWAS and TWAS analyses to assess the genetic associations with the molecular clusters and further explored their associations with circulating biomarkers, clinical phenotypes, and prognosis.

Our findings further clarify the important role of biomarkers for inflammation, homocysteine metabolism, liver and renal function, and gut microbiota metabolism in ischemic stroke. Subpopulations characterized by the inflammation indicators hs-CRP and IL-6 showed high risk of stroke recurrence, poor functional outcome, and mortality. Inflammatory markers such as hs-CRP and IL-6 are considered acute-phase reactants. We found that cluster 1 (characterized by hs-CRP) showed the highest risk of pulmonary infection. It should be noted that aspiration pneumonia may have contributed to the risk of adverse clinical outcomes, including stroke recurrence, disability, as well as mortality. Recently, anti-inflammatory agents, such as colchicine, have been shown to play a prominent role in improving cardiovascular outcomes in clinical trials.^21^ Our study supports anti-inflammatory treatment as a promising therapy for ischemic stroke. Using GWAS analysis, we identified *PDPN* in a subpopulation characterized by hs-CRP. Podoplanin (PDPN) is involved in lymphangiogenesis and platelet aggregation.^22^ Previous studies have also reported that the PDPN–CLEC-2 axis plays a role in the function of fibroblastic reticular cell, which is critical for controlling immune activation.^23^ In addition, the CLEC2-PDPN axis could be a target for inhibiting the interactions between platelets, the main cause of thrombosis.^24,25^

Kidney disease is a major risk factor for cerebrovascular disease.^26^ Additionally, renal insufficiency can lead to inflammation, oxidative stress, and abnormal calcium-phosphorus metabolism.^27,28^ Subpopulations identified by biomarkers of renal function, such as UMA, cystatin C, and creatinine, had a significantly high risk of stroke recurrence and mortality. We found that the expression of *TMEM43* was strongly correlated with the subpopulation characterized by renal function. *TMEM43* encodes a highly conserved integral membrane protein (transmembrane protein 43) and is associated with cardiac and metabolism-related pathways and diabetic kidney disease.^29,30^ Nevertheless, whether *TMEM43* is a potential therapeutic target for patients with NCIS remains to be confirmed.

The correlation between liver function and ischemic stroke remains unclear.^31,32^ Our study fills this critical gap by analyzing the distinct molecular signature and its related genetic variants. The subpopulation characterized by liver enzymes of AST and ALT was associated with high risk of unfavorable functional outcome. Another subpopulation (cluster 27) characterized by liver function indicators, IBIL and TBIL, was strongly associated with *UGT1A1*; this gene encodes UDP-glucuronosyltransferase and correlates with bilirubin level. We also found that genes^33^ in cluster 27 were enriched for flavonoid glucuronidation, a potential therapeutic agent in neurological protection.^34,35^

Homocysteine metabolism is relevant for various pathological conditions in AIS, especially ischemia-induced damage that can lead to the accumulation of reactive oxygen species (ROS).^13,36^ Our findings showed that populations characterized by homocysteine metabolism impairments, manifested as increased HCY level, had a moderate risk of unfavorable clinical outcomes, and that candidate loci were strongly associated with these subpopulations, including *MTHFR* and *CLCN6*. However, increased folate intake was associated with a reduced risk of poor functional outcome post-stroke, suggesting that increasing habitual folate intake may have a beneficial effect with respect to stroke incidence.^37,38^

We found the prognosis value of gut microbiota metabolisms in this NCIS population, especially in the subpopulation characterized by TMAVA and TML, which was associated with decreased risk of poor functional outcome. The microbial metabolite TMAO is linked to increased risk of cardiovascular risk.^39^ However, there is limited evidence for other microbial metabolites, such as TML and TMAVA. Increasing evidence suggests that gut microbiota products may promote post-stroke recovery.^9^ There may be interactions between lipids and gut microbiota metabolism; the gut microbiome has been implicated in the regulation of cholesterol homeostasis.^40^ A variant of *CRELD2* was associated with the subpopulation marked by gut microbiota metabolism, a novel chaperone for the receptor LDL receptor-related protein 1 (LRP1).^41^ *CRELD2* is also involved in the regulation of Wnt signaling pathway, which is important for the recovery of ischemic brain injury, reversal of blood-brain barrier (BBB) breakdown, and promotion of neurogenesis and angiogenesis.^41^ The Wnt signaling pathway may also be involved in microbiota-induced inflammation and immune homeostasis.^42^

Finally, limited research has been conducted on genetic associations with stroke-related outcomes. We constructed polygenic prediction models for stroke recurrence and poor functional outcomes, which may potentially aid clinicians to determine individual patient risks. Notably, the predicted probability of stroke recurrence and poor functional outcomes tended to be high in particular subpopulations. Most previous studies used selected biomarkers and genetic variants to predict risk of ischemic stroke in the AIS population.^43,44^ A recent study demonstrated that genetic risk scores were predictive of ischemic stroke independent of clinical risk factors in patients with cardiometabolic disease.^45^ Our results highlight the benefits of using SNP profiling panels for risk stratification in patients with NCIS.

In conclusion, we identified 30 molecular subpopulations based on 63 biomarkers and explored the potential biological heterogeneity of NCIS populations and constructed deep comprehensive landscape in NCIS. We found that biomarkers for inflammation, abnormal liver and kidney function, homocysteine metabolism, lipid metabolism, and gut microbiota metabolism can be used to stratify patients with AIS. Our findings provide novel insights into the molecular mechanisms underlying ischemic stroke and will help facilitate the development of novel therapeutic strategies against AIS.

### Study limitations

Our study has several limitations. First, apart from blood biomarkers and genomic data, our study lacked multi-omics data, such as transcriptome data, which would further delineate the molecular mechanisms of ischemic stroke. However, we used transcriptomic imputation from GTEx dataset and performed TWAS to bridge SNPs, genes, and phenotypes. Second, our clustering analysis strategy might have failed to characterize currently unidentified populations owing to biomarker limitations. Our analysis was based on many known risk factors that are important for prognosis or remain controversial in patients with NCIS. Although we identified multiple promising biomarker candidates, additional high-quality proteomic and metabolomic data may provide a robust approach to unravel novel molecular profiles underlying the complex phenotypes of ischemic stroke. Third, samples from different time points are required in future studies, which could potentially be utilized to explore the molecular dynamics during the disease course. Fourth, all participants in this study were from an East Asian population and thus, further studies are warranted to validate the applicability of our findings to populations of different ancestries. Finally, external validation is required to confirm the generalizability of our results, including the molecular signatures, biomarkers, and SNP profiling. Nevertheless, our results suggest that precision management based on molecular signatures should be considered in patients with NCIS.

## Materials and methods

### Study participants

The CNSR-III is a prospective, multicenter cohort of 15,166 patients with AIS or TIA recruited between August 01, 2015 and March 31, 2018, from 201 hospitals in China. The patients that participated in the CNSR-III study were at least 18 years old and admitted to the hospital within 7 days of AIS or TIA onset. Further details regarding the CNSR-III study design and methodology have been previously described.^46^ This study was approved by the Ethics Committees of Beijing Tiantan Hospital (IRB number: KY2015-001-01). Written informed consent was obtained from all participants or their representatives.

Patients who were diagnosed with TIA, cardioembolism (CE), or a stroke of other determined etiology were excluded from the analysis (*n* = 1651). Moreover, patients who presented with cancer (*n* = 100) or infection before stroke (*n* = 360), those without multiple circulating biomarkers (*n* = 281), and those without WGS data (*n* = 3827) were excluded.

### Clinical phenotypic data in CNSR-III

The clinical information of the patients was collected through in-person interviews by trained research coordinators. Stroke severity was assessed within 24 h of hospital admission using the National Institutes of Health Stroke Scale (NIHSS) score. Traditional stroke subtypes were classified into five major categories, namely large-artery atherosclerosis (LAA), small-vessel occlusion (SVO), cardioembolism (CE), undetermined etiology (UE), and other etiology (OE), according to the Trial of Org 10 172 in Acute Stroke Treatment (TOAST) criteria.^47^ Clinical outcomes, including recurrent stroke, all-cause mortality at 3, 6, and 12-months post-stroke, and poor functional outcome as defined by an mRS score >2 at 3-months post-stroke. Patients were followed up via in-person interviews at 3 months and via telephone interviews at 6 and 12 months by trained interviewers based on a standardized interview protocol to collect the clinical outcomes.^46^

### Blood sample collection and biomarker measurements

Blood samples were collected on the day of hospital enrollment and the median time from index event onset to sample collection was 55 h (IQR: 27–96 h). All specimens were stored at −80 °C until further analysis; 81 serum biomarkers identified in this study were extracted from these samples. Blood biomarker measurements were performed at the central laboratory of Tiantan Hospital, Beijing, China by laboratory staff who were blinded to the patients’ characteristics and clinical outcomes. The biomarkers that were missing in at least 25% of the samples and those with correlation coefficients >0.7 were excluded.

### WGS

Genomic DNA was extracted from peripheral white blood cells (WBCs) using a Magnetic Blood Genomic DNA Kit (DP329, TIANGEN Biotech Co. Ltd., Beijing, China) according to the manufacturer’s instructions. WGS was performed on the BGISEQ-500 platform (BGI Genomics, Shenzhen, China). The average depth was greater than 30× for each subject. Raw sequence reads were filtered using an in-house quality control pipeline. Samples were genotyped using MassARRAY®Typer (V.4.1, Agena Bioscience, California, USA). Details of the protocol have been described previously.^48^

### Genotyping and quality control

Standard genotyping of quality controls (QCs) was performed. Variants with call rate <98% and minor allele frequency (MAF) < 5% were excluded from further analysis. Samples with outlying heterozygosity for autosomal chromosomes (i.e., ±3 standard deviations away from the sample mean) were also excluded. Relatedness between individuals was assessed among all genotyped samples using an independent linkage disequilibrium-pruned subset of SNPs, leaving no pair with r^2^ > 0.2 within a window of 50 kb. No related samples were found.

Principal component analysis (PCA) was performed to identify large-scale differences in ancestry between individuals using PLINK.^49^ Genetic background outliers detected using PCA were filtered using 1000 G subjects as the population reference panel.^50^ After QC filters, 7695 samples were available for subsequent analyses.

### Unsupervised hierarchical clustering based on blood biomarkers

An unsupervised hierarchical clustering analysis was performed to identify clusters of individuals with NCIS based on 63 biomarkers using Ward’s hierarchical agglomerative clustering method (ward.d2) with the Euclidean distances of the z-scores.^51,52^ The resulting heatmap was visualized using Java TreeView software version 1.1.6r4. The reproducibility of clustering was assessed with the bootstrap resampling method using the Pvclust R package. The dendrogram for the clustering results shows the distance between each cluster using the Ward method, in which 30 clusters were identified. A key benefit of hierarchical clustering is its ability to create a tree structure with detailed division of every cluster. Thus, several clusters were obtained based on visual evaluation.

### Dimensionality reduction

UMAP is a novel nonlinear dimensionality reduction method that is capable of distinguishing neighboring clusters while retaining the high-dimensional topology of data points in the low-dimensional space. Visualization of the UMAP dimension reduction was based on a scatterplot in which each dot represents one sample and is labeled with biomarkers, clinical phenotypes, and outcomes.^53,54^ The t-distributed stochastic neighbor embedding (t-SNE) analysis assigns a weight to each of the modeling variables to create two-dimensional composite eigenvectors that represent gradients within the data. t-SNE identifies similar variable patterns between data points with multiple features^55^ and then maximizes the gradient of data by weighting the features to show similar data points near one another on the plot, while showing more different points further apart. In our study, we created two-dimensional t-SNE plots to represent the overall structure of our data. We colored the patients according to their phenotypic membership. These plots were created using Python 3.9 software.

### Estimating the proportion of genetic variation attributed to SNPs and biomarkers

The proportion of variation attributed to genotyped SNPs and biomarkers in each cluster was estimated using GCTA software with a linear mixed model (LMM).^56,57^ The 63 biomarkers were used as fixed effects in the regression analysis where Vg is the variance explained by SNPs and Vp is the total observed phenotypic variance. The variance explained by the 63 biomarkers was calculated from the estimation of the corresponding fixed effect coefficient. Each cluster was evaluated using a separate analysis.

### Genome-wide association study

We performed genome-wide association analyses of the 30 clusters. To enhance the biological insights into ischemic stroke, GWASs of 63 biomarkers and 59 clinical phenotypes in CNSR-III were also conducted. For clusters and binary phenotypes, we performed a first-fallback logistic regression using PLINK (v1.9). For biomarkers and quantitative phenotypes, we applied a generalized linear model association analysis using PLINK (v1.9). We applied quantile normalization for phenotype using the -pheno-quantile-normalize option, which fit a linear model with covariates and transformed the phenotypes to normal distribution N (0, 1) while preserving their original rank. We reported uncorrected association *P*-values from the linear regression and retained all SNP associations with uncorrected *P*-values of *P* < 10^−5^. Multiple hypothesis testing was accounted for by applying Bonferroni correction based on the number of studied traits, resulting in a genome-wide significance level of *P* < 1.67 × 10^−9^ (5.0 × 10^−8^/30) for clusters and *P* < 7.9 × 10^−10^ (5.0 × 10^−^8/63) for biomarkers. Each variant was annotated with a variant effect predictor (VEP). ‘Damaging’ variants were predicted using SIFT and PolyPhen-2. The PolyPhen-2 score ranged from 0.0 (tolerated) to 1.0 (deleterious), while the SIFT score ranged from 0.0 (deleterious) to 1.0 (tolerated).^58,59^ Novel variants were defined as those that were not indexed in any of the databases. We then performed GO enrichment analysis.

### Heritability and genetic correlation

We conducted LD score regression using LDSC software (v.1.0.1, https://github.com/bulik/ldsc) to obtain SNP-based heritability and pairwise genetic correlation across 63 biomarkers, 57 clinical phenotypes, and 30 molecular clusters of the CNSR-III population (*N* = 7695). GWAS summary statistics were cleaned and prepared as input. East Asian LD scores of high-quality common SNPs present in the HapMap 3 reference panel (MHC region excluded) were used as reference SNPs and weighted SNPs in the regression.^60,61^

### Partitioned heritability and functional enrichment for clusters

To assess the enrichment of heritability in functional annotations and MAF bins, a stratified LD score regression was performed to partition heritability into multiple functional categories. We applied heritability enrichment analyses using linkage disequilibrium score regression (LDSC) for 30 clusters and 63 biomarkers. We used the publicly available partitioned LD scores for predefined annotations based on the Roadmap Epigenomics Project provided by the LDSC authors (https://data.broadinstitute.org/alkescluster/LDSCORE). We generated the East Asian LD score reference for each annotation using the 1000 Genomes Project Phase 3 (v5). Hierarchical clustering was performed on the matrix of enrichment significance for the 30 clusters and 63 biomarkers. We only used highly significant enrichments (*P* < 0.05).^62^

### GWAS colocalization analysis

To identify shared causal variants among phenotypic traits and molecular clusters, we performed colocalization on pairwise GWAS summary statistics of 63 biomarkers and 30 clusters. We applied the function ‘coloc.abf’ from the ‘coloc’ packages in R, which assume that there is one causal signal in each input variant list. The prior probabilities were set as defaults *p*1 = 1 × 10^−4^; *p*2 = 1 × 10^−4^; *p*12 = 1 × 10^−5^. We considered SNPs to be significant with *P* < 1 × 10^−5^ in the GWAS statistics of 30 clusters as the leading SNPs. The input SNP lists were chosen as all variants scored by the GWAS within the flanking region of 1 Mb around the leading SNPs. We filtered the colocalization results with posterior probability of the fourth hypothesis where PP.H4 > 0.5 and *P* < 1 × 10^−5^ in both GWAS statistics of the pairwise traits.^63,64^

### Transcriptome-wide association study

We applied TWAS to infer genes whose expression may be altered by significant SNPs identified in the GWAS analysis. The gene expression imputation model was pre-trained using UTMOST (https://github.com/Joker-Jerome/UTMOST) with 44 tissues in the GTEx v6p data.^65^ We separately performed single-tissue association tests on the GWAS summary statistics of 63 biomarkers and 30 molecular clusters using pre-trained weights and covariance matrices for 44 tissues. Significant gene-level associations were established as *P* < 1.0 × 10^−7^ (0.05/17,291/30) for clusters and *P* < 5.0 × 10^−8^ (0.05/17,291/63) for biomarkers. Genes with absolute z-scores >4 were reported in this study. To determine the extent to which GWAS signals directly affect gene expression, conditional analyses were performed for genome-wide TWAS signals using FUSION.

### Fine-mapping of causal gene sets

To fine-map the causal genes associated with traits, we ran FOCUS (https://github.com/bogdanlab/focus) to perform a Bayesian test on the TWAS statistics.^66^ The GWAS summary statistics of 63 biomarkers and 30 clusters were used as inputs. The weight database integrated from FUSION and PrediXcan for FOCUS was downloaded.^67^ The LD scores were selected as the East Asian LD from 1000 Genomes Phase 3. For the 30 clusters, the *P*-value threshold was set to 1 × 10^−5^ to filter the input GWAS statistics. For 63 biomarkers, the default *P*-value threshold (5 × 10^−8^) was used. Causal genes with PIP > 0.5 are reported in this work.

### Prediction models for clinical outcomes across subpopulations

We applied a multilayer perceptron (MLP) composed of an input layer, output layer, and three hidden layers with 128 neurons as a binary classifier to predict clinical outcomes, including stroke recurrence at 1 year and poor functional outcome at 3 months. The top 500 SNPs associated with clinical outcomes and 63 biomarkers were selected to develop the polybiomarker prediction models. The hyperparameter optimization of the model was performed by a grid search with 3-fold cross-validation. With the optimal hyperparameters, the model was trained using cross-validation, wherein samples were subjected to a 10-fold split, in which 90% of the samples were used for training and 10% of the samples were used for evaluation. The model AUC from each cross-validation split was averaged to estimate overall classifier performance. The probability of stroke recurrence and poor functional outcome for each individual was generated and visualized on the UMAP.

### Statistical analysis

The chi-square test and Fisher’s exact test was used to analyze the associations of categorical variables and one-way analysis of variance for the association with continuous outcomes. Tukey’s multiple comparison test was used for the post-hoc analysis of variance (ANOVA). Survival analysis was performed using the Kaplan–Meier method with the log-rank test to examine statistically significant differences between clusters. Cox proportional hazards regression was used to calculate HRs and 95% CIs for each cluster with reference to all other clusters. We applied the Cox proportional hazards model to analyze the effects of biomarkers (e.g., continuous covariates) on clinical outcomes. Adjusted odds ratios (ORs) and 95% CIs were calculated using logistic regression. All models were adjusted for age, sex, alcohol consumption, smoking, history of stroke, dyslipidemia, diabetes mellitus, hypertension, and coronary heart disease. Statistical significance was defined as a two-sided *P*-value < 0.05.

## Supplementary information

Supplementary Information

Supplementary Table 16

## Data Availability

For whole-genome sequencing datasets, access was managed by the CNSR-III Data Access Committee. The variant databases are available following the regulation of the Human Genetic Resources Administration of China (HGRAC). The CNSR-III datasets are available at http://cnsr3.ncrcnd.org.cn. Any additional information required to reanalyze the data reported in this paper is available from the corresponding author upon reasonable request.
