# Supplementary material for: Biomarker and genomic analyses reveal molecular signatures of non-cardioembolic ischemic stroke
Source: Signal Transduct Target Ther. 2023 May 30;8:222. doi: 10.1038/s41392-023-01465-w (PMC10227023; doi:10.1038/s41392-023-01465-w)
Supplement: Supplementary file 1 — Supplementary Information [file 41392_2023_1465_MOESM1_ESM.docx]

Supplementary Information for

Biomarker and genomic analyses reveal molecular signatures of non-cardioembolic ischemic stroke

Lingling Ding, MD^1,2,3^; Yu Liu, PhD^2^; Xia Meng, MD^2^; Yong Jiang, PhD^2,3^; Jinxi Lin, MD^2^; Si Cheng, MD^2^; Zhe Xu, PhD^2^; Xingquan Zhao, MD^1,2,3^; Hao Li, MD^2^;Yongjun Wang, MD ^1,2,3,4,5,6^; Zixiao Li, MD ^1,2,3,7,8,*^

^1^Department of Neurology, Beijing Tiantan Hospital, Capital Medical University, Beijing, 100070, China

^2^China National Clinical Research Center for Neurological Diseases, Beijing, 100070, China

^3^Research Unit of Artificial Intelligence in Cerebrovascular Disease, Chinese Academy of Medical Sciences, Beijing, 100070, China

^4^Center for Excellence in Brain Science and Intelligence Technology, Chinese Academy of Sciences, Shanghai, 200031, China.

^5^Advanced Innovation Center for Human Brain Protection, Capital Medical University, Beijing, 100070, China.

^6^Clinical Center for Precision Medicine in Stroke, Capital Medical University, Beijing, 100070, China.

^7^Chinese Institute for Brain Research, Beijing, China

^8^Beijing Engineering Research Center of Digital Healthcare for Neurological Diseases, Beijing, 100070, China

^*^Correspondence to: lizixiao2008@hotmail.com

**This PDF file includes:**

Supplementary Figures (Figure S1-S4)

Supplementary Tables (Table S1- S15)

**Other Supplementary Materials for this manuscript include the following:**

Supplementary Table 16. Transcriptome-wide association study of clusters in multiple tissues.

**Supplementary Figure 1.** Dimensionality reduction for visualizing clusters and biomarkers. UMAP projection colored by clusters (left panel) and biomarkers for (**a**) inflammation, (**b**) renal function, and (**c**) liver function.

Abbreviations: hs-CRP, high-sensitivity C-reactive protein; IL-1Ra, Interleukin-1 receptor antagonist; IL-6, interleukin-6; UMA, urine microalbumin; ALT, alanine aminotransferase; AST, aspartate aminotransferase; IBIL, indirect bilirubin; DBIL, direct bilirubin.

**Supplementary Figure 2.** Dimensionality reduction for visualizing clusters and biomarkers. UMAP projection colored by clusters (left panel) and biomarkers for (**a**) lipid metabolism, and (**b**) homocysteine metabolism.

Abbreviations: Apo-AI, apolipoprotein AI; Apo-AII, apolipoprotein AII; Apo-E, apolipoprotein-E; Apo-B, apolipoprotein-B; MMA, methylmalonic aciduria; HCY, homocysteinemia.

**Supplementary Figure 3.** Dimensionality reduction for visualizing clusters and biomarkers. UMAP projection colored by clusters (left panel) and biomarkers for (**a**) coagulation function, (**b**) blood constituents, (**c**) gut-microbial metabolites, and (**d**) glucose metabolism.

Abbreviations: INR, international normalized ratio; MCV, mean corpuscular volume; RDWCV, coefficient of variation of RBC distribution width; TMAO, trimethylamine-N-oxide; FPG, fasting plasma glucose.

**Supplementary Figure 4.**Transcriptome-wide association study of clusters in multiple tissues.

(**a**) Transcriptome-wide association study of clusters in multiple tissues. (**b**) Chromosome 1 regional association plot. (**c**) Chromosome 2 regional association plot. (**d**) Chromosome 3 regional association plot. The top panel in each plot shows all the genes in the locus. The marginally TWAS-associated genes are highlighted in blue, while the jointly significant genes are in green. The bottom panel shows a Manhattan plot of the GWAS data before (gray) and after (blue) conditioning on the predicted expression of the green genes. The x-axis denotes genome coordinates, while the y-axis denotes association P-values in GWAS.

**Supplementary Table 1. Clinical phenotypes of patients included and excluded in the current analysis.**

|  | **Total (N = 15,166)** | | | **Included (n = 7,695)** | | | **Excluded (n = 7,471)** | | |  |
| --- | --- | --- | --- | --- | --- | --- | --- | --- | --- | --- |
| **Phenotypes** | **Missing(n)** | **Mean (SD)** | **Median (Q1,Q3)** | **Missing(n)** | **Mean (SD)** | **Median (Q1,Q3)** | **Missing(n)** | **Mean (SD)** | **Median (Q1,Q3)** | **P value^1^** |
| **Age (year)** | 0 | 62.23 (11.3) | 63  (54,70) | 0 | 61.44 (10.94) | 62  (54,69) | 0 | 63.05 (11.61) | 63  (55,71) | <0.001 |
| **DBP (mmHg)** | 0 | 87.35 (13.09) | 86  (79,95) | 0 | 88.06 (13.12) | 87.5 (80,96) | 0 | 86.63 (13.02) | 85  (78.5,95) | <0.001 |
| **SBP (mmHg)** | 0 | 150.08 (22.07) | 148 (135,163.5) | 0 | 151.35 (22.01) | 150 (136,165) | 0 | 148.76 (22.06) | 146.5 (133.25,161.5) | <0.001 |
| **BMI (kg/m^2^)** | 0 | 24.71 (3.34) | 24.49 (22.60,26.56) | 0 | 24.77 (3.30) | 24.49 (22.66,26.63) | 0 | 24.65 (3.38) | 24.47 (22.53,26.44) | 0.018 |
|  | **Missing(n)** | **Event**  **(n)** | **Ratio**  **(%)** | **Missing(n)** | **Event**  **(n)** | **Ratio**  **(%)** | **Missing(n)** | **Event**  **(n)** | **Ratio**  **(%)** | **P value^1^** |
| **Sex, Female** | 0 | 10,364 | 68.30% | 0 | 5,413 | 70.30% | 0 | 4,951 | 66.30% | <0.001 |
| **Drinking** | 0 | 2,126 | 14.00% | 0 | 1,210 | 15.70% | 0 | 916 | 12.30% | <0.001 |
| **Smoking** | 0 | 4,752 | 31.30% | 0 | 2,648 | 34.40% | 0 | 2,104 | 28.20% | <0.001 |
| **History of stroke** | 0 | 3,355 | 22.10% | 0 | 1,759 | 22.90% | 0 | 1,596 | 21.40% | 0.028 |
| **Family history of diabetes** | 827 | 13,339 | 88.00% | 457 | 6,704 | 87.10% | 370 | 6,635 | 88.80% | 0.002 |
| **Family history of hypertension** | 839 | 11,102 | 73.20% | 464 | 5,551 | 72.10% | 375 | 5,551 | 74.30% | 0.003 |
| **Family history of hyperlipidemia** | 1676 | 13,136 | 86.60% | 836 | 6,682 | 86.80% | 840 | 6,454 | 86.40% | 0.431 |
| **Family history of stroke** | 806 | 12,347 | 81.40% | 435 | 6,205 | 80.60% | 371 | 6,142 | 82.20% | 0.013 |
| **Hypertension** | 0 | 4,066 | 26.80% | 0 | 1,915 | 24.90% | 0 | 2,151 | 28.80% | <0.001 |
| **Lipid metabolism disorder** | 0 | 9,613 | 63.40% | 0 | 4,729 | 61.50% | 0 | 4,884 | 65.40% | <0.001 |
| **Liver disease** | 0 | 14,240 | 93.90% | 0 | 7,187 | 93.40% | 0 | 7,053 | 94.40% | 0.011 |
| **PAD** | 0 | 14,324 | 94.40% | 0 | 7,243 | 94.10% | 0 | 7,081 | 94.80% | 0.085 |
| **CHD** | 0 | 12,854 | 84.80% | 0 | 6,696 | 87.00% | 0 | 6,158 | 82.40% | <0.001 |
| **Myocardial infarction** | 0 | 236 | 1.60% | 0 | 114 | 1.50% | 0 | 122 | 1.60% | 0.491 |
| **Impaired glucose tolerance** | 4365 | 376 | 2.50% | 2422 | 199 | 2.60% | 1943 | 177 | 2.40% | 0.42 |
| **IFG** | 4365 | 120 | 0.80% | 2422 | 68 | 0.90% | 1943 | 52 | 0.70% | 0.225 |
| **T1DM** | 485 | 31 | 0.20% | 262 | 13 | 0.20% | 223 | 18 | 0.20% | 0.423 |
| **T2DM** | 485 | 4,338 | 28.60% | 262 | 2,411 | 31.30% | 223 | 1,927 | 25.80% | <0.001 |
| **Respiratory disease** | 0 | 14,326 | 94.50% | 0 | 7,354 | 95.60% | 0 | 6,972 | 93.30% | <0.001 |
| **Pulmonary infection** | 14329 | 824 | 5.40% | 7354 | 335 | 4.40% | 6975 | 489 | 6.50% | <0.001 |
| **Urinary tract infection** | 0 | 231 | 1.50% | 0 | 102 | 1.30% | 0 | 129 | 1.70% | 0.051 |
| **Renal insufficiency** | 0 | 195 | 1.30% | 0 | 86 | 1.10% | 0 | 109 | 1.50% | 0.073 |
| **DVT** | 0 | 15,083 | 99.50% | 0 | 7,655 | 99.50% | 0 | 7,428 | 99.40% | 0.723 |
| **Epilepsy** | 0 | 15,094 | 99.50% | 0 | 7,657 | 99.50% | 0 | 7,437 | 99.50% | 0.819 |
| **Bleeding** | 0 | 14,951 | 98.60% | 0 | 7,602 | 98.80% | 0 | 7,349 | 98.40% | 0.032 |
| **sECAS** | 2154 | 596 | 3.90% | 1030 | 295 | 3.80% | 1124 | 301 | 4.00% | 0.564 |
| **sICAS** | 2154 | 3,813 | 25.10% | 1030 | 1,874 | 24.40% | 1124 | 1,939 | 26.00% | 0.024 |
| **TOAST-LAA** | 0 | 3,856 | 25.40% | 0 | 2,135 | 27.70% | 0 | 1,721 | 23.00% | <0.001 |
| **TOAST-CE** | 0 | 917 | 6.00% | 0 | 0 | 0.00% | 0 | 917 | 12.30% | <0.001 |
| **TOAST-SAO** | 0 | 3,165 | 20.90% | 0 | 1,996 | 25.90% | 0 | 1,169 | 15.60% | <0.001 |
| **TOAST-OE** | 0 | 182 | 1.20% | 0 | 0 | 0.00% | 0 | 182 | 2.40% | <0.001 |
| **TOAST-UE** | 0 | 7,046 | 46.50% | 0 | 3,564 | 46.30% | 0 | 3,482 | 46.60% | 0.732 |
| **Anti-platelet therapy** | 113 | 440 | 2.90% | 60 | 128 | 1.70% | 53 | 312 | 4.20% | <0.001 |
| **Aspirin** | 553 | 13,200 | 87.00% | 188 | 6,792 | 88.30% | 365 | 6,408 | 85.80% | <0.001 |
| **Clopidogrel** | 553 | 9,044 | 59.60% | 188 | 4,776 | 62.10% | 365 | 4,268 | 57.10% | <0.001 |
| **Dipyridamole** | 553 | 39 | 0.30% | 188 | 17 | 0.20% | 365 | 22 | 0.30% | 0.463 |
| **Cilostazol** | 553 | 95 | 0.60% | 188 | 52 | 0.70% | 365 | 43 | 0.60% | 0.497 |
| **Hypoglycemic treatment** | 113 | 11,261 | 74.30% | 60 | 5,536 | 71.90% | 53 | 5,725 | 76.60% | <0.001 |
| **Antihypertensive treatment** | 113 | 8,053 | 53.10% | 60 | 4,045 | 52.60% | 53 | 4,008 | 53.60% | 0.188 |
| **Lipid-lowering drugs** | 113 | 547 | 3.60% | 60 | 193 | 2.50% | 53 | 354 | 4.70% | <0.001 |
| **Hemorrhagic stroke in hospital** | 78 | 15,057 | 99.30% | 18 | 7,667 | 99.60% | 60 | 7,390 | 98.90% | <0.001 |
| **Ischemic stroke in hospital** | 98 | 14,439 | 95.20% | 31 | 7,335 | 95.30% | 67 | 7,104 | 95.10% | 0.525 |
| **Hemorrhagic transformation** | 95 | 14,878 | 98.10% | 25 | 7,602 | 98.80% | 70 | 7,276 | 97.40% | <0.001 |
| **Systemic embolic events in hospital** | 117 | 15,018 | 99.00% | 34 | 7,654 | 99.50% | 83 | 7364 | 98.60% | <0.001 |
| **Death in hospital** | 0 | 35 | 0.20% | 0 | 14 | 0.20% | 0 | 21 | 0.30% | 0.27 |
| **Hemorrhagic stroke at 3 months** | 0 | 69 | 0.50% | 0 | 26 | 0.30% | 0 | 43 | 0.60% | 0.04 |
| **Combined vascular event at 3 months** | 0 | 977 | 6.40% | 0 | 479 | 6.20% | 0 | 498 | 6.70% | 0.283 |
| **Death at 3 months** | 0 | 218 | 1.40% | 0 | 87 | 1.10% | 0 | 131 | 1.80% | 0.002 |
| **Ischemic stroke at 3 months** | 0 | 888 | 5.90% | 0 | 448 | 5.80% | 0 | 440 | 5.90% | 0.887 |
| **mRS at 3 mongths** | 182 | 2,075 | 13.70% | 81 | 1,000 | 13.00% | 101 | 1,075 | 14.40% | 0.013 |
| **Stroke at 3 months** | 0 | 944 | 6.20% | 0 | 469 | 6.10% | 0 | 475 | 6.40% | 0.524 |
| **Combined vascular event at 6 months** | 0 | 1,242 | 8.20% | 0 | 617 | 8.00% | 0 | 625 | 8.40% | 0.453 |
| **Death at 6 months** | 0 | 325 | 2.10% | 0 | 133 | 1.70% | 0 | 192 | 2.60% | <0.001 |
| **mRS at 6 months** | 198 | 1,888 | 12.40% | 94 | 907 | 11.80% | 104 | 981 | 13.10% | 0.013 |
| **Stroke at 6 months** | 0 | 1,185 | 7.80% | 0 | 596 | 7.70% | 0 | 589 | 7.90% | 0.774 |
| **Combined vascular event at 1 year** | 0 | 1,557 | 10.30% | 0 | 762 | 9.90% | 0 | 795 | 10.60% | 0.141 |
| **Death at 1 year** | 0 | 498 | 3.30% | 0 | 196 | 2.50% | 0 | 302 | 4.00% | <0.001 |
| **Stroke at 1 year** | 0 | 1,473 | 9.70% | 0 | 732 | 9.50% | 0 | 741 | 9.90% | 0.414 |
| **mRS at 1 year** | 369 | 1,981 | 13.10% | 190 | 920 | 12.00% | 179 | 1,061 | 14.20% | <0.001 |

^1^Compared between the included and excluded patients. A value of P < 0.05 was considered to be statistically significant.

Abbreviations: N, number; SD, standard deviation; Q1, first quartile; Q1, fourth quartile; SBP, systolic blood pressure; DBP, diastolic blood pressure; BMI, body mass index; IFG, impaired fasting glucose; T1DM, type 1 diabetes mellitus; T2DM, type 2 diabetes mellitus; CHD, coronary heart disease; PAD, peripheral arterial disease; DVT, deep venous thrombosis; sICAS, symptomatic intracranial atherosclerotic stenosis; sECAS, symptomatic extracranial atherosclerotic stenosis; TOAST, Trial of Org 10 172 in Acute Stroke Treatment; LAA, large-artery atherosclerosis; SAO, small-artery occlusion; CE, cardioembolism; OE, other determined etiology; UE, undetermined etiology; mRS, modified Rankin scale.

**Supplementary Table 2. Clinical phenotypes and biomarkers.**

| **Phenotypes** | **Group** | **Subgroup** | **Description** |
| --- | --- | --- | --- |
| **Clinical phenotypes** | | | |
| **Age** | Clinical phenotype | Risk factor | Basic Information: Age(years old) |
| **Gender** | Clinical phenotype | Risk factor | Basic Information: Gender; 1-male; 2-female |
| **SBP** | Clinical phenotype | Risk factor | Physical examination: Blood pressure at admission: **Systolic blood pressure (mmHg)** |
| **DBP** | Clinical phenotype | Risk factor | Physical examination: Blood pressure at admission**: Diastolic blood pressure (mmHg)** |
| **BMI** | Clinical phenotype | Risk factor | Physical examination: **Body mass index (kg/m^2^)** |
| **Smoking** | Clinical phenotype | Risk factor | History: Smoking; 0-No,1-Yes |
| **Drinking** | Clinical phenotype | Risk factor | History: Drinking; 0-No,1-Yes |
| **History of stroke** | Clinical phenotype | Risk factor | History: Stroke History; 0-No; 1-Yes |
| **Family history of hypertension** | Clinical phenotype | Family history | Family History: Hypertension; 1-No; 2-Yes |
| **Family history of hyperlipidemia** | Clinical phenotype | Family history | Family History: Hyperlipidemia; 1-No; 2-Yes |
| **Family history of diabetes** | Clinical phenotype | Family history | Family History: Diabetes; 1-No; 2-Yes |
| **Family history of stroke** | Clinical phenotype | Family history | Family History: Stroke; 1-No; 2-Yes |
| **Hypertension** | Clinical phenotype | Comorbidity | Final diagnosis: Hypertension; 1-No; 2-Yes |
| **Impaired glucose tolerance** | Clinical phenotype | Comorbidity | Final diagnosis: pre-diabetes; 1-impaired glucose tolerance; 0-No; 1-Yes |
| **IFG** | Clinical phenotype | Comorbidity | Final diagnosis: pre-diabetes; 2-**impaired fasting glucose(IFG)**; 0-No; 1-Yes |
| **T1DM** | Clinical phenotype | Comorbidity | Final diagnosis: diabetes classification; 1- **type 1 diabetes mellitus**; 0-No; 1-Yes |
| **T2DM** | Clinical phenotype | Comorbidity | Final diagnosis: diabetes classification; 2- **type 2 diabetes mellitus**; 0-No; 1-Yes |
| **Lipid metabolism disorder** | Clinical phenotype | Comorbidity | Final diagnosis: lipid metabolism disorder; 1-No; 2-Yes |
| **CHD** | Clinical phenotype | Comorbidity | Final diagnosis: **coronary heart disease**; 1-No; 2-Yes |
| **Myocardial infarction** | Clinical phenotype | Comorbidity | Final diagnosis: coronary heart disease; 1-myocardial infarction; 0-No; 1-Yes |
| **PAD** | Clinical phenotype | Comorbidity | Final diagnosis: **peripheral arterial disease**; 1-No; 2-Yes |
| **Liver disease** | Clinical phenotype | Comorbidity | Final diagnosis: liver disease; 1-No; 2-Yes |
| **Renal insufficiency** | Clinical phenotype | Comorbidity | Final diagnosis: 2-renal insufficiency; 0-No; 1-Yes |
| **Respiratory disease** | Clinical phenotype | Complication | Final diagnosis: Respiratory disease; 1-No; 2-Yes |
| **Urinary tract infection** | Clinical phenotype | Complication | Final diagnosis:; 1-urinary tract infection; 0-No; 1-Yes |
| **Pulmonary infection** | Clinical phenotype | Complication | Final diagnosis: pulmonary infection; 1-Yes; 2-No |
| **DVT** | Clinical phenotype | Complication | Final diagnosis: **Deep venous thrombosis**; 1-No; 2-Yes |
| **Bleeding** | Clinical phenotype | Complication | Final diagnosis: bleeding; 1-No; 2-Yes |
| **Epilepsy** | Clinical phenotype | Complication | Final diagnosis: Epilepsy; 1-No; 2-Yes |
| **sICAS** | Clinical phenotype | Etiology | **Symptomatic intracranial atherosclerotic stenosis criminal artery**:0-No;1-Yes |
| **sECAS** | Clinical phenotype | Etiology | **Symptomatic extracranial atherosclerotic stenosis criminal artery**:0-No;1-Yes |
| **TOAST** | Clinical phenotype | Etiology | **Trial of Org 10 172 in Acute Stroke Treatment (TOAST)**:1-Large artery atherosclerosis; 2-Cardic embolism; 3-Small vascular occlusion; 4-Other determination; 5-Undetermined |
| **Anti-platelet therapy** | Clinical phenotype | Medications | Inpatient Therapy: Anti-platelet therapy; 1-No; 2-Yes |
| **Lipid-lowering drugs** | Clinical phenotype | Medications | Inpatient Therapy: Lipid-lowering drugs; 1-No; 2-Yes |
| **Hypoglycemic treatment** | Clinical phenotype | Medications | Inpatient Therapy: Hypoglycemic treatment; 1-No; 2-Yes |
| **Antihypertensive treatment** | Clinical phenotype | Medications | Inpatient Therapy: Antihypertensive treatment; 1-No; 2-Yes |
| **Aspirin** | Clinical phenotype | Medications | Inpatient Therapy: Anti-platelet drugs category; 1-aspirin; 0-No; 1-Yes |
| **Clopidogrel** | Clinical phenotype | Medications | Inpatient Therapy: Anti-platelet drugs category; 2-clopidogrel; 0-No; 1-Yes |
| **Dipyridamole** | Clinical phenotype | Medications | Inpatient Therapy: Anti-platelet drugs category; 3-dipyridamole; 0-No; 1-Yes |
| **Cilostazol** | Clinical phenotype | Medications | Inpatient Therapy: Anti-platelet drugs category; 4-cilostazol; 0-No; 1-Yes |
| **Ischemic stroke in hospital** | Clinical phenotype | Clinical outcome | Inpatient Event: Ischemic Stroke; 1-No; 2-Yes; |
| **Hemorrhagic stroke in hospital** | Clinical phenotype | Clinical outcome | Inpatient Event: Hemorrhagic stroke; 1-No; 2-Yes |
| **Hemorrhagic transformation** | Clinical phenotype | Clinical outcome | Inpatient Event: Hemorrhagic transformation after infarction; 1-No; 2-Yes |
| **Systemic embolic events in hospital** | Clinical phenotype | Clinical outcome | Inpatient Event: systemic embolic events; 1-no; 2-yes |
| **Death in hospital** | Clinical phenotype | Clinical outcome | Discharge status: 1-death; 2-survival |
| **Ischemic stroke at 3 months** | Clinical phenotype | Clinical outcome | Follow-up events at 3 months: recurrence of ischemic stroke: 0-No; 1-Yes |
| **Hemorrhagic stroke at 3 months** | Clinical phenotype | Clinical outcome | Follow-up events at 3 months: recurrence of hemorrhage stroke: 0-No; 1-Yes |
| **Stroke at 1 year** | Clinical phenotype | Clinical outcome | Follow-up events at 12 months: Recurrence of stroke: 0-No; 1-Yes |
| **Stroke at 6 months** | Clinical phenotype | Clinical outcome | Follow-up events at 6 months: Recurrence of stroke: 0-No; 1-Yes |
| **Stroke at 3 months** | Clinical phenotype | Clinical outcome | Follow-up events at 3 months: Recurrence of stroke: 0-No; 1-Yes |
| **Combined vascular event at 1 year** | Clinical phenotype | Clinical outcome | Follow-up events at 12 months: Occurrence of combined vascular event: 0-No; 1-Yes |
| **Combined vascular event at 6 months** | Clinical phenotype | Clinical outcome | Follow-up events at 6 months: Occurrence of combined vascular event: 0-No; 1-Yes |
| **Combined vascular event at 3 months** | Clinical phenotype | Clinical outcome | Follow-up events at 3 months: Occurrence of combined vascular event: 0-No; 1-Yes |
| **Death at 1 year** | Clinical phenotype | Clinical outcome | Follow-up events at 12 months: Whether the patient died: 0-survival;1-death |
| **Death at 6 months** | Clinical phenotype | Clinical outcome | Follow-up events at 6 months: Whether the patient died: 0-survival;1-death |
| **Death at 3 months** | Clinical phenotype | Clinical outcome | Follow-up events at 3 months: Whether the patient died: 0-survival;1-death |
| **mRS at 3 mongths** | Clinical phenotype | Clinical outcome | Follow-up events at 3 months: Modified Rankin Scale (mRS) score at follow-up; 0-(0-2) points;1-(3-6) points |
| **mRS at 6 mongths** | Clinical phenotype | Clinical outcome | Follow-up events at 6 months: Modified Rankin Scale (mRS) score at follow-up; 0-(0-2) points;1-(3-6) points |
| **mRS at 1 year** | Clinical phenotype | Clinical outcome | Follow-up events at 12 months: Modified Rankin Scale (mRS) score at follow-up; 0-(0-2) points;1-(3-6) points |
| **Biomarkers** | | | |
| **Angiopoietin-Like 3 (mg/ml)** | Biomarker | Lipid metabolism | Angiopoietin-Like 3 |
| **PCSK9 (ng/ml)** | Biomarker | Lipid metabolism | Proprotein convertase subtilisin/Kexin type 9 |
| **Lp-PLA2-Activity (nmol/min/ml)** | Biomarker | Lipid metabolism | Lipoprotein- associated phospolipase A2 activity |
| **LDL (mmol/L)** | Biomarker | Lipid metabolism | Low density lipoprotein |
| **Lp(a) (mg/dL)** | Biomarker | Lipid metabolism | Lipoprotein (a) |
| **Adiponectin (mg/ml)** | Biomarker | Lipid metabolism | Adiponectin |
| **HDL (mmol/L)** | Biomarker | Lipid metabolism | High density lipoprotein |
| **LDL-R (ng/ml)** | Biomarker | Lipid metabolism | Low density lipoprotein-Receptor |
| **Triglyceride (mmol/L)** | Biomarker | Lipid metabolism | Triglyceride |
| **Apolipoprotein-E (g/L)** | Biomarker | Lipid metabolism | Apolipoprotein-E |
| **Apolipoprotein-AI (g/L)** | Biomarker | Lipid metabolism | Apolipoprotein-AI |
| **Apolipoprotein-AII (g/L)** | Biomarker | Lipid metabolism | Apolipoprotein-AII |
| **Apolipoprotein-B (g/L)** | Biomarker | Lipid metabolism | Apolipoprotein-B |
| **MCV (fL)** | Biomarker | Blood constituents | Mean corpuscular volume |
| **Basophil (×10^9/L)** | Biomarker | Blood constituents | Basophil |
| **Eosinophil (×10^9/L)** | Biomarker | Blood constituents | Eosinophil |
| **Platelet (×10^9/L)** | Biomarker | Blood constituents | Platelet |
| **RDWCV (%)** | Biomarker | Blood constituents | Red blood cell distribution width |
| **MPV (fL)** | Biomarker | Blood constituents | Mean platelet volume |
| **Hemoglobin (g/L)** | Biomarker | Blood constituents | Hemoglobin |
| **MCHC (g/L)** | Biomarker | Blood constituents | Mean corpuscular hemoglobin concentration |
| **APTT (s)** | Biomarker | Coagulation function | Activated Partial Thromboplastin Time |
| **INR** | Biomarker | Coagulation function | International normalized ratio |
| **Prothrombin time (s)** | Biomarker | Coagulation function | Prothrombin time |
| **Fibrinogen (mg/dl)** | Biomarker | Coagulation function | Fibrinogen |
| **D-dimer (μg/ml)** | Biomarker | Coagulation function | D-dimer |
| **Thrombin time (s)** | Biomarker | Coagulation function | Thrombin time |
| **Chlorine (mmol/L)** | Biomarker | Electrolyte | Chlorine |
| **Sodium (mmol/L)** | Biomarker | Electrolyte | Sodium |
| **Potassium (mmol/L)** | Biomarker | Electrolyte | Potassium |
| **FPG (mmol/L)** | Biomarker | Glucose metabolism | Fsting plasma glucose |
| **TMAVA (μmmol/)** | Biomarker | Gut-microbial metabolites | N,N,N-trimethyl-5-aminovaleric acid |
| **TMAO (μmmol/)** | Biomarker | Gut-microbial metabolites | Trimethylamine-N-oxide |
| **TML (μmmol/)** | Biomarker | Gut-microbial metabolites | Trimethyllysine |
| **Carnitine (μmmol/)** | Biomarker | Gut-microbial metabolites | Carnitine |
| **Butyrobetaine (μmmol/)** | Biomarker | Gut-microbial metabolites | Butyrobetaine |
| **Betaine (μmmol/)** | Biomarker | Gut-microbial metabolites | Betaine |
| **Choline (μmmol/)** | Biomarker | Gut-microbial metabolites | Choline |
| **MMA (nmol/L)** | Biomarker | Homocysteine metabolism | Methylmalonic aciduria |
| **HCY (μmol/L)** | Biomarker | Homocysteine metabolism | Homocysteinemia |
| **Folic acid (nmol/L)** | Biomarker | Homocysteine metabolism | Folic acid |
| **Vitamin B12 (pmol/L)** | Biomarker | Homocysteine metabolism | Vitamin B12 |
| **Monocyte (×10^9/L)** | Biomarker | Inflammation | Monocyte |
| **Neutrophil (×10^9/L)** | Biomarker | Inflammation | Neutrophil |
| **Lymphocyte (×10^9/L)** | Biomarker | Inflammation | Lymphocyte |
| **IL-6 (pg/ml)** | Biomarker | Inflammation | Interleukin-6 |
| **hs-CRP (mg/L)** | Biomarker | Inflammation | High-sensitivity C-reactive protein |
| **IL-1Ra (pg/ml)** | Biomarker | Inflammation | Interleukin-1 receptor antagonist |
| **YKL-40 (pg/ml)** | Biomarker | Inflammation | Chitinase-3-like Protein 1 |
| **MCP-1 (pg/ml)** | Biomarker | Inflammation | Monocyte chemoattractant protein-1 |
| **IL-6R (pg/ml)** | Biomarker | Inflammation | Interleukin-6 receptor |
| **Alkaline phosphatase (U/L)** | Biomarker | Liver function | Alkaline phosphatase |
| **Globulin (g/L)** | Biomarker | Liver function | Globulin |
| **GGT (U/L)** | Biomarker | Liver function | γ-Glutamyl transpeptidase |
| **ALT (U/L)** | Biomarker | Liver function | Alanine aminotransferase |
| **AST (U/L)** | Biomarker | Liver function | Aspertate Aminotransferase |
| **DBIL (μmol/L)** | Biomarker | Liver function | Direct bilirubin |
| **IBIL (μmol/L)** | Biomarker | Liver function | Indirect bilirubin |
| **Albumin (g/L)** | Biomarker | Liver function | Albumin |
| **Uric acid (μmol/L)** | Biomarker | Renal function | Uric acid |
| **Cystatin C (mg/L)** | Biomarker | Renal function | Cystatin C |
| **Creatinine (μmol/L)** | Biomarker | Renal function | Creatinine |
| **UMA (mg/L)** | Biomarker | Renal function | Urine microalbumin |

**Supplementary Table 3. Excluded biomarkers.**

| **Phenotypes** | **Group** | **Excluded** | **Description** |
| --- | --- | --- | --- |
| **RBC (×10^9/L)** | Biomarker | With ≥25% missing | Red blood cell |
| **Calcium (mmol/L)** | Biomarker | With ≥25% missing | Calcium |
| **HbA1c (%)** | Biomarker | With ≥25% missing | Hemoglobin A1c |
| **LDH (U/L)** | Biomarker | With ≥25% missing | Lactate dehydrogenase |
| **Total bile acid (μmol/L)** | Biomarker | With ≥25% missing | Total bile acid |
| **CO2 combining power (mmol/L)** | Biomarker | With ≥25% missing | CO2 combining power |
| **PLCR(%)** | Biomarker | With ≥25% missing | Platelet large cell ratio |
| **Hematocrit (%)** | Biomarker | Correlation coefficient of >0.7 | Hematocrit |
| **Total protein (g/L)** | Biomarker | Correlation coefficient of >0.7 | Total protein |
| **MCH (pg)** | Biomarker | Correlation coefficient of >0.7 | Mean corpuscular hemoglobin |
| **WBC (×10^9/L)** | Biomarker | Correlation coefficient of >0.7 | White blood cell |
| **Thrombocytocrit (%)** | Biomarker | Correlation coefficient of >0.7 | Thrombocytocrit |
| **TBIL (μmol/L)** | Biomarker | Correlation coefficient of >0.7 | Total bilirubin |
| **Lp-PLA2-Mass (ng/ml)** | Biomarker | Correlation coefficient of >0.7 | Lipoprotein- associated phospolipase A2 mass |
| **UACR (mg/mmol)** | Biomarker | Correlation coefficient of >0.7 | Urea albumin creatinine ratio |
| **Cholesterol (mmol/L)** | Biomarker | Correlation coefficient of >0.7 | Cholesterol |
| **Apolipoprotein-CIII (g/L)** | Biomarker | Correlation coefficient of >0.7 | Apolipoprotein-CIII |
| **Apolipoprotein-CII (g/L)** | Biomarker | Correlation coefficient of >0.7 | Apolipoprotein-CII |

**Supplementary Table 4. Clinical phenotypes across clusters.**

|  | **Cluster 1** | | **Cluster 2** | | **Cluster 3** | | **Cluster 4** | | **Cluster 5** | |
| --- | --- | --- | --- | --- | --- | --- | --- | --- | --- | --- |
|  | **Mean (SD)** | **Median (IQR)** | **Mean (SD)** | **Median**  **(IQR)** | **Mean (SD)** | **Median (IQR)** | **Mean (SD)** | **Median (IQR)** | **Mean (SD)** | **Median (IQR)** |
| **Age** | 64.6 (11.3) | 64  (58,73) | 62.7 (10.9) | 63  (54, 69) | 59.1 (12.0) | 59.5  (50,67) | 67.1  (11.2) | 67  (60,76) | 59.3 (10.7) | 60  (51,67) |
| **DBP (mmHg)** | 84.3 (11.9) | 82  (79,93) | 86.6 (13.1) | 84  (77.3, 92) | 88.3 (12.9) | 88.3  (80,97) | 85.2 (13.2) | 84.5 (76.9,91.6) | 93.4 (16.6) | 90 (80,102.5） |
| **SBP (mmHg)** | 153.5 (23.5) | 150 (141,166.5) | 147.3 (20.5) | 146  (133.4, 160.9) | 150.1 (21.3) | 150 (136.1,162.4) | 149.4 (21.8) | 147.5 (135.5,163.8) | 164.6 (28.1) | 160.5 (142.5,180) |
| **BMI (Kg/m2)** | 23.9 (3.1) | 23.9 (21.7,26.0) | 24.1 (2.8) | 24.219  (22.2, 26.0) | 25.1 (3.3) | 24.7 (22.9,27.4) | 24.2 (3.4) | 24.2 (22,26.1） | 25.0 (2.8) | 24.3 (23.4,26) |
|  | **Event**  **(n)** | **Ratio**  **(%)** | **Event**  **(n)** | **Ratio**  **(%)** | **Event**  **(n)** | **Ratio**  **(%)** | **Event**  **(n)** | **Ratio**  **(%)** | **Event**  **(n)** | **Ratio**  **(%)** |
| **Gender, Female** | 12 | 22.6% | 20 | 28.6% | 45 | 23.2% | 83 | 26.9% | 17 | 34.7% |
| **Drinking** | 5 | 9.4% | 10 | 14.3% | 28 | 14.4% | 44 | 14.3% | 3 | 6.1% |
| **Smoking** | 17 | 32.1% | 19 | 27.1% | 75 | 38.7% | 95 | 30.8% | 16 | 32.7% |
| **History of stroke** | 21 | 39.6% | 17 | 24.3% | 48 | 24.7% | 87 | 28.2% | 15 | 30.6% |
| **Family history of diabetes** | 3 | 5.7% | 2 | 2.9% | 9 | 4.6% | 23 | 7.5% | 8 | 16.3% |
| **Family history of hypertension** | 8 | 15.1% | 15 | 21.4% | 40 | 20.6% | 56 | 18.2% | 15 | 30.6% |
| **Family history of hyperlipidemia** | 1 | 1.9% | 2 | 2.9% | 4 | 2.1% | 7 | 2.3% | 2 | 4.1% |
| **Family history of stroke** | 0 | 0.0% | 10 | 14.3% | 28 | 14.4% | 23 | 7.5% | 9 | 18.4% |
| **Hypertension** | 42 | 79.2% | 46 | 65.7% | 148 | 76.3% | 223 | 72.4% | 44 | 89.8% |
| **Lipid metabolism disorder** | 10 | 18.9% | 26 | 37.1% | 75 | 38.7% | 101 | 32.8% | 26 | 53.1% |
| **Liver disease** | 5 | 9.4% | 5 | 7.1% | 17 | 8.8% | 15 | 4.9% | 0 | 0.0% |
| **PAD** | 2 | 3.8% | 4 | 5.7% | 11 | 5.7% | 17 | 5.5% | 7 | 14.3% |
| **CHD** | 7 | 13.2% | 10 | 14.3% | 28 | 14.4% | 50 | 16.2% | 6 | 12.2% |
| **Myocardial infarction** | 2 | 3.8% | 2 | 2.9% | 3 | 1.5% | 10 | 3.2% | 0 | 0.0% |
| **Impaired glucose tolerance** | 3 | 5.7% | 0 | 0.0% | 8 | 4.1% | 3 | 1.0% | 0 | 0.0% |
| **IFG** | 1 | 1.9% | 0 | 0.0% | 2 | 1.0% | 5 | 1.6% | 0 | 0.0% |
| **T1DM** | 0 | 0.0% | 0 | 0.0% | 1 | 0.5% | 0 | 0.0% | 0 | 0.0% |
| **T2DM** | 17 | 32.1% | 17 | 24.3% | 54 | 27.8% | 99 | 32.1% | 40 | 81.6% |
| **Renal insufficiency** | 3 | 5.7% | 1 | 1.4% | 3 | 1.5% | 1 | 0.3% | 10 | 20.4% |
| **Urinary tract infection** | 4 | 7.5% | 3 | 4.3% | 5 | 2.6% | 10 | 3.2% | 3 | 6.1% |
| **Respiratory disease** | 20 | 37.7% | 7 | 10.0% | 19 | 9.8% | 58 | 18.8% | 3 | 6.1% |
| **Pulmonary infection** | 20 | 37.7% | 7 | 10.0% | 19 | 9.8% | 58 | 18.8% | 3 | 6.1% |
| **DVT** | 0 | 0.0% | 4 | 5.7% | 3 | 1.5% | 2 | 0.6% | 0 | 0.0% |
| **Epilepsy** | 1 | 1.9% | 1 | 1.4% | 0 | 0.0% | 4 | 1.3% | 0 | 0.0% |
| **Bleeding** | 2 | 3.8% | 1 | 1.4% | 6 | 3.1% | 7 | 2.3% | 1 | 2.0% |
| **sECAS** | 1 | 1.9% | 5 | 7.1% | 10 | 5.2% | 25 | 8.1% | 3 | 6.1% |
| **sICAS** | 18 | 34.0% | 25 | 35.7% | 56 | 28.9% | 106 | 34.4% | 13 | 26.5% |
| **TOAST-Large artery atherosclerosis** | 19 | 35.8% | 30 | 42.9% | 68 | 35.1% | 119 | 38.6% | 14 | 28.6% |
| **TOAST-Small vascular occlusion** | 9 | 17.0% | 8 | 11.4% | 39 | 20.1% | 51 | 16.6% | 11 | 22.4% |
| **TOAST-Undetermined causes** | 25 | 47.2% | 32 | 45.7% | 87 | 44.8% | 138 | 44.8% | 24 | 49.0% |
| **Anti-platelet therapy** | 50 | 94.3% | 64 | 91.4% | 186 | 95.9% | 294 | 95.5% | 47 | 95.9% |
| **Aspirin** | 43 | 81.1% | 54 | 77.1% | 155 | 79.9% | 258 | 83.8% | 44 | 89.8% |
| **Clopidogrel** | 30 | 56.6% | 31 | 44.3% | 127 | 65.5% | 173 | 56.2% | 24 | 49.0% |
| **Dipyridamole** | 0 | 0.0% | 0 | 0.0% | 1 | 0.5% | 1 | 0.3% | 0 | 0.0% |
| **Cilostazol** | 0 | 0.0% | 1 | 1.4% | 1 | 0.5% | 5 | 1.6% | 0 | 0.0% |
| **Hypoglycemic treatment** | 14 | 26.4% | 17 | 24.3% | 50 | 25.8% | 90 | 29.2% | 39 | 79.6% |
| **Antihypertensive treatment** | 22 | 41.5% | 27 | 38.6% | 80 | 41.2% | 129 | 41.9% | 35 | 71.4% |
| **Lipid-lowering drugs** | 49 | 92.5% | 65 | 92.9% | 189 | 97.4% | 296 | 96.1% | 46 | 93.9% |
| **Hemorrhagic stroke in hospital** | 0 | 0.0% | 0 | 0.0% | 0 | 0.0% | 0 | 0.0% | 0 | 0.0% |
| **Ischemic stroke in hospital** | 5 | 9.4% | 5 | 7.1% | 13 | 6.7% | 23 | 7.5% | 2 | 4.1% |
| **Hemorrhagic transformation** | 2 | 3.8% | 2 | 2.9% | 4 | 2.1% | 12 | 3.9% | 0 | 0.0% |
| **Systemic embolic events in hospital** | 0 | 0.0% | 0 | 0.0% | 2 | 1.0% | 0 | 0.0% | 0 | 0.0% |
| **Death in hospital** | 1 | 1.9% | 1 | 1.4% | 2 | 1.0% | 2 | 0.6% | 0 | 0.0% |
| **Hemorrhagic stroke at 3 months** | 0 | 0.0% | 0 | 0.0% | 1 | 0.5% | 1 | 0.3% | 0 | 0.0% |
| **Combined vascular event at 3 months** | 7 | 13.2% | 7 | 10.0% | 22 | 11.3% | 26 | 8.4% | 3 | 6.1% |
| **Death at 3 months** | 5 | 9.4% | 6 | 8.6% | 7 | 3.6% | 10 | 3.2% | 3 | 6.1% |
| **Ischemic stroke at 3 months** | 7 | 13.2% | 7 | 10.0% | 21 | 10.8% | 25 | 8.1% | 3 | 6.1% |
| **mRS at 3 mongths** | 20 | 37.7% | 25 | 35.7% | 41 | 21.1% | 101 | 32.8% | 17 | 34.7% |
| **Stroke at 3 months** | 7 | 13.2% | 7 | 10.0% | 21 | 10.8% | 26 | 8.4% | 3 | 6.1% |
| **Combined vascular event at 6 months** | 11 | 20.8% | 9 | 12.9% | 29 | 14.9% | 35 | 11.4% | 5 | 10.2% |
| **Death at 6 months** | 8 | 15.1% | 8 | 11.4% | 10 | 5.2% | 18 | 5.8% | 6 | 12.2% |
| **mRS at 6 mongths** | 17 | 32.1% | 21 | 30.0% | 39 | 20.1% | 90 | 29.2% | 12 | 24.5% |
| **Stroke at 6 months** | 11 | 20.8% | 9 | 12.9% | 27 | 13.9% | 33 | 10.7% | 5 | 10.2% |
| **Combined vascular event at 1 year** | 12 | 22.6% | 11 | 15.7% | 33 | 17.0% | 42 | 13.6% | 8 | 16.3% |
| **Death at 1 year** | 8 | 15.1% | 8 | 11.4% | 11 | 5.7% | 25 | 8.1% | 9 | 18.4% |
| **Stroke at 1 year** | 12 | 22.6% | 11 | 15.7% | 30 | 15.5% | 40 | 13.0% | 7 | 14.3% |
| **mRS at 1 year** | 19 | 35.8% | 18 | 25.7% | 44 | 22.7% | 100 | 32.5% | 12 | 24.5% |

|  | **Cluster 6** | | **Cluster 7** | | **Cluster 8** | | **Cluster 9** | | **Cluster 10** | |
| --- | --- | --- | --- | --- | --- | --- | --- | --- | --- | --- |
|  | **Mean (SD)** | **Median (IQR)** | **Mean (SD)** | **Median**  **(IQR)** | **Mean (SD)** | **Median (IQR)** | **Mean (SD)** | **Median (IQR)** | **Mean (SD)** | **Median (IQR)** |
| **Age** | 64.4 (10.7) | 63  (58,72.5) | 67.5  (11.9) | 68  (61,76) | 60.4  (11.8,62) | 62  (53,69) | 57.7  (11.7) | 58  (50,66.5) | 61.3  (10.7) | 62  (55,68.5) |
| **DBP (mmHg)** | 85.5 (13.1) | 85.75  (76.4,92.5) | 87.1  (15.3) | 85  (79,97.5) | 88.6  (14.3) | 88.5 (79,95) | 89  (13.6) | 89  (80,98) | 88.8  (13.8) | 87.5  (79.8,98) |
| **SBP (mmHg)** | 149.8 (24.7) | 147  (132.5,163.8) | 156.3  (27.2) | 158.5  (139.5,174) | 151  (22.6) | 147 (137.5,165.5) | 147.5  (22.8) | 146.5  (130.5,160.5) | 154.4  (23) | 152  (140,169) |
| **BMI (Kg/m2)** | 25  (3.8) | 24.6  (22.5,27) | 24.7  (3.4) | 24.2  (22.5,26.5) | 25(3.2) | 24.6 (22.5,26.8) | 25.2  (3.3) | 25.2  (23.3,27.1) | 24.7  (2.9) | 24.4  (23.1,26.1) |
|  | **Event**  **(n)** | **Ratio**  **(%)** | **Event**  **(n)** | **Ratio**  **(%)** | **Event**  **(n)** | **Ratio**  **(%)** | **Event**  **(n)** | **Ratio**  **(%)** | **Event**  **(n)** | **Ratio**  **(%)** |
| **Gender, Female** | 16 | 18.2% | 32 | 20.9% | 22 | 27.2% | 14 | 7.7% | 40 | 19.0% |
| **Drinking** | 20 | 22.7% | 16 | 10.5% | 15 | 18.5% | 39 | 21.3% | 34 | 16.1% |
| **Smoking** | 33 | 37.5% | 43 | 28.1% | 31 | 38.3% | 96 | 52.5% | 91 | 43.1% |
| **History of stroke** | 21 | 23.9% | 50 | 32.7% | 15 | 18.5% | 47 | 25.7% | 63 | 29.9% |
| **Family history of diabetes** | 7 | 8.0% | 14 | 9.2% | 1 | 1.2% | 13 | 7.1% | 16 | 7.6% |
| **Family history of hypertension** | 14 | 15.9% | 42 | 27.5% | 23 | 28.4% | 41 | 22.4% | 58 | 27.5% |
| **Family history of hyperlipidemia** | 2 | 2.3% | 3 | 2.0% | 2 | 2.5% | 2 | 1.1% | 8 | 3.8% |
| **Family history of stroke** | 9 | 10.2% | 24 | 15.7% | 11 | 13.6% | 33 | 18.0% | 38 | 18.0% |
| **Hypertension** | 67 | 76.1% | 133 | 86.9% | 58 | 71.6% | 128 | 69.9% | 163 | 77.3% |
| **Lipid metabolism disorder** | 35 | 39.8% | 58 | 37.9% | 29 | 35.8% | 56 | 30.6% | 89 | 42.2% |
| **Liver disease** | 10 | 11.4% | 12 | 7.8% | 1 | 1.2% | 7 | 3.8% | 15 | 7.1% |
| **PAD** | 5 | 5.7% | 11 | 7.2% | 0 | 0.0% | 9 | 4.9% | 16 | 7.6% |
| **CHD** | 8 | 9.1% | 38 | 24.8% | 10 | 12.3% | 12 | 6.6% | 31 | 14.7% |
| **Myocardial infarction** | 3 | 3.4% | 5 | 3.3% | 0 | 0.0% | 2 | 1.1% | 7 | 3.3% |
| **Impaired glucose tolerance** | 3 | 3.4% | 5 | 3.3% | 2 | 2.5% | 5 | 2.7% | 3 | 1.4% |
| **IFG** | 1 | 1.1% | 2 | 1.3% | 1 | 1.2% | 0 | 0.0% | 5 | 2.4% |
| **T1DM** | 0 | 0.0% | 1 | 0.7% | 0 | 0.0% | 0 | 0.0% | 1 | 0.5% |
| **T2DM** | 39 | 44.3% | 54 | 35.3% | 20 | 24.7% | 22 | 12.0% | 54 | 25.6% |
| **Renal insufficiency** | 1 | 1.1% | 23 | 15.0% | 0 | 0.0% | 0 | 0.0% | 0 | 0.0% |
| **Urinary tract infection** | 1 | 1.1% | 1 | 0.7% | 1 | 1.2% | 2 | 1.1% | 2 | 0.9% |
| **Respiratory disease** | 3 | 3.4% | 12 | 7.8% | 3 | 3.7% | 3 | 1.6% | 5 | 2.4% |
| **Pulmonary infection** | 3 | 3.4% | 12 | 7.8% | 3 | 3.7% | 3 | 1.6% | 5 | 2.4% |
| **DVT** | 1 | 1.1% | 3 | 2.0% | 0 | 0.0% | 2 | 1.1% | 1 | 0.5% |
| **Epilepsy** | 0 | 0.0% | 0 | 0.0% | 0 | 0.0% | 0 | 0.0% | 2 | 0.9% |
| **Bleeding** | 2 | 2.3% | 3 | 2.0% | 0 | 0.0% | 1 | 0.5% | 0 | 0.0% |
| **sECAS** | 3 | 3.4% | 5 | 3.3% | 0 | 0.0% | 9 | 4.9% | 5 | 2.4% |
| **sICAS** | 19 | 21.6% | 40 | 26.1% | 22 | 27.2% | 45 | 24.6% | 44 | 20.9% |
| **TOAST-Large artery atherosclerosis** | 21 | 23.9% | 51 | 33.3% | 24 | 29.6% | 52 | 28.4% | 48 | 22.7% |
| **TOAST-Small vascular occlusion** | 32 | 36.4% | 26 | 17.0% | 13 | 16.0% | 50 | 27.3% | 47 | 22.3% |
| **TOAST-Undetermined causes** | 35 | 39.8% | 76 | 49.7% | 44 | 54.3% | 81 | 44.3% | 116 | 55.0% |
| **Anti-platelet therapy** | 86 | 97.7% | 149 | 97.4% | 79 | 97.5% | 179 | 97.8% | 208 | 98.6% |
| **Aspirin** | 78 | 88.6% | 133 | 86.9% | 71 | 87.7% | 164 | 89.6% | 193 | 91.5% |
| **Clopidogrel** | 54 | 61.4% | 85 | 55.6% | 59 | 72.8% | 98 | 53.6% | 127 | 60.2% |
| **Dipyridamole** | 0 | 0.0% | 0 | 0.0% | 0 | 0.0% | 0 | 0.0% | 0 | 0.0% |
| **Cilostazol** | 2 | 2.3% | 0 | 0.0% | 2 | 2.5% | 0 | 0.0% | 1 | 0.5% |
| **Hypoglycemic treatment** | 33 | 37.5% | 51 | 33.3% | 14 | 17.3% | 19 | 10.4% | 46 | 21.8% |
| **Antihypertensive treatment** | 38 | 43.2% | 92 | 60.1% | 43 | 53.1% | 80 | 43.7% | 100 | 47.4% |
| **Lipid-lowering drugs** | 86 | 97.7% | 146 | 95.4% | 78 | 96.3% | 177 | 96.7% | 205 | 97.2% |
| **Hemorrhagic stroke in hospital** | 0 | 0.0% | 1 | 0.7% | 0 | 0.0% | 1 | 0.5% | 0 | 0.0% |
| **Ischemic stroke in hospital** | 1 | 1.1% | 11 | 7.2% | 2 | 2.5% | 10 | 5.5% | 6 | 2.8% |
| **Hemorrhagic transformation** | 0 | 0.0% | 2 | 1.3% | 0 | 0.0% | 1 | 0.5% | 0 | 0.0% |
| **Systemic embolic events in hospital** | 0 | 0.0% | 0 | 0.0% | 0 | 0.0% | 0 | 0.0% | 0 | 0.0% |
| **Death in hospital** | 1 | 1.1% | 1 | 0.7% | 0 | 0.0% | 0 | 0.0% | 0 | 0.0% |
| **Hemorrhagic stroke at 3 months** | 0 | 0.0% | 1 | 0.7% | 1 | 1.2% | 3 | 1.6% | 0 | 0.0% |
| **Combined vascular event at 3 months** | 3 | 3.4% | 18 | 11.8% | 3 | 3.7% | 13 | 7.1% | 8 | 3.8% |
| **Death at 3 months** | 1 | 1.1% | 5 | 3.3% | 1 | 1.2% | 3 | 1.6% | 2 | 0.9% |
| **Ischemic stroke at 3 months** | 2 | 2.3% | 16 | 10.5% | 2 | 2.5% | 11 | 6.0% | 7 | 3.3% |
| **mRS at 3 mongths** | 8 | 9.1% | 35 | 22.9% | 7 | 8.6% | 20 | 10.9% | 16 | 7.6% |
| **Stroke at 3 months** | 2 | 2.3% | 17 | 11.1% | 3 | 3.7% | 13 | 7.1% | 7 | 3.3% |
| **Combined vascular event at 6 months** | 5 | 5.7% | 23 | 15.0% | 7 | 8.6% | 17 | 9.3% | 10 | 4.7% |
| **Death at 6 months** | 2 | 2.3% | 8 | 5.2% | 1 | 1.2% | 4 | 2.2% | 2 | 0.9% |
| **mRS at 6 mongths** | 10 | 11.4% | 43 | 28.1% | 7 | 8.6% | 17 | 9.3% | 13 | 6.2% |
| **Stroke at 6 months** | 4 | 4.5% | 20 | 13.1% | 7 | 8.6% | 17 | 9.3% | 9 | 4.3% |
| **Combined vascular event at 1 year** | 9 | 10.2% | 30 | 19.6% | 7 | 8.6% | 21 | 11.5% | 16 | 7.6% |
| **Death at 1 year** | 2 | 2.3% | 14 | 9.2% | 1 | 1.2% | 4 | 2.2% | 2 | 0.9% |
| **Stroke at 1 year** | 8 | 9.1% | 26 | 17.0% | 7 | 8.6% | 21 | 11.5% | 15 | 7.1% |
| **mRS at 1 year** | 7 | 8.0% | 42 | 27.5% | 8 | 9.9% | 17 | 9.3% | 16 | 7.6% |

|  | **Cluster 11** | | **Cluster 12** | | **Cluster 13** | | **Cluster 14** | | **Cluster 15** | |
| --- | --- | --- | --- | --- | --- | --- | --- | --- | --- | --- |
|  | **Mean (SD)** | **Median (IQR)** | **Mean (SD)** | **Median**  **(IQR)** | **Mean (SD)** | **Median (IQR)** | **Mean (SD)** | **Median (IQR)** | **Mean (SD)** | **Median (IQR)** |
| **Age** | 63.8  (11.2) | 64  (57,72) | 61.9  (9.4) | 62  (55,68) | 63.6  (10.6) | 64  (57,71) | 61.6  (9.8) | 60  (55,69) | 59.6  (12.4) | 58  (50,69) |
| **DBP (mmHg)** | 86.4  (12.6) | 85.5  (78,94) | 87.3  (12.4) | 87  (79,95) | 86.7  (13.2) | 86.5  (77.5,96.5) | 86.8  (12.4) | 86  (79,94) | 87.2  (13.6) | 84  (79,94) |
| **SBP (mmHg)** | 149.6  (21.7) | 147.5  (135,164) | 151.8  (21) | 150  (137,165) | 150.3  (21.9) | 149.5  (134.5,166) | 147.0  (20.3) | 145  (134.5,157) | 152.5  (22.3) | 151.5  (138,165) |
| **BMI (Kg/m2)** | 24.5  (3.3) | 24.3  (22.5,26.1) | 24.6  (3.2) | 24.2  (22.5,26.4) | 23.8  (3.2) | 23.7  (21.8,25.5) | 24.5  (3.7) | 24.1  (22.2,26) | 24.5  (3.5) | 24  (21.9,27.1) |
|  | **Event**  **(n)** | **Ratio**  **(%)** | **Event**  **(n)** | **Ratio**  **(%)** | **Event**  **(n)** | **Ratio**  **(%)** | **Event**  **(n)** | **Ratio**  **(%)** | **Event**  **(n)** | **Ratio**  **(%)** |
| **Gender, Female** | 112 | 16.5% | 543 | 54.8% | 205 | 36.0% | 31 | 24.8% | 57 | 56.4% |
| **Drinking** | 97 | 14.3% | 86 | 8.7% | 90 | 15.8% | 18 | 14.4% | 7 | 6.9% |
| **Smoking** | 248 | 36.6% | 207 | 20.9% | 186 | 32.7% | 40 | 32.0% | 18 | 17.8% |
| **History of stroke** | 178 | 26.3% | 215 | 21.7% | 123 | 21.6% | 26 | 20.8% | 18 | 17.8% |
| **Family history of diabetes** | 45 | 6.6% | 59 | 6.0% | 27 | 4.7% | 5 | 4.0% | 4 | 4.0% |
| **Family history of hypertension** | 158 | 23.3% | 225 | 22.7% | 98 | 17.2% | 17 | 13.6% | 19 | 18.8% |
| **Family history of hyperlipidemia** | 15 | 2.2% | 25 | 2.5% | 14 | 2.5% | 3 | 2.4% | 1 | 1.0% |
| **Family history of stroke** | 98 | 14.5% | 132 | 13.3% | 76 | 13.4% | 8 | 6.4% | 19 | 18.8% |
| **Hypertension** | 499 | 73.7% | 752 | 75.9% | 389 | 68.4% | 94 | 75.2% | 74 | 73.3% |
| **Lipid metabolism disorder** | 171 | 25.3% | 342 | 34.5% | 151 | 26.5% | 65 | 52.0% | 28 | 27.7% |
| **Liver disease** | 38 | 5.6% | 47 | 4.7% | 27 | 4.7% | 6 | 4.8% | 7 | 6.9% |
| **PAD** | 40 | 5.9% | 64 | 6.5% | 23 | 4.0% | 16 | 12.8% | 5 | 5.0% |
| **CHD** | 78 | 11.5% | 103 | 10.4% | 60 | 10.5% | 8 | 6.4% | 9 | 8.9% |
| **Myocardial infarction** | 7 | 1.0% | 6 | 0.6% | 7 | 1.2% | 1 | 0.8% | 0 | 0.0% |
| **Impaired glucose tolerance** | 19 | 2.8% | 19 | 1.9% | 9 | 1.6% | 2 | 1.6% | 5 | 5.0% |
| **IFG** | 5 | 0.7% | 9 | 0.9% | 3 | 0.5% | 0 | 0.0% | 1 | 1.0% |
| **T1DM** | 0 | 0.0% | 2 | 0.2% | 1 | 0.2% | 0 | 0.0% | 0 | 0.0% |
| **T2DM** | 181 | 26.7% | 254 | 25.6% | 100 | 17.6% | 38 | 30.4% | 22 | 21.8% |
| **Renal insufficiency** | 4 | 0.6% | 1 | 0.1% | 1 | 0.2% | 1 | 0.8% | 0 | 0.0% |
| **Urinary tract infection** | 4 | 0.6% | 10 | 1.0% | 9 | 1.6% | 1 | 0.8% | 2 | 2.0% |
| **Respiratory disease** | 22 | 3.2% | 25 | 2.5% | 19 | 3.3% | 7 | 5.6% | 7 | 6.9% |
| **Pulmonary infection** | 22 | 3.2% | 25 | 2.5% | 19 | 3.3% | 7 | 5.6% | 7 | 6.9% |
| **DVT** | 6 | 0.9% | 2 | 0.2% | 1 | 0.2% | 0 | 0.0% | 1 | 1.0% |
| **Epilepsy** | 2 | 0.3% | 5 | 0.5% | 4 | 0.7% | 1 | 0.8% | 1 | 1.0% |
| **Bleeding** | 9 | 1.3% | 13 | 1.3% | 8 | 1.4% | 1 | 0.8% | 2 | 2.0% |
| **sECAS** | 20 | 3.0% | 34 | 3.4% | 20 | 3.5% | 6 | 4.8% | 1 | 1.0% |
| **sICAS** | 149 | 22.0% | 240 | 24.2% | 119 | 20.9% | 23 | 18.4% | 33 | 32.7% |
| **TOAST-Large artery atherosclerosis** | 163 | 24.1% | 272 | 27.4% | 134 | 23.6% | 26 | 20.8% | 35 | 34.7% |
| **TOAST-Small vascular occlusion** | 209 | 30.9% | 278 | 28.1% | 165 | 29.0% | 43 | 34.4% | 18 | 17.8% |
| **TOAST-Undetermined causes** | 305 | 45.1% | 441 | 44.5% | 270 | 47.5% | 56 | 44.8% | 48 | 47.5% |
| **Anti-platelet therapy** | 651 | 96.2% | 974 | 98.3% | 560 | 98.4% | 121 | 96.8% | 96 | 95.0% |
| **Aspirin** | 590 | 87.1% | 885 | 89.3% | 509 | 89.5% | 105 | 84.0% | 86 | 85.1% |
| **Clopidogrel** | 436 | 64.4% | 625 | 63.1% | 353 | 62.0% | 77 | 61.6% | 61 | 60.4% |
| **Dipyridamole** | 1 | 0.1% | 0 | 0.0% | 3 | 0.5% | 0 | 0.0% | 0 | 0.0% |
| **Cilostazol** | 1 | 0.1% | 6 | 0.6% | 3 | 0.5% | 1 | 0.8% | 1 | 1.0% |
| **Hypoglycemic treatment** | 161 | 23.8% | 212 | 21.4% | 77 | 13.5% | 38 | 30.4% | 19 | 18.8% |
| **Antihypertensive treatment** | 307 | 45.3% | 442 | 44.6% | 237 | 41.7% | 67 | 53.6% | 46 | 45.5% |
| **Lipid-lowering drugs** | 641 | 94.7% | 965 | 97.4% | 560 | 98.4% | 120 | 96.0% | 94 | 93.1% |
| **Hemorrhagic stroke in hospital** | 1 | 0.1% | 1 | 0.1% | 1 | 0.2% | 0 | 0.0% | 0 | 0.0% |
| **Ischemic stroke in hospital** | 24 | 3.5% | 37 | 3.7% | 19 | 3.3% | 4 | 3.2% | 1 | 1.0% |
| **Hemorrhagic transformation** | 4 | 0.6% | 11 | 1.1% | 3 | 0.5% | 0 | 0.0% | 2 | 2.0% |
| **Systemic embolic events in hospital** | 0 | 0.0% | 1 | 0.1% | 0 | 0.0% | 0 | 0.0% | 0 | 0.0% |
| **Death in hospital** | 0 | 0.0% | 0 | 0.0% | 0 | 0.0% | 0 | 0.0% | 0 | 0.0% |
| **Hemorrhagic stroke at 3 months** | 2 | 0.3% | 2 | 0.2% | 3 | 0.5% | 0 | 0.0% | 0 | 0.0% |
| **Combined vascular event at 3 months** | 39 | 5.8% | 56 | 5.7% | 30 | 5.3% | 5 | 4.0% | 3 | 3.0% |
| **Death at 3 months** | 5 | 0.7% | 5 | 0.5% | 5 | 0.9% | 0 | 0.0% | 0 | 0.0% |
| **Ischemic stroke at 3 months** | 38 | 5.6% | 53 | 5.3% | 27 | 4.7% | 5 | 4.0% | 3 | 3.0% |
| **mRS at 3 mongths** | 70 | 10.3% | 109 | 11.0% | 60 | 10.5% | 13 | 10.4% | 15 | 14.9% |
| **Stroke at 3 months** | 39 | 5.8% | 55 | 5.5% | 30 | 5.3% | 5 | 4.0% | 3 | 3.0% |
| **Combined vascular event at 6 months** | 47 | 6.9% | 69 | 7.0% | 39 | 6.9% | 7 | 5.6% | 4 | 4.0% |
| **Death at 6 months** | 6 | 0.9% | 9 | 0.9% | 6 | 1.1% | 0 | 0.0% | 0 | 0.0% |
| **mRS at 6 mongths** | 66 | 9.7% | 99 | 10.0% | 56 | 9.8% | 14 | 11.2% | 14 | 13.9% |
| **Stroke at 6 months** | 46 | 6.8% | 68 | 6.9% | 38 | 6.7% | 7 | 5.6% | 4 | 4.0% |
| **Combined vascular event at 1 year** | 50 | 7.4% | 84 | 8.5% | 55 | 9.7% | 10 | 8.0% | 6 | 5.9% |
| **Death at 1 year** | 12 | 1.8% | 14 | 1.4% | 11 | 1.9% | 1 | 0.8% | 0 | 0.0% |
| **Stroke at 1 year** | 49 | 7.2% | 83 | 8.4% | 53 | 9.3% | 10 | 8.0% | 6 | 5.9% |
| **mRS at 1 year** | 74 | 10.9% | 93 | 9.4% | 56 | 9.8% | 17 | 13.6% | 15 | 14.9% |

|  | **Cluster 16** | | **Cluster 17** | | **Cluster 18** | | **Cluster 19** | | **Cluster 20** | |
| --- | --- | --- | --- | --- | --- | --- | --- | --- | --- | --- |
|  | **Mean (SD)** | **Median (IQR)** | **Mean (SD)** | **Median**  **(IQR)** | **Mean (SD)** | **Median (IQR)** | **Mean (SD)** | **Median (IQR)** | **Mean (SD)** | **Median (IQR)** |
| **Age** | 61.9  (9.9) | 62.5  (54,68) | 57.3  (11.4) | 57  (49,65.3) | 57.3  (10.2) | 56.5  (52,62) | 56.6  (9.7) | 55  (49.3,63) | 62.1  (11.0) | 61  (55,68) |
| **DBP (mmHg)** | 88.7  (11.7) | 89.8  (80,97.6) | 91.4  (14.4) | 92.5  (81.8,100) | 89.8  (14.6) | 89  (79.5) | 91.1  (13.1) | 90  (83,100) | 85.2  (11.7) | 86  (77.5,93.5) |
| **SBP (mmHg)** | 154.9  (23.0) | 150.8  (139.8,172.6) | 152.2  (23.2) | 151  (138.9,163.1) | 151.3  (21.3) | 150  (134.5,165) | 151.5  (19.6) | 150  (139.3,165) | 147.2  (21.1) | 145  (132,160) |
| **BMI (Kg/m2)** | 24.7  (2.6) | 24.6  (22.7,26.2) | 25.7  (3.7) | 25.4  (23.4,28) | 24.7  (3.2) | 24.6  (22.5,26.8) | 25.5  (3.1) | 25.1  (23.4,27.3) | 24.5  (3.6) | 24.2  (22,26.6) |
|  | **Event**  **(n)** | **Ratio**  **(%)** | **Event**  **(n)** | **Ratio**  **(%)** | **Event**  **(n)** | **Ratio**  **(%)** | **Event**  **(n)** | **Ratio**  **(%)** | **Event**  **(n)** | **Ratio**  **(%)** |
| **Gender, Female** | 50 | 39.1% | 48 | 37.5% | 15 | 9.5% | 79 | 44.4% | 30 | 33.7% |
| **Drinking** | 15 | 11.7% | 15 | 11.7% | 72 | 45.6% | 20 | 11.2% | 12 | 13.5% |
| **Smoking** | 39 | 30.5% | 37 | 28.9% | 90 | 57.0% | 47 | 26.4% | 32 | 36.0% |
| **History of stroke** | 22 | 17.2% | 20 | 15.6% | 18 | 11.4% | 39 | 21.9% | 21 | 23.6% |
| **Family history of diabetes** | 5 | 3.9% | 13 | 10.2% | 12 | 7.6% | 16 | 9.0% | 7 | 7.9% |
| **Family history of hypertension** | 33 | 25.8% | 27 | 21.1% | 29 | 18.4% | 40 | 22.5% | 22 | 24.7% |
| **Family history of hyperlipidemia** | 2 | 1.6% | 5 | 3.9% | 2 | 1.3% | 4 | 2.2% | 2 | 2.2% |
| **Family history of stroke** | 18 | 14.1% | 19 | 14.8% | 21 | 13.3% | 29 | 16.3% | 6 | 6.7% |
| **Hypertension** | 99 | 77.3% | 104 | 81.3% | 116 | 73.4% | 146 | 82.0% | 62 | 69.7% |
| **Lipid metabolism disorder** | 53 | 41.4% | 48 | 37.5% | 81 | 51.3% | 106 | 59.6% | 40 | 44.9% |
| **Liver disease** | 3 | 2.3% | 41 | 32.0% | 31 | 19.6% | 13 | 7.3% | 9 | 10.1% |
| **PAD** | 4 | 3.1% | 5 | 3.9% | 14 | 8.9% | 4 | 2.2% | 8 | 9.0% |
| **CHD** | 24 | 18.8% | 18 | 14.1% | 15 | 9.5% | 29 | 16.3% | 16 | 18.0% |
| **Myocardial infarction** | 3 | 2.3% | 1 | 0.8% | 0 | 0.0% | 3 | 1.7% | 1 | 1.1% |
| **Impaired glucose tolerance** | 4 | 3.1% | 4 | 3.1% | 7 | 4.4% | 5 | 2.8% | 1 | 1.1% |
| **IFG** | 2 | 1.6% | 0 | 0.0% | 1 | 0.6% | 2 | 1.1% | 0 | 0.0% |
| **T1DM** | 1 | 0.8% | 0 | 0.0% | 0 | 0.0% | 0 | 0.0% | 0 | 0.0% |
| **T2DM** | 31 | 24.2% | 55 | 43.0% | 52 | 32.9% | 94 | 52.8% | 23 | 25.8% |
| **Renal insufficiency** | 3 | 2.3% | 2 | 1.6% | 0 | 0.0% | 0 | 0.0% | 0 | 0.0% |
| **Urinary tract infection** | 1 | 0.8% | 3 | 2.3% | 2 | 1.3% | 0 | 0.0% | 2 | 2.2% |
| **Respiratory disease** | 6 | 4.7% | 4 | 3.1% | 10 | 6.3% | 5 | 2.8% | 6 | 6.7% |
| **Pulmonary infection** | 6 | 4.7% | 4 | 3.1% | 10 | 6.3% | 5 | 2.8% | 6 | 6.7% |
| **DVT** | 0 | 0.0% | 1 | 0.8% | 0 | 0.0% | 2 | 1.1% | 1 | 1.1% |
| **Epilepsy** | 1 | 0.8% | 1 | 0.8% | 0 | 0.0% | 0 | 0.0% | 1 | 1.1% |
| **Bleeding** | 0 | 0.0% | 5 | 3.9% | 1 | 0.6% | 1 | 0.6% | 0 | 0.0% |
| **sECAS** | 1 | 0.8% | 3 | 2.3% | 7 | 4.4% | 7 | 3.9% | 5 | 5.6% |
| **sICAS** | 20 | 15.6% | 27 | 21.1% | 45 | 28.5% | 46 | 25.8% | 27 | 30.3% |
| **TOAST-Large artery atherosclerosis** | 20 | 15.6% | 28 | 21.9% | 47 | 29.7% | 52 | 29.2% | 30 | 33.7% |
| **TOAST-Small vascular occlusion** | 29 | 22.7% | 35 | 27.3% | 42 | 26.6% | 56 | 31.5% | 17 | 19.1% |
| **TOAST-Undetermined causes** | 79 | 61.7% | 65 | 50.8% | 69 | 43.7% | 70 | 39.3% | 42 | 47.2% |
| **Anti-platelet therapy** | 127 | 99.2% | 123 | 96.1% | 152 | 96.2% | 177 | 99.4% | 87 | 97.8% |
| **Aspirin** | 121 | 94.5% | 110 | 85.9% | 137 | 86.7% | 162 | 91.0% | 83 | 93.3% |
| **Clopidogrel** | 50 | 39.1% | 81 | 63.3% | 97 | 61.4% | 105 | 59.0% | 45 | 50.6% |
| **Dipyridamole** | 2 | 1.6% | 0 | 0.0% | 0 | 0.0% | 2 | 1.1% | 0 | 0.0% |
| **Cilostazol** | 1 | 0.8% | 1 | 0.8% | 1 | 0.6% | 4 | 2.2% | 0 | 0.0% |
| **Hypoglycemic treatment** | 29 | 22.7% | 46 | 35.9% | 48 | 30.4% | 78 | 43.8% | 22 | 24.7% |
| **Antihypertensive treatment** | 72 | 56.3% | 68 | 53.1% | 85 | 53.8% | 88 | 49.4% | 33 | 37.1% |
| **Lipid-lowering drugs** | 122 | 95.3% | 117 | 91.4% | 155 | 98.1% | 177 | 99.4% | 83 | 93.3% |
| **Hemorrhagic stroke in hospital** | 0 | 0.0% | 1 | 0.8% | 0 | 0.0% | 0 | 0.0% | 0 | 0.0% |
| **Ischemic stroke in hospital** | 1 | 0.8% | 8 | 6.3% | 6 | 3.8% | 8 | 4.5% | 2 | 2.2% |
| **Hemorrhagic transformation** | 0 | 0.0% | 2 | 1.6% | 3 | 1.9% | 1 | 0.6% | 1 | 1.1% |
| **Systemic embolic events in hospital** | 0 | 0.0% | 0 | 0.0% | 1 | 0.6% | 0 | 0.0% | 1 | 1.1% |
| **Death in hospital** | 0 | 0.0% | 1 | 0.8% | 0 | 0.0% | 1 | 0.6% | 0 | 0.0% |
| **Hemorrhagic stroke at 3 months** | 0 | 0.0% | 2 | 1.6% | 1 | 0.6% | 0 | 0.0% | 0 | 0.0% |
| **Combined vascular event at 3 months** | 1 | 0.8% | 12 | 9.4% | 10 | 6.3% | 11 | 6.2% | 4 | 4.5% |
| **Death at 3 months** | 1 | 0.8% | 1 | 0.8% | 2 | 1.3% | 1 | 0.6% | 1 | 1.1% |
| **Ischemic stroke at 3 months** | 1 | 0.8% | 10 | 7.8% | 9 | 5.7% | 11 | 6.2% | 4 | 4.5% |
| **mRS at 3 mongths** | 17 | 13.3% | 24 | 18.8% | 16 | 10.1% | 11 | 6.2% | 12 | 13.5% |
| **Stroke at 3 months** | 1 | 0.8% | 12 | 9.4% | 10 | 6.3% | 11 | 6.2% | 4 | 4.5% |
| **Combined vascular event at 6 months** | 5 | 3.9% | 14 | 10.9% | 12 | 7.6% | 13 | 7.3% | 5 | 5.6% |
| **Death at 6 months** | 2 | 1.6% | 3 | 2.3% | 3 | 1.9% | 1 | 0.6% | 1 | 1.1% |
| **mRS at 6 mongths** | 19 | 14.8% | 22 | 17.2% | 14 | 8.9% | 7 | 3.9% | 11 | 12.4% |
| **Stroke at 6 months** | 4 | 3.1% | 14 | 10.9% | 12 | 7.6% | 12 | 6.7% | 4 | 4.5% |
| **Combined vascular event at 1 year** | 7 | 5.5% | 15 | 11.7% | 12 | 7.6% | 14 | 7.9% | 6 | 6.7% |
| **Death at 1 year** | 2 | 1.6% | 4 | 3.1% | 4 | 2.5% | 1 | 0.6% | 3 | 3.4% |
| **Stroke at 1 year** | 6 | 4.7% | 15 | 11.7% | 12 | 7.6% | 13 | 7.3% | 5 | 5.6% |
| **mRS at 1 year** | 11 | 8.6% | 22 | 17.2% | 15 | 9.5% | 7 | 3.9% | 13 | 14.6% |

|  | **Cluster 21** | | **Cluster 22** | | **Cluster 23** | | **Cluster 24** | | **Cluster 25** | |
| --- | --- | --- | --- | --- | --- | --- | --- | --- | --- | --- |
|  | **Mean (SD)** | **Median (IQR)** | **Mean (SD)** | **Median**  **(IQR)** | **Mean (SD)** | **Median (IQR)** | **Mean (SD)** | **Median (IQR)** | **Mean (SD)** | **Median (IQR)** |
| **Age** | 60.4  (11.4) | 61  (52,68.5) | 63.8  (10.0) | 64  (57.8,70.3) | 60.8  (11.7) | 61  (52,68) | 63.3  (10.2) | 63  (56,70.5) | 60.4  (8.9) | 60  (54,67) |
| **DBP (mmHg)** | 88.7  (13.7) | 88.5  (80,96) | 86.7  (13.2) | 83  (78,93.9) | 87.9  (12.4) | 87  (80,95) | 88.6  (12.3) | 87.5  (80,96.8) | 87.1  (12.1) | 87.5  (78.6,95) |
| **SBP (mmHg)** | 150.8  (21.9) | 150  (136.5,162.3) | 150.4  (22.2) | 147.3  (132.5,165.3) | 149.3  (20.4) | 146  (133.5,162.3) | 151.8  (22.0) | 150  (137.5,163.8) | 152.9  (20.4) | 149.5  (138,167) |
| **BMI (Kg/m2)** | 24.7  (2.9) | 24.5  (22.7,26.7) | 25.1  (3.6) | 24.8  (22.9,26.8) | 24.8  (3.1) | 24.5  (22.9,26.1) | 25  (3.5) | 24.5  (22.9,26.7) | 24.4  (3.4) | 24.2  (22.2,25.9) |
|  | **Event**  **(n)** | **Ratio**  **(%)** | **Event**  **(n)** | **Ratio**  **(%)** | **Event**  **(n)** | **Ratio**  **(%)** | **Event**  **(n)** | **Ratio**  **(%)** | **Event**  **(n)** | **Ratio**  **(%)** |
| **Gender, Female** | 48 | 35.6% | 83 | 31.4% | 72 | 20.1% | 185 | 22.4% | 47 | 41.2% |
| **Drinking** | 21 | 15.6% | 47 | 17.8% | 76 | 21.2% | 135 | 16.3% | 15 | 13.2% |
| **Smoking** | 42 | 31.1% | 90 | 34.1% | 172 | 47.9% | 314 | 38.0% | 39 | 34.2% |
| **History of stroke** | 22 | 16.3% | 61 | 23.1% | 96 | 26.7% | 188 | 22.7% | 26 | 22.8% |
| **Family history of diabetes** | 5 | 3.7% | 25 | 9.5% | 19 | 5.3% | 60 | 7.3% | 7 | 6.1% |
| **Family history of hypertension** | 20 | 14.8% | 60 | 22.7% | 65 | 18.1% | 174 | 21.0% | 17 | 14.9% |
| **Family history of hyperlipidemia** | 2 | 1.5% | 9 | 3.4% | 7 | 1.9% | 18 | 2.2% | 3 | 2.6% |
| **Family history of stroke** | 12 | 8.9% | 30 | 11.4% | 46 | 12.8% | 108 | 13.1% | 14 | 12.3% |
| **Hypertension** | 104 | 77.0% | 200 | 75.8% | 253 | 70.5% | 642 | 77.6% | 81 | 71.1% |
| **Lipid metabolism disorder** | 52 | 38.5% | 125 | 47.3% | 144 | 40.1% | 340 | 41.1% | 53 | 46.5% |
| **Liver disease** | 12 | 8.9% | 19 | 7.2% | 20 | 5.6% | 44 | 5.3% | 5 | 4.4% |
| **PAD** | 7 | 5.2% | 35 | 13.3% | 22 | 6.1% | 49 | 5.9% | 7 | 6.1% |
| **CHD** | 19 | 14.1% | 45 | 17.0% | 50 | 13.9% | 122 | 14.8% | 17 | 14.9% |
| **Myocardial infarction** | 1 | 0.7% | 4 | 1.5% | 11 | 3.1% | 13 | 1.6% | 3 | 2.6% |
| **Impaired glucose tolerance** | 8 | 5.9% | 7 | 2.7% | 7 | 1.9% | 26 | 3.1% | 5 | 4.4% |
| **IFG** | 1 | 0.7% | 2 | 0.8% | 1 | 0.3% | 10 | 1.2% | 0 | 0.0% |
| **T1DM** | 0 | 0.0% | 0 | 0.0% | 3 | 0.8% | 3 | 0.4% | 0 | 0.0% |
| **T2DM** | 47 | 34.8% | 98 | 37.1% | 114 | 31.8% | 262 | 31.7% | 42 | 36.8% |
| **Renal insufficiency** | 2 | 1.5% | 4 | 1.5% | 4 | 1.1% | 14 | 1.7% | 0 | 0.0% |
| **Urinary tract infection** | 3 | 2.2% | 4 | 1.5% | 3 | 0.8% | 7 | 0.8% | 2 | 1.8% |
| **Respiratory disease** | 5 | 3.7% | 6 | 2.3% | 12 | 3.3% | 30 | 3.6% | 6 | 5.3% |
| **Pulmonary infection** | 5 | 3.7% | 6 | 2.3% | 12 | 3.3% | 30 | 3.6% | 6 | 5.3% |
| **DVT** | 2 | 1.5% | 1 | 0.4% | 0 | 0.0% | 3 | 0.4% | 0 | 0.0% |
| **Epilepsy** | 1 | 0.7% | 0 | 0.0% | 0 | 0.0% | 5 | 0.6% | 1 | 0.9% |
| **Bleeding** | 4 | 3.0% | 1 | 0.4% | 1 | 0.3% | 6 | 0.7% | 2 | 1.8% |
| **sECAS** | 2 | 1.5% | 13 | 4.9% | 17 | 4.7% | 30 | 3.6% | 6 | 5.3% |
| **sICAS** | 27 | 20.0% | 67 | 25.4% | 91 | 25.3% | 201 | 24.3% | 28 | 24.6% |
| **TOAST-Large artery atherosclerosis** | 38 | 28.1% | 80 | 30.3% | 105 | 29.2% | 227 | 27.4% | 30 | 26.3% |
| **TOAST-Small vascular occlusion** | 33 | 24.4% | 73 | 27.7% | 89 | 24.8% | 222 | 26.8% | 24 | 21.1% |
| **TOAST-Undetermined causes** | 64 | 47.4% | 111 | 42.0% | 165 | 46.0% | 378 | 45.7% | 60 | 52.6% |
| **Anti-platelet therapy** | 132 | 97.8% | 256 | 97.0% | 354 | 98.6% | 811 | 98.1% | 113 | 99.1% |
| **Aspirin** | 121 | 89.6% | 240 | 90.9% | 318 | 88.6% | 726 | 87.8% | 102 | 89.5% |
| **Clopidogrel** | 79 | 58.5% | 171 | 64.8% | 241 | 67.1% | 518 | 62.6% | 80 | 70.2% |
| **Dipyridamole** | 0 | 0.0% | 0 | 0.0% | 0 | 0.0% | 3 | 0.4% | 0 | 0.0% |
| **Cilostazol** | 0 | 0.0% | 2 | 0.8% | 7 | 1.9% | 4 | 0.5% | 0 | 0.0% |
| **Hypoglycemic treatment** | 38 | 28.1% | 90 | 34.1% | 93 | 25.9% | 222 | 26.8% | 37 | 32.5% |
| **Antihypertensive treatment** | 67 | 49.6% | 132 | 50.0% | 140 | 39.0% | 427 | 51.6% | 47 | 41.2% |
| **Lipid-lowering drugs** | 130 | 96.3% | 259 | 98.1% | 353 | 98.3% | 809 | 97.8% | 109 | 95.6% |
| **Hemorrhagic stroke in hospital** | 0 | 0.0% | 0 | 0.0% | 0 | 0.0% | 2 | 0.2% | 0 | 0.0% |
| **Ischemic stroke in hospital** | 8 | 5.9% | 16 | 6.1% | 13 | 3.6% | 30 | 3.6% | 3 | 2.6% |
| **Hemorrhagic transformation** | 3 | 2.2% | 1 | 0.4% | 1 | 0.3% | 6 | 0.7% | 0 | 0.0% |
| **Systemic embolic events in hospital** | 0 | 0.0% | 0 | 0.0% | 0 | 0.0% | 0 | 0.0% | 0 | 0.0% |
| **Death in hospital** | 0 | 0.0% | 1 | 0.4% | 0 | 0.0% | 1 | 0.1% | 0 | 0.0% |
| **Hemorrhagic stroke at 3 months** | 0 | 0.0% | 1 | 0.4% | 0 | 0.0% | 4 | 0.5% | 0 | 0.0% |
| **Combined vascular event at 3 months** | 9 | 6.7% | 23 | 8.7% | 21 | 5.8% | 46 | 5.6% | 5 | 4.4% |
| **Death at 3 months** | 0 | 0.0% | 3 | 1.1% | 1 | 0.3% | 7 | 0.8% | 1 | 0.9% |
| **Ischemic stroke at 3 months** | 9 | 6.7% | 21 | 8.0% | 21 | 5.8% | 44 | 5.3% | 5 | 4.4% |
| **mRS at 3 mongths** | 14 | 10.4% | 45 | 17.0% | 37 | 10.3% | 78 | 9.4% | 17 | 14.9% |
| **Stroke at 3 months** | 9 | 6.7% | 22 | 8.3% | 21 | 5.8% | 46 | 5.6% | 5 | 4.4% |
| **Combined vascular event at 6 months** | 10 | 7.4% | 27 | 10.2% | 29 | 8.1% | 62 | 7.5% | 8 | 7.0% |
| **Death at 6 months** | 0 | 0.0% | 6 | 2.3% | 2 | 0.6% | 12 | 1.5% | 1 | 0.9% |
| **mRS at 6 mongths** | 9 | 6.7% | 40 | 15.2% | 42 | 11.7% | 72 | 8.7% | 12 | 10.5% |
| **Stroke at 6 months** | 10 | 7.4% | 26 | 9.8% | 29 | 8.1% | 61 | 7.4% | 8 | 7.0% |
| **Combined vascular event at 1 year** | 13 | 9.6% | 32 | 12.1% | 34 | 9.5% | 79 | 9.6% | 14 | 12.3% |
| **Death at 1 year** | 1 | 0.7% | 7 | 2.7% | 6 | 1.7% | 19 | 2.3% | 1 | 0.9% |
| **Stroke at 1 year** | 12 | 8.9% | 31 | 11.7% | 34 | 9.5% | 77 | 9.3% | 13 | 11.4% |
| **mRS at 1 year** | 8 | 5.9% | 42 | 15.9% | 36 | 10.0% | 82 | 9.9% | 10 | 8.8% |

|  | **Cluster 26** | | **Cluster 27** | | **Cluster 28** | | **Cluster 29** | | **Cluster 30** | |
| --- | --- | --- | --- | --- | --- | --- | --- | --- | --- | --- |
|  | **Mean (SD)** | **Median (IQR)** | **Mean (SD)** | **Median**  **(IQR)** | **Mean (SD)** | **Median (IQR)** | **Mean (SD)** | **Median (IQR)** | **Mean (SD)** | **Median (IQR)** |
| **Age** | 63.1  (11.5) | 64  (54,72) | 59.2  (11.4) | 60  (52,67.3) | 56.2  (10.4) | 56  (49,63) | 58.3  (9.4) | 58  (52,64.8) | 60.2  (9.1) | 61  (54,67) |
| **DBP (mmHg)** | 87.8  (12.9) | 87.5  (80,94) | 92.9  (15) | 90.5  (81,100) | 90.8  (13.2) | 90  (81,99.5) | 87.9  (12.4) | 86  (80,95) | 89  (13.6) | 87.5  (80,98.6) |
| **SBP (mmHg)** | 152.4  (23.6) | 150  (137.5,163.5) | 154.7  (23.8) | 154.8  (133.5,171.1) | 151.7  (21.4) | 149  (137.5,165.5) | 153.4  (22.8) | 150.8  (136.1,167.5) | 153.8  (22.4) | 151  (140,167.1) |
| **BMI (Kg/m2)** | 24.5  (3.4) | 24  (22,26.6) | 25.1  (3.0) | 24.7  (23.1,26.8) | 25.7  (3.1) | 25.4  (23.6,27.6) | 24.9  (3.2) | 24.54  (23.1,26.8) | 24.7  (3.0) | 24.6  (22.7,26.3) |
|  | **Event**  **(n)** | **Ratio**  **(%)** | **Event**  **(n)** | **Ratio**  **(%)** | **Event**  **(n)** | **Ratio**  **(%)** | **Event**  **(n)** | **Ratio**  **(%)** | **Event**  **(n)** | **Ratio**  **(%)** |
| **Gender, Female** | 63 | 42.3% | 14 | 9.7% | 117 | 16.5% | 80 | 37.4% | 102 | 41.1% |
| **Drinking** | 18 | 12.1% | 39 | 27.1% | 137 | 19.4% | 33 | 15.4% | 43 | 17.3% |
| **Smoking** | 40 | 26.8% | 53 | 36.8% | 301 | 42.6% | 69 | 32.2% | 68 | 27.4% |
| **History of stroke** | 33 | 22.1% | 28 | 19.4% | 146 | 20.7% | 50 | 23.4% | 45 | 18.1% |
| **Family history of diabetes** | 5 | 3.4% | 14 | 9.7% | 49 | 6.9% | 44 | 20.6% | 17 | 6.9% |
| **Family history of hypertension** | 31 | 20.8% | 40 | 27.8% | 187 | 26.4% | 62 | 29.0% | 44 | 17.7% |
| **Family history of hyperlipidemia** | 2 | 1.3% | 1 | 0.7% | 15 | 2.1% | 11 | 5.1% | 3 | 1.2% |
| **Family history of stroke** | 20 | 13.4% | 24 | 16.7% | 122 | 17.3% | 37 | 17.3% | 31 | 12.5% |
| **Hypertension** | 122 | 81.9% | 120 | 83.3% | 533 | 75.4% | 153 | 71.5% | 185 | 74.6% |
| **Lipid metabolism disorder** | 93 | 62.4% | 61 | 42.4% | 299 | 42.3% | 96 | 44.9% | 113 | 45.6% |
| **Liver disease** | 12 | 8.1% | 11 | 7.6% | 57 | 8.1% | 6 | 2.8% | 13 | 5.2% |
| **PAD** | 6 | 4.0% | 4 | 2.8% | 40 | 5.7% | 13 | 6.1% | 4 | 1.6% |
| **CHD** | 19 | 12.8% | 21 | 14.6% | 77 | 10.9% | 41 | 19.2% | 28 | 11.3% |
| **Myocardial infarction** | 2 | 1.3% | 5 | 3.5% | 6 | 0.8% | 4 | 1.9% | 2 | 0.8% |
| **Impaired glucose tolerance** | 6 | 4.0% | 5 | 3.5% | 21 | 3.0% | 2 | 0.9% | 5 | 2.0% |
| **IFG** | 0 | 0.0% | 1 | 0.7% | 9 | 1.3% | 1 | 0.5% | 3 | 1.2% |
| **T1DM** | 0 | 0.0% | 0 | 0.0% | 0 | 0.0% | 0 | 0.0% | 0 | 0.0% |
| **T2DM** | 63 | 42.3% | 43 | 29.9% | 215 | 30.4% | 186 | 86.9% | 75 | 30.2% |
| **Renal insufficiency** | 1 | 0.7% | 2 | 1.4% | 3 | 0.4% | 0 | 0.0% | 2 | 0.8% |
| **Urinary tract infection** | 3 | 2.0% | 0 | 0.0% | 8 | 1.1% | 5 | 2.3% | 1 | 0.4% |
| **Respiratory disease** | 10 | 6.7% | 7 | 4.9% | 9 | 1.3% | 3 | 1.4% | 9 | 3.6% |
| **Pulmonary infection** | 10 | 6.7% | 7 | 4.9% | 9 | 1.3% | 3 | 1.4% | 9 | 3.6% |
| **DVT** | 1 | 0.7% | 0 | 0.0% | 2 | 0.3% | 1 | 0.5% | 0 | 0.0% |
| **Epilepsy** | 4 | 2.7% | 0 | 0.0% | 2 | 0.3% | 0 | 0.0% | 1 | 0.4% |
| **Bleeding** | 2 | 1.3% | 3 | 2.1% | 5 | 0.7% | 4 | 1.9% | 2 | 0.8% |
| **sECAS** | 10 | 6.7% | 9 | 6.3% | 24 | 3.4% | 5 | 2.3% | 9 | 3.6% |
| **sICAS** | 45 | 30.2% | 44 | 30.6% | 151 | 21.4% | 46 | 21.5% | 57 | 23.0% |
| **TOAST-Large artery atherosclerosis** | 48 | 32.2% | 46 | 31.9% | 185 | 26.2% | 57 | 26.6% | 66 | 26.6% |
| **TOAST-Small vascular occlusion** | 29 | 19.5% | 30 | 20.8% | 196 | 27.7% | 50 | 23.4% | 72 | 29.0% |
| **TOAST-Undetermined causes** | 72 | 48.3% | 68 | 47.2% | 326 | 46.1% | 107 | 50.0% | 110 | 44.4% |
| **Anti-platelet therapy** | 147 | 98.7% | 142 | 98.6% | 687 | 97.2% | 210 | 98.1% | 245 | 98.8% |
| **Aspirin** | 128 | 85.9% | 129 | 89.6% | 620 | 87.7% | 197 | 92.1% | 230 | 92.7% |
| **Clopidogrel** | 94 | 63.1% | 86 | 59.7% | 469 | 66.3% | 137 | 64.0% | 163 | 65.7% |
| **Dipyridamole** | 0 | 0.0% | 0 | 0.0% | 1 | 0.1% | 2 | 0.9% | 1 | 0.4% |
| **Cilostazol** | 0 | 0.0% | 1 | 0.7% | 4 | 0.6% | 2 | 0.9% | 1 | 0.4% |
| **Hypoglycemic treatment** | 52 | 34.9% | 36 | 25.0% | 184 | 26.0% | 172 | 80.4% | 72 | 29.0% |
| **Antihypertensive treatment** | 72 | 48.3% | 71 | 49.3% | 321 | 45.4% | 100 | 46.7% | 122 | 49.2% |
| **Lipid-lowering drugs** | 145 | 97.3% | 140 | 97.2% | 674 | 95.3% | 209 | 97.7% | 243 | 98.0% |
| **Hemorrhagic stroke in hospital** | 0 | 0.0% | 0 | 0.0% | 1 | 0.1% | 0 | 0.0% | 1 | 0.4% |
| **Ischemic stroke in hospital** | 9 | 6.0% | 4 | 2.8% | 30 | 4.2% | 14 | 6.5% | 14 | 5.6% |
| **Hemorrhagic transformation** | 1 | 0.7% | 2 | 1.4% | 3 | 0.4% | 1 | 0.5% | 0 | 0.0% |
| **Systemic embolic events in hospital** | 0 | 0.0% | 1 | 0.7% | 1 | 0.1% | 0 | 0.0% | 0 | 0.0% |
| **Death in hospital** | 0 | 0.0% | 0 | 0.0% | 0 | 0.0% | 0 | 0.0% | 2 | 0.8% |
| **Hemorrhagic stroke at 3 months** | 0 | 0.0% | 0 | 0.0% | 3 | 0.4% | 0 | 0.0% | 1 | 0.4% |
| **Combined vascular event at 3 months** | 11 | 7.4% | 6 | 4.2% | 43 | 6.1% | 17 | 7.9% | 17 | 6.9% |
| **Death at 3 months** | 2 | 1.3% | 2 | 1.4% | 1 | 0.1% | 3 | 1.4% | 3 | 1.2% |
| **Ischemic stroke at 3 months** | 10 | 6.7% | 6 | 4.2% | 39 | 5.5% | 15 | 7.0% | 16 | 6.5% |
| **mRS at 3 mongths** | 32 | 21.5% | 16 | 11.1% | 61 | 8.6% | 30 | 14.0% | 33 | 13.3% |
| **Stroke at 3 months** | 10 | 6.7% | 6 | 4.2% | 42 | 5.9% | 15 | 7.0% | 17 | 6.9% |
| **Combined vascular event at 6 months** | 12 | 8.1% | 9 | 6.3% | 54 | 7.6% | 20 | 9.3% | 20 | 8.1% |
| **Death at 6 months** | 2 | 1.3% | 2 | 1.4% | 2 | 0.3% | 4 | 1.9% | 4 | 1.6% |
| **mRS at 6 mongths** | 25 | 16.8% | 12 | 8.3% | 48 | 6.8% | 28 | 13.1% | 28 | 11.3% |
| **Stroke at 6 months** | 11 | 7.4% | 9 | 6.3% | 53 | 7.5% | 18 | 8.4% | 20 | 8.1% |
| **Combined vascular event at 1 year** | 14 | 9.4% | 13 | 9.0% | 67 | 9.5% | 24 | 11.2% | 24 | 9.7% |
| **Death at 1 year** | 5 | 3.4% | 3 | 2.1% | 7 | 1.0% | 5 | 2.3% | 6 | 2.4% |
| **Stroke at 1 year** | 12 | 8.1% | 13 | 9.0% | 65 | 9.2% | 22 | 10.3% | 24 | 9.7% |
| **mRS at 1 year** | 21 | 14.1% | 14 | 9.7% | 45 | 6.4% | 30 | 14.0% | 26 | 10.5% |

**Supplementary Table 5. Risk of stroke recurrence at 3 months in all clusters.**

| **Cluster** | **Number** | **Stroke recurrence at 3 months** | | | | |
| --- | --- | --- | --- | --- | --- | --- |
|  |  | **Events, n (%)** | **HR (95% CI)** | **P value** | **Adjust HR (95% CI)** | **P value** |
| **C1** | 53 | 7 (13.21%) | 2.341 (1.110, 4.937) | 0.026 | 2.206 (1.043, 4.665) | 0.038 |
| **C2** | 70 | 7 (10.00%) | 1.715 (0.813, 3.618) | 0.157 | 1.736 (0.822, 3.665) | 0.148 |
| **C3** | 194 | 21 (10.82%) | 1.848 (1.193, 2.862) | 0.006 | 1.914 (1.235, 2.966) | 0.004 |
| **C4** | 308 | 26 (8.44%) | 1.434 (0.965, 2.129) | 0.074 | 1.339 (0.899, 1.996) | 0.151 |
| **C5** | 49 | 3 (6.12%) | 1.015 (0.326, 3.160) | 0.979 | 0.970 (0.310, 3.032) | 0.958 |
| **C6** | 88 | 2 (2.27%) | 0.364 (0.091, 1.459) | 0.154 | 0.352 (0.088, 1.413) | 0.141 |
| **C7** | 153 | 17 (11.11%) | 1.910 (1.177, 3.099) | 0.009 | 1.694 (1.039, 2.764) | 0.035 |
| **C8** | 81 | 3 (3.70%) | 0.597 (0.192, 1.858) | 0.373 | 0.611 (0.196, 1.903) | 0.396 |
| **C9** | 183 | 13 (7.10%) | 1.189 (0.685, 2.063) | 0.539 | 1.370 (0.785, 2.388) | 0.268 |
| **C10** | 211 | 7 (3.32%) | 0.533 (0.253, 1.124) | 0.098 | 0.530 (0.251, 1.119) | 0.096 |
| **C11** | 677 | 39 (5.76%) | 0.935 (0.674, 1.297) | 0.687 | 0.943 (0.677, 1.315) | 0.731 |
| **C12** | 991 | 55 (5.55%) | 0.897 (0.677, 1.189) | 0.450 | 0.869 (0.651, 1.159) | 0.339 |
| **C13** | 569 | 30 (5.27%) | 0.848 (0.585, 1.227) | 0.381 | 0.846 (0.583, 1.228) | 0.379 |
| **C14** | 125 | 5 (4.00%) | 0.645 (0.267, 1.556) | 0.329 | 0.681 (0.282, 1.646) | 0.394 |
| **C15** | 101 | 3 (2.97%) | 0.472 (0.152, 1.469) | 0.195 | 0.482 (0.155, 1.501) | 0.208 |
| **C16** | 128 | 1 (0.78%) | 0.122 (0.017, 0.868) | 0.036 | 0.119 (0.017, 0.850) | 0.034 |
| **C17** | 128 | 12 (9.38%) | 1.573 (0.887, 2.790) | 0.122 | 1.611 (0.906, 2.867) | 0.105 |
| **C18** | 158 | 10 (6.33%) | 1.045 (0.558, 1.955) | 0.891 | 1.120 (0.595, 2.110) | 0.725 |
| **C19** | 178 | 11 (6.18%) | 1.016 (0.559, 1.848) | 0.957 | 0.991 (0.542, 1.811) | 0.976 |
| **C20** | 89 | 4 (4.49%) | 0.727 (0.272, 1.946) | 0.526 | 0.722 (0.270, 1.934) | 0.517 |
| **C21** | 135 | 9 (6.67%) | 1.111 (0.574, 2.148) | 0.755 | 1.094 (0.565, 2.118) | 0.789 |
| **C22** | 264 | 22 (8.33%) | 1.401 (0.913, 2.150) | 0.123 | 1.335 (0.868, 2.051) | 0.188 |
| **C23** | 359 | 21 (5.85%) | 0.951 (0.614, 1.474) | 0.824 | 0.961 (0.620, 1.490) | 0.859 |
| **C24** | 827 | 46 (5.56%) | 0.897 (0.662, 1.217) | 0.486 | 0.880 (0.648, 1.194) | 0.411 |
| **C25** | 114 | 5 (4.39%) | 0.711 (0.295, 1.716) | 0.448 | 0.704 (0.291, 1.700) | 0.435 |
| **C26** | 149 | 10 (6.71%) | 1.113 (0.595, 2.082) | 0.738 | 1.045 (0.557, 1.961) | 0.891 |
| **C27** | 144 | 6 (4.17%) | 0.671 (0.300, 1.502) | 0.332 | 0.684 (0.305, 1.535) | 0.357 |
| **C28** | 707 | 42 (5.94%) | 0.971 (0.707, 1.333) | 0.853 | 1.077 (0.781, 1.486) | 0.650 |
| **C29** | 214 | 15 (7.01%) | 1.171 (0.700, 1.959) | 0.547 | 1.115 (0.659, 1.886) | 0.686 |
| **C30** | 248 | 17 (6.85%) | 1.131 (0.697, 1.835) | 0.620 | 1.139 (0.700, 1.851) | 0.600 |

Adjust: Age, gender, hypertension, diabetes, coronary heart disease, peripheral arterial disease, hyperlipemia, smoke, drink, history of stroke

**Supplementary Table 6. Risk of stroke recurrence at 6 months in all clusters.**

| **Cluster** | **Number** | **Stroke recurrence at 6 months** | | | | |
| --- | --- | --- | --- | --- | --- | --- |
|  |  | **Events, n (%)** | **HR (95% CI)** | **P value** | **Adjust HR (95% CI)** | **P value** |
| **C1** | 53 | 11 (20.75%) | 3.027 (1.667, 5.497) | <0.001 | 2.863 (1.573, 5.210) | 0.001 |
| **C2** | 70 | 9 (12.86%) | 1.765 (0.914, 3.410) | 0.091 | 1.782 (0.922, 3.445) | 0.086 |
| **C3** | 194 | 27 (13.92%) | 1.898 (1.290, 2.792) | 0.001 | 1.958 (1.330, 2.883) | 0.001 |
| **C4** | 308 | 33 (10.71%) | 1.438 (1.012, 2.043) | 0.043 | 1.342 (0.942, 1.913) | 0.103 |
| **C5** | 49 | 5 (10.20%) | 1.347 (0.559, 3.249) | 0.507 | 1.280 (0.529, 3.102) | 0.584 |
| **C6** | 88 | 4 (4.55%) | 0.574 (0.215, 1.535) | 0.269 | 0.558 (0.209, 1.495) | 0.246 |
| **C7** | 153 | 20 (13.07%) | 1.774 (1.136, 2.771) | 0.012 | 1.551 (0.989, 2.432) | 0.056 |
| **C8** | 81 | 7 (8.64%) | 1.099 (0.521, 2.315) | 0.805 | 1.136 (0.539, 2.395) | 0.737 |
| **C9** | 183 | 17 (9.29%) | 1.223 (0.755, 1.981) | 0.413 | 1.395 (0.858, 2.270) | 0.180 |
| **C10** | 211 | 9 (4.27%) | 0.536 (0.278, 1.036) | 0.064 | 0.524 (0.271, 1.012) | 0.054 |
| **C11** | 677 | 46 (6.79%) | 0.861 (0.638, 1.164) | 0.331 | 0.865 (0.638, 1.173) | 0.352 |
| **C12** | 991 | 68 (6.86%) | 0.870 (0.676, 1.120) | 0.279 | 0.846 (0.653, 1.095) | 0.205 |
| **C13** | 569 | 38 (6.68%) | 0.842 (0.606, 1.170) | 0.305 | 0.846 (0.608, 1.179) | 0.323 |
| **C14** | 125 | 7 (5.60%) | 0.709 (0.337, 1.494) | 0.366 | 0.738 (0.350, 1.558) | 0.426 |
| **C15** | 101 | 4 (3.96%) | 0.494 (0.185, 1.320) | 0.160 | 0.508 (0.190, 1.361) | 0.178 |
| **C16** | 128 | 4 (3.12%) | 0.384 (0.143, 1.025) | 0.056 | 0.378 (0.141, 1.011) | 0.053 |
| **C17** | 128 | 14 (10.94%) | 1.445 (0.850, 2.455) | 0.174 | 1.500 (0.880, 2.555) | 0.136 |
| **C18** | 158 | 12 (7.59%) | 0.987 (0.557, 1.748) | 0.965 | 1.076 (0.604, 1.917) | 0.804 |
| **C19** | 178 | 12 (6.74%) | 0.868 (0.490, 1.538) | 0.628 | 0.845 (0.475, 1.503) | 0.566 |
| **C20** | 89 | 4 (4.49%) | 0.567 (0.212, 1.516) | 0.258 | 0.556 (0.208, 1.489) | 0.243 |
| **C21** | 135 | 10 (7.41%) | 0.967 (0.517, 1.806) | 0.915 | 0.966 (0.517, 1.806) | 0.913 |
| **C22** | 264 | 26 (9.85%) | 1.300 (0.878, 1.926) | 0.191 | 1.231 (0.830, 1.827) | 0.302 |
| **C23** | 359 | 29 (8.08%) | 1.037 (0.714, 1.506) | 0.848 | 1.039 (0.715, 1.511) | 0.840 |
| **C24** | 827 | 61 (7.38%) | 0.942 (0.722, 1.227) | 0.656 | 0.920 (0.706, 1.201) | 0.541 |
| **C25** | 114 | 8 (7.02%) | 0.895 (0.445, 1.797) | 0.755 | 0.883 (0.439, 1.776) | 0.728 |
| **C26** | 149 | 11 (7.38%) | 0.956 (0.527, 1.736) | 0.883 | 0.888 (0.488, 1.617) | 0.698 |
| **C27** | 144 | 9 (6.25%) | 0.792 (0.410, 1.531) | 0.489 | 0.811 (0.419, 1.569) | 0.533 |
| **C28** | 707 | 53 (7.50%) | 0.961 (0.725, 1.274) | 0.781 | 1.063 (0.798, 1.415) | 0.677 |
| **C29** | 214 | 18 (8.41%) | 1.102 (0.689, 1.762) | 0.684 | 1.062 (0.658, 1.714) | 0.807 |
| **C30** | 248 | 20 (8.06%) | 1.044 (0.668, 1.630) | 0.851 | 1.060 (0.678, 1.659) | 0.797 |

Adjust: Age, gender, hypertension, diabetes, coronary heart disease, peripheral arterial disease, hyperlipemia, smoke, drink, history of stroke

**Table S7. Risk of stroke recurrence at 12 months in all clusters.**

| **Cluster** | **Number** | **Stroke recurrence at 12 months** | | | | |
| --- | --- | --- | --- | --- | --- | --- |
|  |  | **Events, n (%)** | **HR (95% CI)** | **P value** | **Adjust HR (95% CI)** | **P value** |
| **C1** | 53 | 12 (22.64%) | 2.800 (1.583, 4.954) | <0.001 | 2.601 (1.467, 4.611) | 0.001 |
| **C2** | 70 | 11 (15.71%) | 1.777 (0.980, 3.223) | 0.058 | 1.766 (0.973, 3.206) | 0.061 |
| **C3** | 194 | 30 (15.46%) | 1.735 (1.204, 2.500) | 0.003 | 1.780 (1.235, 2.567) | 0.002 |
| **C4** | 308 | 40 (12.99%) | 1.434 (1.043, 1.973) | 0.027 | 1.336 (0.969, 1.842) | 0.077 |
| **C5** | 49 | 7 (14.29%) | 1.573 (0.747, 3.312) | 0.233 | 1.522 (0.720, 3.218) | 0.271 |
| **C6** | 88 | 8 (9.09%) | 0.944 (0.470, 1.895) | 0.871 | 0.930 (0.463, 1.870) | 0.840 |
| **C7** | 153 | 26 (16.99%) | 1.902 (1.286, 2.814) | 0.001 | 1.649 (1.111, 2.448) | 0.013 |
| **C8** | 81 | 7 (8.64%) | 0.891 (0.423, 1.876) | 0.762 | 0.930 (0.442, 1.959) | 0.849 |
| **C9** | 183 | 21 (11.48%) | 1.229 (0.796, 1.896) | 0.353 | 1.377 (0.888, 2.133) | 0.153 |
| **C10** | 211 | 15 (7.11%) | 0.730 (0.438, 1.217) | 0.227 | 0.705 (0.422, 1.176) | 0.180 |
| **C11** | 677 | 49 (7.24%) | 0.737 (0.551, 0.984) | 0.039 | 0.727 (0.542, 0.974) | 0.032 |
| **C12** | 991 | 83 (8.38%) | 0.862 (0.686, 1.083) | 0.203 | 0.835 (0.661, 1.055) | 0.131 |
| **C13** | 569 | 53 (9.31%) | 0.962 (0.728, 1.273) | 0.788 | 0.970 (0.731, 1.286) | 0.830 |
| **C14** | 125 | 10 (8.00%) | 0.825 (0.442, 1.541) | 0.547 | 0.856 (0.458, 1.600) | 0.627 |
| **C15** | 101 | 6 (5.94%) | 0.605 (0.271, 1.351) | 0.220 | 0.617 (0.276, 1.379) | 0.239 |
| **C16** | 128 | 6 (4.69%) | 0.466 (0.209, 1.041) | 0.063 | 0.467 (0.209, 1.043) | 0.063 |
| **C17** | 128 | 15 (11.72%) | 1.257 (0.754, 2.096) | 0.381 | 1.303 (0.779, 2.177) | 0.313 |
| **C18** | 158 | 12 (7.59%) | 0.802 (0.453, 1.419) | 0.449 | 0.897 (0.505, 1.595) | 0.712 |
| **C19** | 178 | 13 (7.30%) | 0.758 (0.438, 1.312) | 0.323 | 0.742 (0.427, 1.289) | 0.290 |
| **C20** | 89 | 5 (5.62%) | 0.573 (0.238, 1.381) | 0.215 | 0.564 (0.234, 1.360) | 0.202 |
| **C21** | 135 | 12 (8.89%) | 0.944 (0.534, 1.670) | 0.843 | 0.960 (0.543, 1.700) | 0.890 |
| **C22** | 264 | 31 (11.74%) | 1.260 (0.879, 1.805) | 0.208 | 1.208 (0.842, 1.734) | 0.305 |
| **C23** | 359 | 34 (9.47%) | 0.987 (0.700, 1.393) | 0.941 | 0.992 (0.702, 1.401) | 0.963 |
| **C24** | 827 | 77 (9.31%) | 0.971 (0.767, 1.230) | 0.809 | 0.956 (0.754, 1.212) | 0.712 |
| **C25** | 114 | 13 (11.40%) | 1.190 (0.687, 2.059) | 0.535 | 1.187 (0.685, 2.056) | 0.541 |
| **C26** | 149 | 12 (8.05%) | 0.842 (0.476, 1.490) | 0.555 | 0.797 (0.450, 1.414) | 0.438 |
| **C27** | 144 | 13 (9.03%) | 0.935 (0.541, 1.619) | 0.812 | 0.946 (0.546, 1.641) | 0.844 |
| **C28** | 707 | 65 (9.19%) | 0.957 (0.742, 1.234) | 0.733 | 1.045 (0.807, 1.352) | 0.741 |
| **C29** | 214 | 22 (10.28%) | 1.094 (0.716, 1.672) | 0.678 | 1.073 (0.695, 1.655) | 0.750 |
| **C30** | 248 | 24 (9.68%) | 1.018 (0.678, 1.529) | 0.931 | 1.044 (0.694, 1.570) | 0.837 |

Adjust: Age, gender, hypertension, diabetes, coronary heart disease, peripheral arterial disease, hyperlipemia, smoke, drink, history of stroke

**Supplementary Table 8. Risk of mortality at 3 months in all clusters.**

| **Cluster** | **Number** | **Mortality 3 months** | | | | | |
| --- | --- | --- | --- | --- | --- | --- | --- |
|  |  | **Events, n (%)** | **HR (95% CI)** | **P value** | **Adjust HR (95% CI)** | **P value** |  |
| **C1** | 53 | 5 (9.43%) | - | - | - | - |  |
| **C2** | 70 | 6 (8.57%) | 8.362 (3.649, 19.163) | <0.001 | 8.064 (3.500, 18.578) | <0.001 |  |
| **C3** | 194 | 7 (3.61%) | 3.439 (1.588, 7.445) | 0.002 | 3.531 (1.627, 7.663) | 0.001 |  |
| **C4** | 308 | 10 (3.25%) | 3.149 (1.629, 6.085) | 0.001 | 2.389 (1.222, 4.670) | 0.011 |  |
| **C5** | 49 | 3 (6.12%) | 5.834 (1.844, 18.454) | 0.003 | 6.663 (2.052, 21.631) | 0.002 |  |
| **C6** | 88 | 1 (1.14%) | 1.010 (0.141, 7.253) | 0.992 | 0.895 (0.124, 6.442) | 0.912 |  |
| **C7** | 153 | 5 (3.27%) | 3.011 (1.221, 7.428) | 0.017 | 2.165 (0.866, 5.411) | 0.098 |  |
| **C8** | 81 | 1 (1.23%) | 1.089 (0.152, 7.822) | 0.932 | 1.147 (0.159, 8.242) | 0.892 |  |
| **C9** | 183 | 3 (1.64%) | 1.467 (0.464, 4.639) | 0.515 | 1.669 (0.523, 5.329) | 0.387 |  |
| **C10** | 211 | 2 (0.95%) | 0.836 (0.206, 3.399) | 0.803 | 0.820 (0.201, 3.342) | 0.782 |  |
| **C11** | 677 | 5 (0.74%) | 0.631 (0.256, 1.556) | 0.317 | 0.518 (0.209, 1.286) | 0.156 |  |
| **C12** | 991 | 5 (0.50%) | 0.413 (0.167, 1.018) | 0.055 | 0.425 (0.170, 1.059) | 0.066 |  |
| **C13** | 569 | 5 (0.88%) | 0.759 (0.308, 1.873) | 0.550 | 0.703 (0.284, 1.743) | 0.447 |  |
| **C14** | 125 | 0 (0.00%) | - | - | - | - |  |
| **C15** | 101 | 0 (0.00%) | - | - | - | - |  |
| **C16** | 128 | 1 (0.78%) | 0.681 (0.095, 4.888) | 0.702 | 0.677 (0.094, 4.863) | 0.698 |  |
| **C17** | 128 | 1 (0.78%) | 0.684 (0.095, 4.913) | 0.706 | 0.794 (0.110, 5.720) | 0.819 |  |
| **C18** | 158 | 2 (1.27%) | 1.127 (0.277, 4.581) | 0.867 | 1.724 (0.418, 7.100) | 0.451 |  |
| **C19** | 178 | 1 (0.56%) | 0.491 (0.068, 3.525) | 0.479 | 0.622 (0.086, 4.519) | 0.639 |  |
| **C20** | 89 | 1 (1.12%) | 0.991 (0.138, 7.116) | 0.993 | 0.979 (0.136, 7.045) | 0.983 |  |
| **C21** | 135 | 0 (0.00%) | - | - | - | - |  |
| **C22** | 264 | 3 (1.14%) | 1.005 (0.318, 3.178) | 0.994 | 0.945 (0.298, 2.999) | 0.924 |  |
| **C23** | 359 | 1 (0.28%) | 0.236 (0.033, 1.692) | 0.151 | 0.231 (0.032, 1.661) | 0.146 |  |
| **C24** | 827 | 7 (0.85%) | 0.727 (0.336, 1.574) | 0.419 | 0.666 (0.307, 1.445) | 0.304 |  |
| **C25** | 114 | 1 (0.88%) | 0.772 (0.107, 5.541) | 0.797 | 0.827 (0.115, 5.954) | 0.851 |  |
| **C26** | 149 | 2 (1.34%) | 1.191 (0.293, 4.839) | 0.807 | 1.163 (0.284, 4.760) | 0.834 |  |
| **C27** | 144 | 2 (1.39%) | 1.228 (0.302, 4.990) | 0.774 | 1.456 (0.356, 5.944) | 0.601 |  |
| **C28** | 707 | 1 (0.14%) | 0.114 (0.016, 0.819) | 0.031 | 0.141 (0.020, 1.019) | 0.052 |  |
| **C29** | 214 | 3 (1.40%) | 1.242 (0.393, 3.929) | 0.712 | 1.407 (0.432, 4.579) | 0.571 |  |
| **C30** | 248 | 3 (1.21%) | 1.072 (0.339, 3.392) | 0.906 | 1.298 (0.408, 4.132) | 0.659 |  |

Adjust: Age, gender, hypertension, diabetes, coronary heart disease, peripheral arterial disease, hyperlipemia, smoke, drink, history of stroke

**Supplementary Table 9. Risk of mortality at 6 months in all clusters.**

| **Cluster** | **Number** | **Mortality 6 months** | | | | |
| --- | --- | --- | --- | --- | --- | --- |
|  |  | **Events, n (%)** | **HR (95% CI)** | **P value** | **Adjust HR (95% CI)** | **P value** |
| **C1** | 53 | 8 (15.09%) | - | - | - | - |
| **C2** | 70 | 8 (11.43%) | 7.397 (3.619, 15.119) | <0.001 | 7.482 (3.649, 15.343) | <0.001 |
| **C3** | 194 | 10 (5.15%) | 3.221 (1.691, 6.136) | <0.001 | 3.243 (1.698, 6.192) | <0.001 |
| **C4** | 308 | 18 (5.84%) | 3.811 (2.319, 6.263) | <0.001 | 2.962 (1.783, 4.920) | <0.001 |
| **C5** | 49 | 6 (12.24%) | 7.797 (3.438, 17.682) | <0.001 | 7.542 (3.253, 17.485) | <0.001 |
| **C6** | 88 | 2 (2.27%) | 1.331 (0.329, 5.379) | 0.688 | 1.206 (0.298, 4.885) | 0.793 |
| **C7** | 153 | 8 (5.23%) | 3.178 (1.555, 6.496) | 0.002 | 2.078 (1.007, 4.290) | 0.048 |
| **C8** | 81 | 1 (1.23%) | 0.708 (0.099, 5.064) | 0.731 | 0.779 (0.109, 5.577) | 0.804 |
| **C9** | 183 | 4 (2.19%) | 1.274 (0.471, 3.445) | 0.634 | 1.503 (0.551, 4.100) | 0.426 |
| **C10** | 211 | 2 (0.95%) | 0.541 (0.134, 2.188) | 0.389 | 0.497 (0.123, 2.011) | 0.327 |
| **C11** | 677 | 6 (0.89%) | 0.488 (0.215, 1.107) | 0.086 | 0.413 (0.181, 0.941) | 0.035 |
| **C12** | 991 | 9 (0.91%) | 0.491 (0.250, 0.967) | 0.040 | 0.531 (0.267, 1.055) | 0.071 |
| **C13** | 569 | 6 (1.05%) | 0.588 (0.259, 1.333) | 0.203 | 0.586 (0.257, 1.335) | 0.203 |
| **C14** | 125 | 0 (0.00%) | - | - | - | - |
| **C15** | 101 | 0 (0.00%) | - | - | - | - |
| **C16** | 128 | 2 (1.56%) | 0.894 (0.221, 3.612) | 0.875 | 0.894 (0.221, 3.618) | 0.876 |
| **C17** | 128 | 3 (2.34%) | 1.358 (0.432, 4.267) | 0.600 | 1.587 (0.503, 5.014) | 0.431 |
| **C18** | 158 | 3 (1.90%) | 1.108 (0.353, 3.481) | 0.860 | 1.795 (0.566, 5.697) | 0.321 |
| **C19** | 178 | 1 (0.56%) | 0.319 (0.045, 2.282) | 0.255 | 0.359 (0.050, 2.584) | 0.309 |
| **C20** | 89 | 1 (1.12%) | 0.643 (0.090, 4.601) | 0.661 | 0.644 (0.090, 4.612) | 0.661 |
| **C21** | 135 | 0 (0.00%) | - | - | - | - |
| **C22** | 264 | 6 (2.27%) | 1.331 (0.587, 3.018) | 0.494 | 1.184 (0.521, 2.694) | 0.687 |
| **C23** | 359 | 2 (0.56%) | 0.308 (0.076, 1.246) | 0.099 | 0.305 (0.075, 1.233) | 0.096 |
| **C24** | 827 | 12 (1.45%) | 0.823 (0.455, 1.490) | 0.521 | 0.740 (0.408, 1.341) | 0.320 |
| **C25** | 114 | 1 (0.88%) | 0.500 (0.070, 3.579) | 0.491 | 0.510 (0.071, 3.657) | 0.503 |
| **C26** | 149 | 2 (1.34%) | 0.770 (0.191, 3.111) | 0.714 | 0.686 (0.169, 2.784) | 0.598 |
| **C27** | 144 | 2 (1.39%) | 0.797 (0.197, 3.221) | 0.750 | 0.935 (0.231, 3.788) | 0.925 |
| **C28** | 707 | 2 (0.28%) | 0.149 (0.037, 0.604) | 0.008 | 0.182 (0.045, 0.737) | 0.017 |
| **C29** | 214 | 4 (1.87%) | 1.079 (0.399, 2.920) | 0.880 | 1.118 (0.405, 3.085) | 0.829 |
| **C30** | 248 | 4 (1.61%) | 0.930 (0.344, 2.516) | 0.887 | 1.159 (0.426, 3.149) | 0.773 |

Adjust: Age, gender, hypertension, diabetes, coronary heart disease, peripheral arterial disease, hyperlipemia, smoke, drink, history of stroke

**Supplementary Table 10. Risk of mortality at 12 months in all clusters.**

| **Cluster** | **Number** | **Mortality 12 months** | | | | |
| --- | --- | --- | --- | --- | --- | --- |
|  |  | **Events, n (%)** | **HR (95% CI)** | **P value** | **Adjust HR (95% CI)** | **P value** |
| **C1** | 53 | 8 (15.09%) | 6.991 (3.446, 14.186) | <0.001 | 5.495 (2.695, 11.204) | <0.001 |
| **C2** | 70 | 8 (11.43%) | 4.973 (2.451, 10.091) | <0.001 | 4.867 (2.394, 9.895) | <0.001 |
| **C3** | 194 | 11 (5.67%) | 2.372 (1.291, 4.358) | 0.005 | 2.423 (1.317, 4.458) | 0.004 |
| **C4** | 308 | 25 (8.12%) | 3.608 (2.371, 5.489) | <0.001 | 2.676 (1.744, 4.106) | <0.001 |
| **C5** | 49 | 9 (18.37%) | 8.155 (4.177, 15.918) | <0.001 | 8.111 (4.079, 16.131) | <0.001 |
| **C6** | 88 | 2 (2.27%) | 0.904 (0.225, 3.642) | 0.888 | 0.815 (0.202, 3.287) | 0.774 |
| **C7** | 153 | 14 (9.15%) | 3.859 (2.241, 6.645) | <0.001 | 2.433 (1.400, 4.227) | 0.002 |
| **C8** | 81 | 1 (1.23%) | 0.477 (0.067, 3.400) | 0.460 | 0.534 (0.075, 3.812) | 0.532 |
| **C9** | 183 | 4 (2.19%) | 0.854 (0.317, 2.298) | 0.754 | 1.004 (0.371, 2.717) | 0.994 |
| **C10** | 211 | 2 (0.95%) | 0.364 (0.090, 1.467) | 0.155 | 0.326 (0.081, 1.313) | 0.115 |
| **C11** | 677 | 12 (1.77%) | 0.672 (0.375, 1.206) | 0.183 | 0.546 (0.303, 0.984) | 0.044 |
| **C12** | 991 | 14 (1.41%) | 0.520 (0.302, 0.896) | 0.019 | 0.579 (0.333, 1.005) | 0.052 |
| **C13** | 569 | 11 (1.93%) | 0.737 (0.401, 1.354) | 0.326 | 0.741 (0.402, 1.367) | 0.337 |
| **C14** | 125 | 1 (0.80%) | 0.308 (0.043, 2.199) | 0.240 | 0.330 (0.046, 2.361) | 0.270 |
| **C15** | 101 | 0 (0.00%) | - | - | - | - |
| **C16** | 128 | 2 (1.56%) | 0.600 (0.149, 2.414) | 0.472 | 0.628 (0.156, 2.529) | 0.512 |
| **C17** | 128 | 4 (3.12%) | 1.229 (0.457, 3.308) | 0.683 | 1.488 (0.550, 4.025) | 0.433 |
| **C18** | 158 | 4 (2.53%) | 1.008 (0.375, 2.714) | 0.987 | 1.632 (0.601, 4.433) | 0.337 |
| **C19** | 178 | 1 (0.56%) | 0.214 (0.030, 1.527) | 0.124 | 0.257 (0.036, 1.844) | 0.177 |
| **C20** | 89 | 3 (3.37%) | 1.316 (0.421, 4.117) | 0.637 | 1.358 (0.433, 4.259) | 0.600 |
| **C21** | 135 | 1 (0.74%) | 0.287 (0.040, 2.047) | 0.213 | 0.308 (0.043, 2.200) | 0.240 |
| **C22** | 264 | 7 (2.65%) | 1.043 (0.490, 2.217) | 0.914 | 0.910 (0.427, 1.938) | 0.806 |
| **C23** | 359 | 6 (1.67%) | 0.637 (0.282, 1.435) | 0.276 | 0.630 (0.279, 1.422) | 0.266 |
| **C24** | 827 | 19 (2.30%) | 0.890 (0.555, 1.429) | 0.630 | 0.795 (0.495, 1.278) | 0.344 |
| **C25** | 114 | 1 (0.88%) | 0.339 (0.048, 2.419) | 0.281 | 0.360 (0.050, 2.571) | 0.308 |
| **C26** | 149 | 5 (3.36%) | 1.315 (0.541, 3.196) | 0.545 | 1.181 (0.484, 2.885) | 0.715 |
| **C27** | 144 | 3 (2.08%) | 0.813 (0.260, 2.542) | 0.722 | 0.914 (0.292, 2.867) | 0.878 |
| **C28** | 707 | 7 (0.99%) | 0.361 (0.170, 0.768) | 0.008 | 0.450 (0.211, 0.962) | 0.039 |
| **C29** | 214 | 5 (2.34%) | 0.912 (0.375, 2.216) | 0.839 | 0.964 (0.391, 2.378) | 0.937 |
| **C30** | 248 | 6 (2.42%) | 0.945 (0.419, 2.130) | 0.891 | 1.225 (0.541, 2.774) | 0.626 |

Adjust: Age, gender, hypertension, diabetes, coronary heart disease, peripheral arterial disease, hyperlipemia, smoke, drink, history of stroke

**Supplementary Table 11. Risk of poor functional outcome at 3 months in all clusters.**

| **Cluster** | **Number** | **Poor functional outcome at 3 months** | | | | |
| --- | --- | --- | --- | --- | --- | --- |
|  |  | **No. of Events (n, %)** | **OR (95% CI)** | **P value** | **Adjust OR (95% CI)** | **P value** |
| **C1** | 53 | 20 (37.74%) | 4.479(2.535, 7.917) | <0.001 | 4.479(2.237, 7.214) | <0.001 |
| **C2** | 70 | 25 (35.71%) | 3.743(2.284, 6.129) | <0.001 | 3.743(2.300, 6.347) | <0.001 |
| **C3** | 194 | 41 (21.13%) | 1.842(1.296, 2.620) | 0.001 | 1.842(1.426, 2.933) | <0.001 |
| **C4** | 308 | 101 (32.79%) | 3.603(2.809, 4.623) | <0.001 | 3.603(2.368, 3.955) | <0.001 |
| **C5** | 49 | 17 (34.69%) | 3.673(2.026, 6.659) | <0.001 | 3.673(2.030, 6.938) | <0.001 |
| **C6** | 88 | 8 (9.09%) | 0.667(0.321, 1.384) | 0.277 | 0.667(0.290, 1.269) | 0.185 |
| **C7** | 153 | 35 (22.88%) | 2.014(1.373, 2.956) | <0.001 | 2.014(1.052, 2.312) | 0.027 |
| **C8** | 81 | 7 (8.64%) | 0.623(0.286, 1.357) | 0.233 | 0.623(0.296, 1.429) | 0.284 |
| **C9** | 183 | 20 (10.93%) | 0.818(0.512, 1.309) | 0.402 | 0.818(0.610, 1.586) | 0.944 |
| **C10** | 211 | 16 (7.58%) | 0.541(0.323, 0.905) | 0.019 | 0.541(0.311, 0.879) | 0.014 |
| **C11** | 677 | 70 (10.34%) | 0.753(0.582, 0.973) | 0.031 | 0.753(0.526, 0.890) | 0.005 |
| **C12** | 991 | 109 (11.00%) | 0.808(0.654, 0.998) | 0.048 | 0.808(0.614, 0.951) | 0.016 |
| **C13** | 569 | 60 (10.54%) | 0.769(0.583, 1.013) | 0.062 | 0.769(0.543, 0.953) | 0.022 |
| **C14** | 125 | 13 (10.40%) | 0.801(0.449, 1.430) | 0.453 | 0.801(0.453, 1.464) | 0.491 |
| **C15** | 101 | 15 (14.85%) | 1.170(0.673, 2.034) | 0.578 | 1.170(0.690, 2.134) | 0.501 |
| **C16** | 128 | 17 (13.28%) | 1.013(0.605, 1.696) | 0.960 | 1.013(0.607, 1.716) | 0.939 |
| **C17** | 128 | 24 (18.75%) | 1.539(0.982, 2.411) | 0.060 | 1.539(1.124, 2.818) | 0.014 |
| **C18** | 158 | 16 (10.13%) | 0.763(0.453, 1.287) | 0.310 | 0.763(0.595, 1.723) | 0.964 |
| **C19** | 178 | 11 (6.18%) | 0.432(0.234, 0.799) | 0.007 | 0.432(0.252, 0.873) | 0.017 |
| **C20** | 89 | 12 (13.48%) | 1.031(0.559, 1.902) | 0.922 | 1.031(0.544, 1.883) | 0.969 |
| **C21** | 135 | 14 (10.37%) | 0.782(0.448, 1.366) | 0.387 | 0.782(0.456, 1.412) | 0.445 |
| **C22** | 264 | 45 (17.05%) | 1.396(1.005, 1.939) | 0.047 | 1.396(0.928, 1.809) | 0.128 |
| **C23** | 359 | 37 (10.31%) | 0.756(0.534, 1.069) | 0.114 | 0.756(0.544, 1.101) | 0.154 |
| **C24** | 827 | 78 (9.43%) | 0.675(0.529, 0.861) | 0.002 | 0.675(0.493, 0.808) | 0.000 |
| **C25** | 114 | 17 (14.91%) | 1.162(0.691, 1.954) | 0.571 | 1.162(0.710, 2.040) | 0.491 |
| **C26** | 149 | 32 (21.48%) | 1.836(1.235, 2.729) | 0.003 | 1.836(1.117, 2.522) | 0.013 |
| **C27** | 144 | 16 (11.11%) | 0.831(0.492, 1.404) | 0.488 | 0.831(0.553, 1.602) | 0.823 |
| **C28** | 707 | 61 (8.63%) | 0.605(0.462, 0.795) | 0.000 | 0.605(0.573, 0.998) | 0.048 |
| **C29** | 214 | 30 (14.02%) | 1.099(0.742, 1.627) | 0.636 | 1.099(0.778, 1.747) | 0.456 |
| **C30** | 248 | 33 (13.31%) | 1.021(0.703, 1.481) | 0.915 | 1.021(0.754, 1.606) | 0.619 |

Adjust: Age, gender, hypertension, diabetes, coronary heart disease, peripheral arterial disease, hyperlipemia, smoke, drink, history of stroke

**Supplementary Table 12. Proportion of variance explained by SNPs for each cluster.**

| **Cluster** | **Vg/Vp** | **SE** | **Vg** | **Vg_SE** | **Ve** | **Ve_SE** | **P value** |
| --- | --- | --- | --- | --- | --- | --- | --- |
| **1** | 0.0000 | 0.0370 | 0.0000 | 0.0003 | 0.0068 | 0.0003 | 0.500 |
| **2** | 0.0000 | 0.0372 | 0.0000 | 0.0003 | 0.0090 | 0.0004 | 0.500 |
| **3** | 0.0000 | 0.0363 | 0.0000 | 0.0009 | 0.0246 | 0.0010 | 0.500 |
| **4** | 0.0242 | 0.0359 | 0.0009 | 0.0014 | 0.0375 | 0.0015 | 0.239 |
| **5** | 0.0000 | 0.0371 | 0.0000 | 0.0002 | 0.0063 | 0.0003 | 0.500 |
| **6** | 0.0000 | 0.0341 | 0.0000 | 0.0004 | 0.0113 | 0.0004 | 0.500 |
| **7** | 0.0895 | 0.0342 | 0.0017 | 0.0007 | 0.0177 | 0.0007 | 0.000 |
| **8** | 0.0091 | 0.0349 | 0.0001 | 0.0004 | 0.0103 | 0.0004 | 0.391 |
| **9** | 0.0920 | 0.0369 | 0.0021 | 0.0009 | 0.0211 | 0.0009 | 0.002 |
| **10** | 0.0138 | 0.0324 | 0.0004 | 0.0009 | 0.0263 | 0.0010 | 0.312 |
| **11** | 0.0914 | 0.0387 | 0.0073 | 0.0031 | 0.0730 | 0.0033 | 0.008 |
| **12** | 0.0527 | 0.0351 | 0.0059 | 0.0039 | 0.1063 | 0.0042 | 0.043 |
| **13** | 0.0466 | 0.0382 | 0.0032 | 0.0026 | 0.0653 | 0.0028 | 0.110 |
| **14** | 0.0922 | 0.0334 | 0.0015 | 0.0005 | 0.0145 | 0.0006 | <0.001 |
| **15** | 0.1358 | 0.0342 | 0.0018 | 0.0004 | 0.0112 | 0.0005 | <0.001 |
| **16** | 0.0000 | 0.0378 | 0.0000 | 0.0006 | 0.0163 | 0.0007 | 0.500 |
| **17** | 0.0208 | 0.0375 | 0.0003 | 0.0006 | 0.0160 | 0.0007 | 0.289 |
| **18** | 0.0337 | 0.0391 | 0.0007 | 0.0008 | 0.0194 | 0.0008 | 0.203 |
| **19** | 0.0000 | 0.0369 | 0.0000 | 0.0008 | 0.0226 | 0.0009 | 0.500 |
| **20** | 0.0000 | 0.0361 | 0.0000 | 0.0004 | 0.0114 | 0.0004 | 0.500 |
| **21** | 0.0000 | 0.0376 | 0.0000 | 0.0006 | 0.0172 | 0.0007 | 0.500 |
| **22** | 0.0056 | 0.0362 | 0.0002 | 0.0012 | 0.0330 | 0.0013 | 0.438 |
| **23** | 0.0856 | 0.0389 | 0.0038 | 0.0017 | 0.0407 | 0.0018 | 0.013 |
| **24** | 0.0465 | 0.0363 | 0.0045 | 0.0035 | 0.0915 | 0.0037 | 0.080 |
| **25** | 0.0000 | 0.0379 | 0.0000 | 0.0006 | 0.0146 | 0.0006 | 0.500 |
| **26** | 0.0467 | 0.0351 | 0.0009 | 0.0007 | 0.0181 | 0.0007 | 0.066 |
| **27** | 0.0256 | 0.0380 | 0.0005 | 0.0007 | 0.0179 | 0.0007 | 0.253 |
| **28** | 0.0000 | 0.0370 | 0.0000 | 0.0031 | 0.0834 | 0.0033 | 0.500 |
| **29** | 0.0537 | 0.0376 | 0.0015 | 0.0010 | 0.0256 | 0.0011 | 0.066 |
| **30** | 0.0064 | 0.0374 | 0.0002 | 0.0012 | 0.0310 | 0.0013 | 0.433 |

**Supplementary Table 13. Genome-wide significant risk loci for each cluster.**

| **Cluster** | **SNP** | **CHR** | **BP** | **GENE** | **ALT** | **REF** | **MAF** | **OR** | **STAT** | **NMISS** | **P** | **ANNO** |
| --- | --- | --- | --- | --- | --- | --- | --- | --- | --- | --- | --- | --- |
| **1** | rs4688534 | 3 | 65037452 | ADAMTS9-AS2 | C | A | 0.0603 | A | 31.05 | 0.05941 | 2.52E-08 | intron_variant,non_coding_transcript_variant |
| **6** | rs75943769 | 5 | 153126504 | LINC01470 | C | T | 0.05605 | T | 35.73 | 0.05485 | 2.27E-09 | intron_variant,non_coding_transcript_variant |
| **7** | rs6798807 | 3 | 14130575 | TMEM43 | T | C | 0.3288 | C | 29.76 | 0.3259 | 4.90E-08 | intron_variant,NMD_transcript_variant |
| **8** | chr2:75827282:T:TTAAATGTAAGCTCTAAAACTAT | 2 | 75827282 | AC110614 | T | TTAAATGTAAGCTCTAAAACTATA | 0.0595 | TTAAATGTAAGCTCTAAAACTATA | 41.8 | 0.05822 | 1.01E-10 |  |
| **8** | rs6118070 | 20 | 8113306 | PLCB1 | C | T | 0.1762 | T | 32.38 | 0.1744 | 1.27E-08 | upstream_gene_variant,intron_variant |
| **8** | rs145811063 | 17 | 75837135 | UNC13D | A | G | 0.05281 | G | 29.75 | 0.05179 | 4.91E-08 | intron_variant,NMD_transcript_variant,upstream_gene_variant,downstream_gene_variant,non_coding_transcript_variant |
| **9** | rs1801133 | 1 | 11796321 | MTHFR | G | A | 0.4573 | A | 180.1 | 0.4657 | 4.62E-41 | 3_prime_UTR_variant,intron_variant,NMD_transcript_variant,downstream_gene_variant,non_coding_transcript_exon_variant,5_prime_UTR_variant,non_coding_transcript_variant,missense_variant |
| **9** | rs12038287 | 1 | 11835339 | CLCN6 | C | T | 0.433 | T | 89.76 | 0.4271 | 2.69E-21 | intron_variant,NMD_transcript_variant,upstream_gene_variant,downstream_gene_variant,non_coding_transcript_variant |
| **9** | rs12092731 | 1 | 11908338 | AL021155 | C | T | 0.4811 | T | 50.47 | 0.4856 | 1.21E-12 | downstream_gene_variant |
| **9** | rs2639453 | 1 | 11922487 | KIAA2013 | T | C | 0.3646 | C | 44.33 | 0.3605 | 2.78E-11 | 3_prime_UTR_variant,intron_variant |
| **9** | rs6684539 | 1 | 11908216 | RNU5E-1 | A | G | 0.4837 | G | 43.05 | 0.4795 | 5.33E-11 | non_coding_transcript_exon_variant,downstream_gene_variant |
| **9** | rs4845892 | 1 | 12013728 | MFN2 | C | A | 0.4625 | A | 37.52 | 0.4586 | 9.05E-10 | intron_variant,downstream_gene_variant |
| **9** | rs11121846 | 1 | 11970390 | PLOD1 | G | A | 0.4641 | A | 34.22 | 0.4604 | 4.93E-09 | upstream_gene_variant,intron_variant,non_coding_transcript_variant,downstream_gene_variant |
| **9** | rs6697244 | 1 | 11778791 | C1orf167-AS1 | G | T | 0.162 | T | 33.11 | 0.1647 | 8.70E-09 | missense_variant,upstream_gene_variant,non_coding_transcript_exon_variant,downstream_gene_variant |
| **9** | rs34954501 | 1 | 11878146 | SBF1P2 | G | A | 0.4842 | A | 31.76 | 0.4878 | 1.74E-08 | non_coding_transcript_exon_variant |
| **9** | rs926271 | 1 | 11909860 | RNU5E-4P | G | A | 0.4592 | A | 30.54 | 0.4626 | 3.27E-08 | non_coding_transcript_exon_variant,downstream_gene_variant |
| **12** | rs1208125736 | 13 | 18212172 | FAM230C | G | A | 0.3517 | A | 161.3 | 0.3705 | 5.78E-37 | intron_variant,non_coding_transcript_variant |
| **15** | rs2831790 | 21 | 28418816 | AF165147 | G | A | 0.08922 | A | 29.82 | 0.08777 | 4.74E-08 |  |
| **19** | rs111784051 | 19 | 44899005 | TOMM40 | G | T | 0.07786 | T | 100.8 | 0.07451 | 1.01E-23 | intron_variant,NMD_transcript_variant,downstream_gene_variant |
| **19** | rs72654473 | 19 | 44911142 | AC011481 | A | C | 0.07682 | C | 95.97 | 0.07358 | 1.17E-22 | upstream_gene_variant,downstream_gene_variant |
| **19** | rs651821 | 11 | 116791863 | APOA5 | C | T | 0.2743 | T | 73.58 | 0.2695 | 9.66E-18 | 5_prime_UTR_variant,upstream_gene_variant |
| **19** | rs7254892 | 19 | 44886339 | NECTIN2 | A | G | 0.05767 | G | 70.38 | 0.05524 | 4.88E-17 | upstream_gene_variant,intron_variant |
| **19** | rs964184 | 11 | 116778201 | ZPR1 | G | C | 0.2183 | C | 39.32 | 0.215 | 3.59E-10 | 3_prime_UTR_variant,upstream_gene_variant,downstream_gene_variant |
| **19** | rs11568822 | 19 | 44914381 | APOC1 | CTTCG | C | 0.1816 | C | 36.39 | 0.1787 | 1.61E-09 | 5_prime_UTR_variant,upstream_gene_variant,downstream_gene_variant |
| **19** | rs3825041 | 11 | 116760991 | BUD13 | T | C | 0.2115 | C | 36.03 | 0.2084 | 1.94E-09 | intron_variant,NMD_transcript_variant |
| **24** | rs12170409 | 22 | 49925087 | CRELD2 | T | C | 0.09563 | C | 31.27 | 0.09104 | 2.25E-08 | non_coding_transcript_exon_variant,intron_variant,non_coding_transcript_variant,downstream_gene_variant |
| **25** | rs73596816 | 6 | 160596331 | LPA | A | G | 0.05154 | G | 45.08 | 0.05007 | 1.89E-11 | intron_variant |
| **25** | rs56393506 | 6 | 160668275 | AL109933 | T | C | 0.1186 | C | 38.23 | 0.1166 | 6.30E-10 | upstream_gene_variant |
| **27** | rs28946889 | 2 | 233762816 | UGT1A4 | T | G | 0.4098 | G | 67.69 | 0.4143 | 1.91E-16 | intron_variant,NMD_transcript_variant |
| **27** | rs10929285 | 2 | 233705031 | AC114812 | T | G | 0.4168 | G | 61.59 | 0.4211 | 4.23E-15 | intron_variant,NMD_transcript_variant,non_coding_transcript_variant |
| **27** | rs11888459 | 2 | 233747994 | UGT1A2P | C | T | 0.1243 | T | 57.93 | 0.1215 | 2.71E-14 | intron_variant,NMD_transcript_variant,upstream_gene_variant,non_coding_transcript_exon_variant,non_coding_transcript_variant |
| **27** | rs7581102 | 2 | 233786081 | MROH2A | A | G | 0.11 | G | 37.78 | 0.1078 | 7.90E-10 | intron_variant,non_coding_transcript_variant |
| **27** | rs7597496 | 2 | 233721797 | RPL17P11 | G | A | 0.2995 | A | 33.78 | 0.2965 | 6.16E-09 | non_coding_transcript_exon_variant,intron_variant,NMD_transcript_variant,non_coding_transcript_variant |
| **27** | rs2269352 | 2 | 233604330 | UGT1A11P | C | T | 0.4928 | T | 31.19 | 0.496 | 2.34E-08 | non_coding_transcript_exon_variant |
| **29** | rs585508 | 1 | 201582028 | RPS10P7 | A | G | 0.138 | G | 33.83 | 0.1353 | 6.00E-09 | intron_variant,non_coding_transcript_variant |
| **29** | rs4754221 | 11 | 107416923 | CWF19L2 | G | A | 0.1307 | A | 30.68 | 0.1281 | 3.04E-08 | intron_variant,NMD_transcript_variant,non_coding_transcript_variant |

**Supplementary Table 14. Seven mutations were predicted as deleterious or damaging for clusters.**

| **Cluster** | **SNPs** | **SYMBOL** | **SIFT** | **SIFTScore** | **Polyphen** | **PolyphenScore** | **p** |
| --- | --- | --- | --- | --- | --- | --- | --- |
| 1 | rs9603837 | ADPRHL1 | deleterious_  low_confidence | 0 | unknown | 0 | 8.79E-06 |
| 9 | rs1023252 | CLCN6 | deleterious_l  ow_confidence | 0 | unknown | 0 | 4.73E-12 |
| 9 | rs61773952 | C1orf167 | deleterious | 0.01 | probably_  damaging | 0.974 | 9.35E-06 |
| 26 | rs77073793 | C8orf74 | deleterious | 0.04 | possibly_  damaging | 0.833 | 5.65E-06 |
| 27 | rs1105879 | UGT1A6 | deleterious | 0.02 | benign | 0.103 | 5.18E-09 |
| 27 | rs4148323 | UGT1A1 | deleterious | 0.05 | possibly_  damaging | 0.625 | 2.53E-08 |
| 27 | rs1042597 | UGT1A8 | deleterious | 0 | benign | 0.116 | 3.64E-08 |

**Supplementary Table 15. Colocalization of biomarkers and clusters.**

| **rsID** | **Biomarker** | **Cluster** | **SNP.PP.H4** | **pval_grp** | **pval_bio** | **Gene Symbol** |
| --- | --- | --- | --- | --- | --- | --- |
| rs1801133 | HCY | 9 | 1 | 4.622E-41 | 7.038E-160 | MTHFR |
| rs651821 | Apo-E | 19 | 0.90996056 | 9.656E-18 | 2.051E-26 | APOA5 |
| rs651821 | Triglyceride | 19 | 0.64583626 | 9.656E-18 | 1.076E-52 | APOA5 |
| rs111784051 | Apo-E | 19 | 0.96062011 | 1.011E-23 | 1.182E-108 | TOMM40 |
| rs111784051 | Triglyceride | 19 | 0.67616134 | 1.011E-23 | 1.539E-12 | TOMM40 |
| rs72654473 | Apo-B | 19 | 0.56845922 | 1.165E-22 | 9.002E-12 | AC011481 |
| rs28946889 | DBIL | 27 | 0.9999992 | 1.912E-16 | 1.197E-45 | UGT1A1 |
| rs28946889 | IBIL | 27 | 1 | 1.912E-16 | 1.031E-93 | UGT1A1 |
| rs10929285 | DBIL | 27 | 0.99984217 | 4.228E-15 | 7.07E-39 | AC114812 |
| rs10929285 | IBIL | 27 | 1 | 4.228E-15 | 3.555E-79 | AC114812 |
